# Radical TADF: Quartet‐Derived Luminescence with Dark TEMPO

**DOI:** 10.1002/adma.202501164

**Published:** 2025-05-15

**Authors:** Sebastian Gorgon, Petri Murto, Daniel G. Congrave, Lujo Matasovic, Andrew D. Bond, Victor Riesgo‐Gonzalez, William K. Myers, Hugo Bronstein, Richard H. Friend

**Affiliations:** ^1^ Cavendish Laboratory University of Cambridge JJ Thomson Ave Cambridge CB3 0US UK; ^2^ Centre for Advanced Electron Spin Resonance Department of Chemistry University of Oxford Inorganic Chemistry Laboratory S Parks Rd Oxford OX1 3QR UK; ^3^ Yusuf Hamied Department of Chemistry University of Cambridge Lensfield Rd Cambridge CB2 1EW UK; ^4^ Department of Chemistry and Materials Science Aalto University Kemistintie 1 Espoo 02150 Finland; ^5^ Department of Chemistry University of Oxford Chemistry Research Laboratory Oxford OX1 3TA UK

**Keywords:** electron spin, luminescence, optoelectronics, organic chromophores, quartet states, TADF

## Abstract

High‐spin states in organic molecules offer promising tuneability for quantum technologies. Photogenerated quartet excitons are an extensively studied platform, but their applications are limited by the absence of optical read‐out via luminescence. Here, a new class of synthetically accessible molecules with quartet‐derived luminescence is demonstrated, formed by appending a non‐luminescent TEMPO radical to thermally activated delayed fluorescence (TADF) chromophores previously used in OLEDs. The low singlet‐triplet energy gap of the chromophore opens a luminescence channel from radical‐triplet coupled states. A set of design rules is established by tuning the energetics in a series of compounds based on a naphthalimide (NAI) core. Generation of quartet states is observed and the strength of radical‐triplet exchange is measured. In DMAC‐TEMPO, up to 72% of detected photons emerge after reverse intersystem crossing from the quartet state repopulates the state with singlet character. This design strategy does not rely on a luminescent radical to provide an emission pathway from the high‐spin state. The large library of TADF chromophores promises a greater pallet of achievable emission colours.

## Graphical abstract

A new class of luminescent organic quantum materials is introduced, formed by attaching a stable radical to TADF chromophores. Due to ferromagnetic exchange coupling, a high‐spin quartet state forms upon excitation, which can thermally access a bright state. ≈200 nm tunability of the emission color is reported, and coherent spin manipulations with microwaves are achieved.

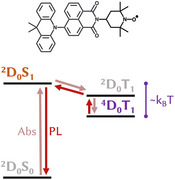

## 1 Introduction

Organic molecules are an attractive platform for quantum technologies.^[^ ^1^ ^]^ They offer a highly modular and deterministic approach for constructing precise quantum objects via chemical synthesis.^[^ ^2^ ^]^ The absence of heavy atoms offers further advantages in biocompatibility, increasing the scope for future quantum sensing demonstrations at physiological conditions.

While controlled preparation and manipulation of electron spins in organics is well established, engineering of efficient optical read‐out pathways remains a challenge. Light provides the most facile and non‐invasive way to read‐out a spin system,^[^ ^3^ ^]^ which would bring organics closer to practical applications.

**Figure 1:**
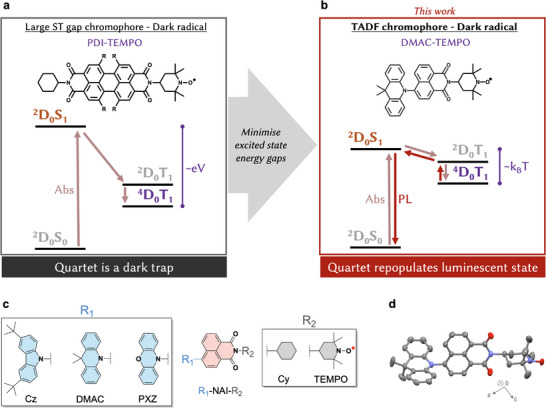
Linking high‐spin and luminescent states in presence of TEMPO radicals. Simplified Jabłoński diagrams for organic molecules with quartet, ^4^D_0_T_1_, excited states. a) Structures based on dark radicals reported to date, such as PDI‐TEMPO, have large singlet‐triplet (ST) gaps, therefore the luminescent ^2^D_0_S_1_ is inaccessible from the quartet. b) In the new strategy presented here, we instead use a TADF chromophore with a small ST gap. The luminescent ^2^D_0_S_1_ is thermally accessible from the quartet and delayed emission is observed, despite the radical being non‐luminescent. c) Molecular structures explored for the TADF‐TEMPO strategy consist of an NAI core coupled to R_1_, an electron‐donating unit: either Cz‐, DMAC‐, or PXZ‐. We study the radical and non‐radical (‐Cy) compounds, the latter acting as a reference. d) Molecular structure of DMAC‐TEMPO taken from the X‐ray crystal structure, revealing the large dihedral angle between the DMAC and NAI units (82.4°).

Key demonstrations of information transfer, storage, and manipulation have been made in excited‐state multi‐spin organic molecules.^[^ ^4^, ^5^, ^6^, ^7^ ^]^ In particular, three‐spin systems with quartet (*S* = 3/2) excited states have been explored as a versatile organic qubit platform.^[^ ^8^, ^9^ ^]^ To date they were obtained by appending a stable, non‐luminescent radical to a simple chromophore (**Figure** **1**a).^[^ ^10^ ^]^ These quartet candidates contain chromophore units with a locally excited (LE)‐type emissive state. Due to large excited‐state singlet‐triplet (S_1_‐T_1_) energy gaps, once a quartet state is formed, it cannot repopulate the initially photogenerated state. Thus, optical read‐out of the high‐spin state via photoluminescence is not available in such structures.

Recently, we have shown an alternative approach to constructing quartet‐bearing molecules. By employing a luminescent π‐conjugated radical and engineering appropriate energy level resonances, our design reversibly linked the quartet state with a doublet state emitting near 700 nm.^[^ ^11^ ^]^ However, relying on luminescent radicals may restrict the range of emission wavelength tunability,^[^ ^12^ ^]^ and limit structural and synthetic diversity.

Stable nitroxide radicals like 2,2,6,6‐tetramethyl‐1‐piperidinyloxy (TEMPO) are widely‐studied open‐shell systems, as they are robust in chemical synthesis, electrochemical applications, and under light‐excitation.^[^ ^13^, ^14^ ^]^ TEMPO has been incorporated into a wide range of chemical structures through mild chemistry that circumvents the need for additional radical conversion steps, making it structurally diverse and synthetically accessible.^[^ ^9^ ^]^ However, as typical of σ‐radicals, TEMPO acts as a luminescence quencher.^[^ ^15^ ^]^ Existing reports of light emission in TEMPO‐derived species rely on harsh redox reactions or aggregation‐based strategies.^[^ ^16^, ^17^ ^]^

TADF is currently a central direction in organic optoelectronics research.^[^ ^18^, ^19^ ^]^ This allows us to take advantage of the vast array of TADF structures to unlock a new direction for luminescent high‐spin molecules. As established for π‐conjugated radicals, reversibly linking the quartet state with an emissive state requires minimizing their energy offset. In TADF‐TEMPO structures, the small energy offset requirement is automatically fulfilled due to the small S_1_‐T_1_ gap inherent to TADF materials (Figure 1b). The additional radical‐triplet exchange only calls for an interaction on the order of a few µeV, to ensure that a coupled quartet state is formed. As the emissive channel is provided by the TADF chromophore, we do not require a luminescent radical.

We focus on an efficient TADF chromophore operating near 2.0 eV with a high intersystem crossing yield, which is a donor‐acceptor system with strong charge transfer (CT) character.^[^ ^20^ ^]^ The design consists of an electron‐accepting 1,8‐naphthalimide (NAI) core with its nitrogen substituted with either a cyclohexane (Cy) or near‐isostructural TEMPO radical as R_2_ (Figure 1c). To explore the sensitivity of the mechanism to the energetics, we prepare a series of structures with three different electron‐donating units R_1_ coupled at the 4‐position: 3,6‐di‐*tert*‐butylcarbazole (Cz),^[^ ^21^ ^]^ 9,9‐dimethyl‐9,10‐dihydroacridine (DMAC),^[^ ^20^ ^]^ and phenoxazine (PXZ),^[^ ^22^ ^]^ in decreasing order of CT energy. The versatility of the DMAC‐NAI motif has been recently shown in bioimaging applications.^[^ ^23^ ^]^

## 2 Results

Both the DMAC‐substituted molecules, DMAC‐Cy and DMAC‐TEMPO, and the PXZ‐substituted molecules, PXZ‐Cy and PXZ‐TEMPO, were synthesized via condensation chemistry followed by Buchwald–Hartwig amination using a palladium catalyst and a bulky alkoxide base with yields varying between 60% and 90%.^[^ ^24^, ^25^ ^]^ The Cz donor was less reactive in these conditions giving low yields (5% or less). Synthesis of Cz‐Cy and Cz‐TEMPO required deprotonation of the Cz nitrogen with a Grignard reagent, and these molecules were isolated with yields of 20–30%.^[^ ^26^ ^]^ NMR spectroscopy suggests that coupling of the radical electron to the NAI hydrogen nuclei is stronger than its coupling to the three donors’ hydrogen nuclei, which is observed as significant broadening of the ^1^H NMR signals of the NAI unit rather than those of the donor units (Figure S1, Supporting Information). The NMR data can be rationalized by considering that the radical is directly linked to the acceptor, whereas interactions between the radical electron and the relevant donor nuclei are weakened by the large dihedral, as shown for DMAC‐TEMPO in Figure 1d. Detailed synthetic protocols, NMR spectra, thermal gravimetry analysis, and X‐ray crystal structures are provided (Figures S2–S12, Supporting Information).

**Figure 2:**
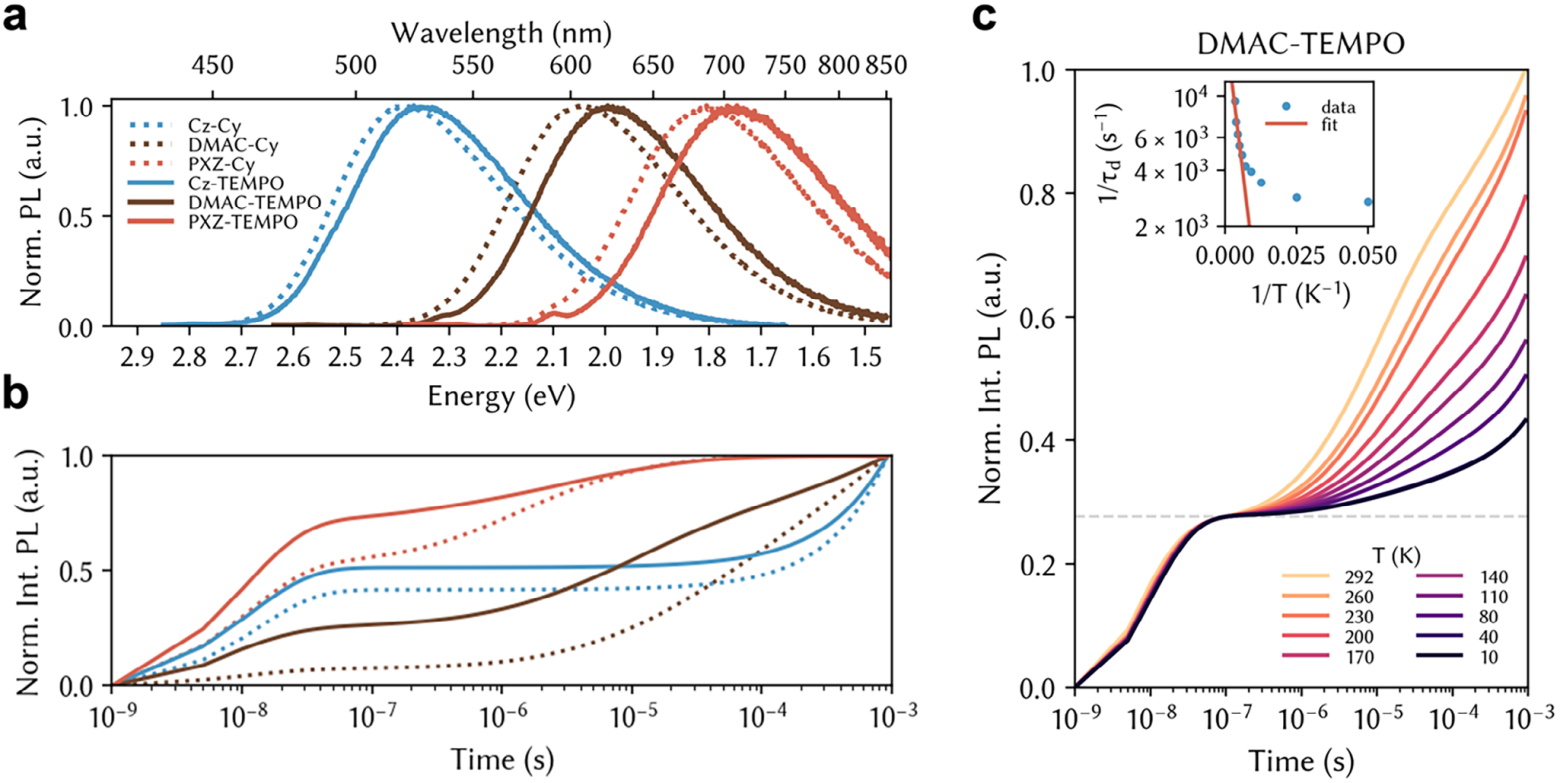
Luminescence spectroscopy. a) Steady‐state photoluminescence spectra in 100 µm toluene solutions following near band‐edge excitation at 295 K. b) Integrated kinetic traces extracted from transient photoluminescence of 1% PMMA films at 293 K under vacuum after 450 nm excitation. c) Temperature dependence of emission intensity in 1% DMAC‐TEMPO in PMMA film under 400 nm excitation. Luminescence was integrated across the 550–700 nm spectral region. Inset shows the Arrhenius fit which extracts an activation energy *E*_A_ = 39 ± 8 meV.

CT‐type photoluminescence is observed for all 6 compounds (**Figure** **2**a), as expected for this TADF motif.^[^ ^20^, ^27^ ^]^ The emission lineshapes do not significantly evolve as function of time after photoexcitation at room temperature (Figure S15, Supporting Information), suggesting the PL arises from a single excited state in each molecule. For the Cy‐derivatives, the emission wavelengths at room temperature in toluene solutions follow expected red‐shifts with increasing strength of the donor moiety R_1_, spanning a range of 0.6 eV. The corresponding TEMPO‐derivatives show a very small (ca. 40 meV) redshift. Cyclic voltammograms (Figure S13, Supporting Information) and absorption spectra (Figure S14, Supporting Information) are consistent with these trends in the emission spectra. Substituting TEMPO onto the acceptor results in a <0.1 V shift of its reduction to a less negative potential (deeper LUMO energy), whereas increasing the donor strength pushes the oxidation to a less positive potential (shallower HOMO energy). Hence, substituting TEMPO and increasing the donor strength both have the effect of lowering the HOMO‐LUMO energy gap.

DMAC‐Cy shows the highest proportion of delayed fluorescence, with over 90% of all emitted photons arriving via delayed emission in 1% in PMMA films (**Table** **1**). Its PLQE of 35% is expected given the low emission energy and is consistent with similar derivatives synthesized to‐date.^[^ ^20^, ^28^ ^]^ DMAC‐TEMPO preserves these dynamics (Figure 2b). As in other radical chromophores, luminescence quenching is observed at high concentrations both in the solid (Figure S20, Supporting Information) and liquid (Figure S21, Supporting Information) state.

**Table 1:** Luminescence properties at 292 K. Emission peak energy (E_PL_) and wavelength (λ_PL_), and quantum yield (PLQE) were measured in 100 µm toluene solutions under near‐band‐gap cw excitation. Delayed emission fraction (I_d_/I_tot_), as well as prompt (τ_p_) and delayed (τ_d_) lifetimes were measured in 1% in PMMA films under vacuum under 450 nm pulsed excitation.

Material	E_PL_ [eV]	λ_PL_ [nm]	PLQE_tol_ [%]	PLQE_PMMA_ [%]	I_d_/I_tot_ [%]	τ_p_ [s]	τ_d_ [s]
Cz‐Cy	2.39	519	22	29	56	1.4 × 10^−8^	1.2 × 10^−2^
DMAC‐Cy	2.04	608	35	35	92	1.9 × 10^−8^	1.7 × 10^−5^
PXZ‐Cy	1.81	685	<1	4	42	1.4 × 10^−8^	2.3 × 10^−6^
Cz‐TEMPO	2.35	528	1	11	49	1.2 × 10^−8^	3.2 × 10^−3^
DMAC‐TEMPO	1.99	623	4	10	72	1.3 × 10^−8^	7.0 × 10^−6^
PXZ‐TEMPO	1.75	708	<1	2	28	1.2 × 10^−8^	4.2 × 10^−6^

To investigate the energetic landscape around the emissive state, we perform temperature‐dependent luminescence spectroscopy. In DMAC‐TEMPO, the fast emission component has a lifetime of ca. 13 ns and contributes ≈27% of all emitted photons across the entire 10–292 K temperature range (Figure 2c). This indicates it is due to ^2^D_0_S_1_^(CT)^ prompt fluorescence in conformations where this outcompetes quartet state generation. The delayed emission in DMAC‐TEMPO is strongly temperature‐activated, with delayed emission intensity ≈5 times larger at room temperature compared to 10 K. The emission lineshape and time‐dependence of emission wavelength do not depend on temperature. This confirms that luminescence occurs from the same emissive state at all temperatures. Arrhenius analysis reveals that this ^2^D_0_S_1_^(CT)^ state can be reformed after crossing an activation barrier of 39 ± 8 meV, which is ≈1.5k_B_T at room temperature. This is similar to the literature values for the activation energy on the related radical‐free DMAC‐NAI TADF compounds.^[^ ^29^, ^30^ ^]^

Turning to Cz‐TEMPO, at 10 K we observe a ms lifetime emission which is redshifted by ≈0.4 eV from the prompt luminescence and exhibits a pronounced vibronic structure (Figure S16, Supporting Information). We thus assign it to phosphorescence from the NAI ^3^LE‐character state, consistent with the literature.^[^ ^27^ ^]^ As this structural motif is common across all 6 compounds, this measurement indicates its energetic position for the whole series. This confirms that the NAI ^3^LE‐character state is near‐isoenergetic with the CT character states in both DMAC‐Cy and DMAC‐TEMPO.

We track the exciton populations via ultrafast transient absorption (TA). Upon photoexcitation, the spectra are dominated by photoinduced absorptions (PIA) near 425 and 720 nm, consistent with literature values for NAI anions (Figure S17, Supporting Information).^[^ ^31^ ^]^ These initially arise from the photogenerated S_1_^(CT)^ in the Cy derivatives and ^2^D_0_S_1_^(CT)^ in the TEMPO derivatives. However, due to small ST gaps, at later times the same PIAs may also arise from any states with triplet CT character. These then evolve toward a PIA near 480 nm which corresponds to the LE NAI triplet.^[^ ^32^ ^]^ We observe an acceleration in the CT PIA depopulation when TEMPO is appended, indicating that the presence of the radical is enhancing intersystem crossing. These dynamics are consistent with the observation of weak prompt fluorescence.

**Figure 3:**
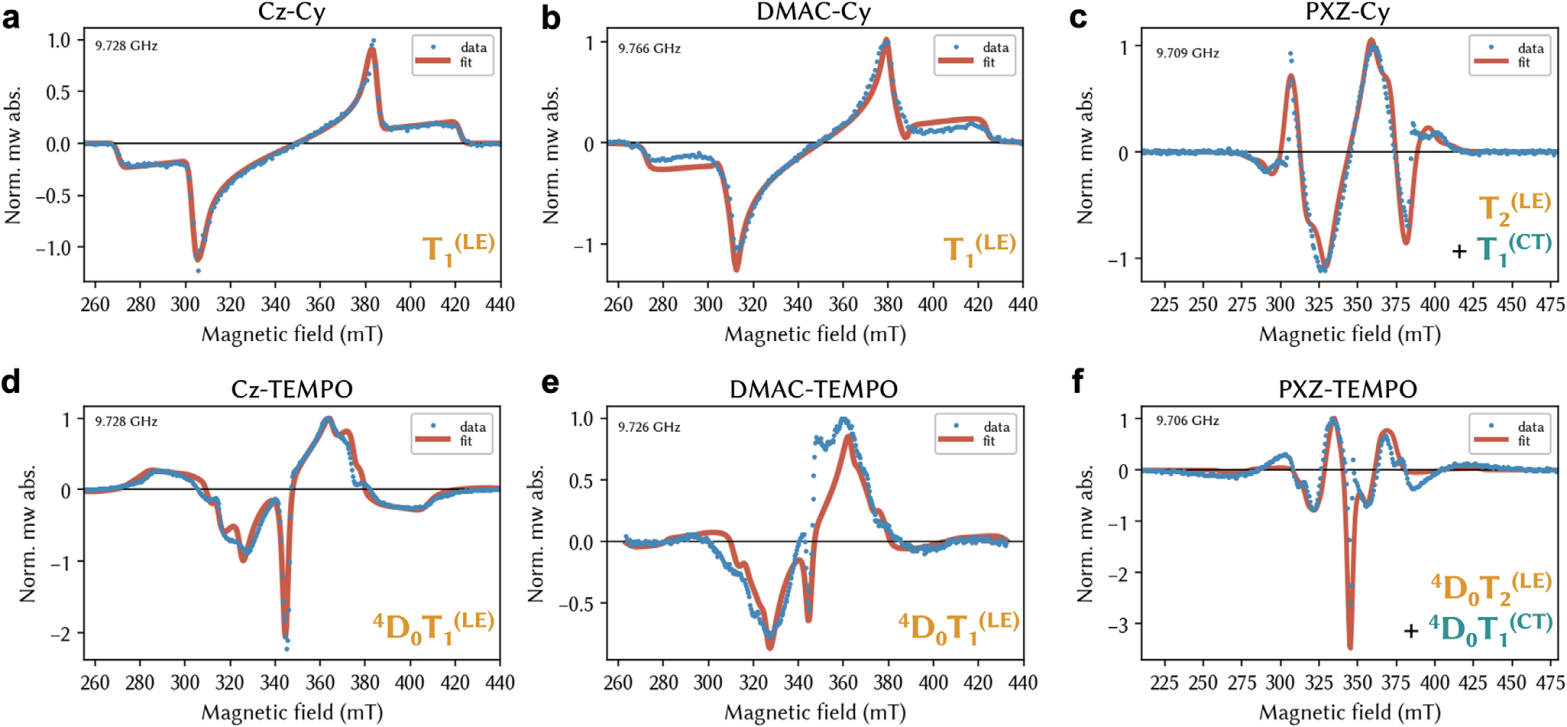
Transient ESR. Prompt (1µs) trESR spectra on frozen 100 µm toluene solutions collected at 80 K at X‐band under excitation with 450 nm 1 mJ pulses. The radical‐free TADF reference compounds show triplet signatures with a) Cz‐Cy and b) DMAC‐Cy spectra displaying an EEEAAA polarization pattern with near identical turning points. c) PXZ‐Cy spectrum is a linear combination of two triplet signatures. The TEMPO‐substituted series spectra arise from quartet states in the intermediate exchange coupling regime. d) Cz‐TEMPO and e) DMAC‐TEMPO spectra have a similar lineshape, while f) PXZ‐TEMPO spectrum reveals population of two quartet species.

Transient ESR (trESR) is a powerful method to study multispin excitons in organic optoelectronic materials.^[^ ^33^, ^34^ ^]^ The spectra are rich in information on the shape and character of the wavefunction, while the extracted sublevel polarization pattern can directly determine the type of ISC mechanism.^[^ ^35^ ^]^ X‐band trESR following 450 nm excitation at 80 K reveals spin‐polarized signals in all six materials studied here. Starting with the radical‐free TADF compounds, Cz‐Cy (**Figure** **3**a) and DMAC‐Cy (Figure 3b) show similar signals with a characteristic polarization pattern of ISC‐generated triplets. Simulation reveals these to have similar dipolar coupling parameters (**Table** **2**). Given the energetics extracted from our luminescence results, we assign this state to the ^3^LE. This matches zfs splittings in the NAI literature.^[^ ^28^, ^30^, ^32^, ^36^ ^]^ A more complex spectrum is seen for PXZ‐Cy (Figure 3c). As indicated by the time evolution of the signals (Figure S18, Supporting Information), we can decompose it into a linear combination of the same ^3^LE seen in the other two derivatives, and a second triplet (Figure S19, Supporting Information). The second triplet has a smaller *D* coupling and larger *D/E* ratio, indicating a greater electron‐hole separation and a more oblong spin distribution than the ^3^LE. Together with the polarization pattern,^[^ ^28^ ^]^ this suggests it arises from a ^3^CT, with hole on PXZ and electron on NAI.

**Table 2:** Spin Hamiltonian parameters. Radical‐free compounds were simulated as triplets (*S* = 1) in the zero‐field basis. The TEMPO‐derivatives were simulated in the coupled basis (*S*_1_ = 1, *S*_2_ = 1/2).

Material	T_n_	Weight		|*D*_T_|, |*E*_T_| [MHz]	P_x_:P_y_:P_z_
Cz‐Cy	LE	100%		2133, 53	0.53:0.33:0.14
DMAC‐Cy	LE	100%		2123, 85	0.24:0.72:0.04
PXZ‐Cy	LE	88%		1920, 120	0.28:0.39:0.33
	CT	12%		1515, 236	0.15:0.66:0.19

Material	T_n_	Weight	|*J*_TR_| [GHz]	|*D*_T_|, |*E*_T_| [MHz]	Q_+3/2_:Q_+1/2_:Q_‐1/2_:Q_‐3/2_:D_+1/2_:D_‐1/2_
Cz‐TEMPO	LE	100%	0.70	2120, 53	0.19:0.09:0.14:0.19:0.21:0.19
DMAC‐TEMPO	LE	100%	0.65	1923, 130	0.21:0.08:0.13:0.22:0.19:0.17
PXZ‐TEMPO	LE	43%	0.68	1904, 146	0.21:0.08:0.14:0.21:0.19:0.17
	CT	57%	>20	1264, 120	0.25:0.12:0.09:0.21:0.04:0.28

Turning to the TEMPO‐derivatives, we observe intense and complex spin‐polarized lineshapes in all three compounds. They share the following features: a narrow emissive line is present near *g* = 2; the most intense turning points are narrower, and the full width of the spectrum is broader than the matching Cy‐reference compounds. Analogously to the radical‐free compounds, the prompt spectrum can be simulated with 1 quartet state for Cz‐TEMPO (Figure 3d) and DMAC‐TEMPO (Figure 3e), but requires 2 for PXZ‐TEMPO (Figure 3f). Simulations reveal that in Cz‐TEMPO and DMAC‐TEMPO these arise from quartet states in the intermediate exchange coupling regime between the triplet and radical. This permits us to directly determine *J_TR_*, which is near 0.7 GHz for all quartet states arising from the coupling of the LE triplet to the TEMPO radical. Despite this value of *J*, already the X‐band spectra are symmetric, since level crossings occur below 100 mT. The optimal zfs values are similar to those found in respective reference Cy‐derivatives, suggesting small triplet‐radical dipolar coupling *D*_TR_. We confirm our spectral assignments by performing trESR at Q‐band on Cz‐TEMPO (Figure S20, Supporting Information), and by explicitly calculating the spectral lineshape for a range of *J_TR_* values (Figure S21, Supporting Information). The two species present in PXZ‐TEMPO evolve with different kinetics, and the delayed polarization shifts toward the CT‐born quartet whose lineshape is narrower by a factor of 2/3 compared to the closed‐shell CT triplet. Simulations confirm that this CT quartet is in the strong regime of triplet‐radical exchange coupling. This is consistent with the greater asymmetry of the pairwise coupling constants of the two triplet spins to TEMPO, due to their spatial separation on the donor and NAI units. The LE‐born quartet in the intermediate coupling regime also contributes to the PXZ‐TEMPO signal, since as in the other derivatives both of its triplet spins reside on the NAI unit. We stress that obtaining the spin Hamiltonian parameters of the parent triplets through studying the Cy‐substituted isostructural analogues provides confidence in assigning the complex quartet spectra seen in our TEMPO‐derivatives.

To explore the potential to manipulate the spin system in our TADF‐TEMPO designs, we use pulsed ESR (Figure S22–S24, Supporting Information). Cz‐TEMPO gives access to a long‐lived quartet, ^4^D_0_T_1_^(LE)^, and transient nutation reveals its spectrum does not carry signals of uncoupled triplets. The absence of triplet features in the nutation experiments confirms that the detected delayed emission originates from a quartet state. Quartet spin coherence time of Cz‐TEMPO is *T*_m_ = 0.8 ± 0.1 µs at 80 K in a fully protonated environment, comparable to that of non‐luminescent PDI‐TEMPO,^[^ ^10^ ^]^ and luminescent TTM‐1Cz‐An.^[^ ^11^ ^]^ We have also performed microwave manipulation experiments on the more luminescent DMAC‐TEMPO. The coherence time of its quartet state is lower than that of Cz‐TEMPO, but can be recovered via a dynamic decoupling pulse sequence (Figure S30, Supporting Information). As the orbital composition of these quartet wavefunctions are similar, it can also be placed in an arbitrary superposition (Figure S31, Supporting Information).

To clarify the energetic landscape of our compounds, we use density functional theory to model the vertical and adiabatic transitions of the closed‐shell derivatives (Section S7, Supporting Information). The adiabatic state diagrams of Cz‐Cy and DMAC‐Cy are similar, with ^3^LE_NAI_ remaining the lowest‐lying excited state along the curve (Figure S25, Supporting Information). The ^1^CT and ^3^CT states of both compounds are almost degenerate in their respective excited state geometries (Tables S3–S5, Supporting Information). In contrast, PXZ‐Cy has all three excited states almost iso‐energetic at the same geometries, with ^3^LE_NAI_ above the CT states at the ^1^CT minimum. This reveals that the energetic ordering of the triplet states depends on molecular conformation for PXZ‐Cy, which is consistent with the observation of both triplet states contributing to the trESR signals for the PXZ compounds.

## 3 Discussion

**Figure 4:**
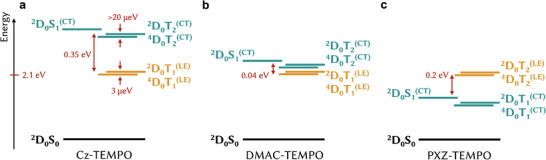
Energetics of TEMPO‐derivatives. All three donor units have two triplet states near the emissive singlet, with the position of the CT triplet shifting with the choice of donor and the position of the LE triplet pinned near 2.1 eV (localized on the NAI core). Both triplets split into a pair of trip‐doublet and trip‐quartet levels, with LE‐born species separated by 3 µeV due to intermediate regime of exchange coupling and CT‐born species separated by at least 20 µeV due to strong regime of exchange coupling. a) Cz‐TEMPO has a high yield of the ^4^D_0_T_1_^(LE)^ state but thereafter cannot efficiently emit light from ^2^D_0_S_1_ due to the substantial ≈0.35 eV energy gap. b) DMAC‐TEMPO is the best TADF‐TEMPO structure, as due to thermally accessible energy gaps, RISC from ^4^D_0_T_1_^(LE)^ toward ^2^D_0_S_1_ occurs with an activation of ≈0.04 eV. c) In PXZ‐TEMPO the CT states lie below the LE triplet, leading to both ^4^D_0_T_1_^(CT)^ and ^4^D_0_T_2_^(LE)^ being populated, likely in different molecular conformations. The values of the energies are estimated from experimental results corresponding to the dominant conformations.

We are now able to construct state diagrams for the three TEMPO‐derivatives (**Figure** **4**), based particularly on insights from luminescence spectroscopy and trESR simulations. As radical‐triplet exchange is in the intermediate regime for all compounds, the Jabłoński diagrams can be found from matching Cy‐compounds, but with each triplet state T_n_ split into a pair of closely spaced ^2^D_0_T_n_ and ^4^D_0_T_n_ levels.

Light absorption in the visible region occurs predominantly along the ^2^D_0_S_0_→^2^D_0_S_1_^(CT)^ transition on the TADF, due to the low extinction coefficient of TEMPO (Figure S12, Supporting Information). The band gap is determined by the choice of donor unit. Since the position of the ^2,4^D_0_T_n_^(LE)^ states is the same for all TEMPO‐derivatives, the energetic ordering of the LE‐ and CT‐character states is varied through the series. In Cz‐TEMPO and DMAC‐TEMPO, the lowest energy triplet has LE character, but in PXZ‐TEMPO the ^2,4^D_0_T_2_^(LE)^ lie above the CT‐character states, leading to both quartets being populated.

DMAC‐TEMPO is the prototypical TADF‐TEMPO compound, as the energy gap between the ^2,4^D_0_T_n_^(LE)^ and ^2^D_0_S_1_^(CT)^ is of the order of Boltzmann energy at room temperature. This is confirmed by the dominance of delayed emission in films. The small fraction of ^4^D_0_T_2_^(CT)^ features in the delayed component of trESR indicates that the RISC toward the emissive state is efficient, and does not lead to pooling of excitons in the other quartet state.

For all TEMPO‐derivatives, ^2^D_0_S_1_^(CT)^ is the only state from which luminescence is detected at room temperature. However, not all observed emission passes through a quartet state. The energy gap between the emissive and the ^4^D_0_T_1_^(LE)^ states in Cz‐TEMPO leads to the majority of emission occurring before the quartet is formed. In contrast, the well‐matched energetics of DMAC‐TEMPO allow delayed emission due to RISC from the lowest quartet state to be the dominant luminescence channel.

Our Radical TADF design already achieves an ≈200 nm luminescence wavelength range, with potential for further expansion. The coherence times need to be improved in future designs to enable room‐temperature spin manipulations. As organic materials are free from heavy atoms, they can be protected them from spin‐orbit‐coupling‐ and hyperfine‐mediated decoherence pathways.^[^ ^37^ ^]^ This contributes to their high quantum fidelities at low temperatures compared to alternative platforms (Section S6, Supporting Information).

Operating on excited states rather than ground states offers a facile way to increase spin multiplicity by engineering ferromagnetic exchange coupling between neighboring chemical units. Quantum logical operations on such multilevel states can be performed with significant performance advantages in fields ranging from quantum simulation to error correction.^[^ ^38^, ^39^ ^]^ However, in designs where the excited state is manipulated electron state coherence can be limited by the photophysical processes leading to luminescence. This first generation of Radical TADF offers sufficient T_m_ times at liquid nitrogen temperatures, with scope to improvement through engineering of local hyperfine and conformational environment.

## 4 Conclusion

This study presents a new design strategy to achieve an emission pathway from a quartet state which does not rely on the presence of a luminescent π‐radical. Due to the of both synthetically accessible non‐luminescent radicals and TADF chromophores, compared to the few efficient luminescent radicals, it promises a much greater range of possible structures bearing both electron‐rich and electron‐deficient functionalities. More broadly, by demonstrating how luminescence can be switched‐on in TEMPO‐based molecules, it showcases the so far unexplored potential of σ‐radicals in optoelectronic research.

While the PLQE can be boosted by engineering the TADF chromophore, improving yield of forward and reverse spin flip processes, and minimizing non‐radiative losses, the key for optical read‐out is maximizing not total emission output, but the fraction of RISC from the quartet to the emissive state. In DMAC‐TEMPO this is already at a substantial 72% of total emission. The PLQE achieved here is of the same order of magnitude to those in analogous established platforms, such as nitrogen‐vacancy (NV) centers in nanodiamonds.^[^ ^40^ ^]^

The intermediate exchange regime for the triplet‐radical interaction in DMAC‐TEMPO may offer additional benefits in applications. As the microwave resonance conditions are very sensitive to the *D*/*J_TR_* ratio in this regime, small perturbations from local magnetic fields may be detectable more easily than in the strong coupling regime in a future sensing demonstration.

The TADF‐TEMPO design presented here offers a viable pathway to quartet‐derived luminescence spanning the entire visible range. More complex molecular structures may then designed with multi‐wavelength read‐out which could offer new possibilities for interfacing.

## 5 Experimental Section

### Transient Absorption Spectroscopy

Transient absorption experiments were conducted on a setup pumped by a regenerative amplifier (Light Conversion, Pharos) emitting sub‐200 fs pulses centered at 1035 nm at a rate of 10 kHz. The output of the amplifier was optically delayed up to 8 ns by a multi‐pass computer‐controlled delay stage. This was used to generate a 500–1000 nm white light (WL) in a sapphire crystal. For generating a 350–500 nm WL, the seed was doubled in a BBO crystal before the sapphire. Wavelength‐tunable pump pulses were generated in an Orpheus Neo (Light Conversion) unit. The pump was chopped at 100 Hz to provide on average a 5 kHz repetition rate for pump‐on and pump‐off measurements. The pump spectrum was filtered using appropriate band‐pass filters to remove residual wavelengths, and its polarization set to magic angle relative to the probe pulses using a Berek rotator. The pump and probe beams were spatially overlapped at the focal point using a beam profiler. The pump and probe diameter at the sample position were on the order of 1000 and 100 µm respectively. After passing through the sample, the probe beam was dispersed with a grating spectrometer (Kymera, Andor Technology) and measured with a Si detector array.

### Transient Photoluminescence Spectroscopy

Time‐resolved PL spectra were collected using an electrically‐gated intensified CCD (ICCD) camera (AndoriStar DH740 CCI‐010) coupled with an image identifier tube after passing through a calibrated grating spectrometer (Andor SR303i). The spectrometer input slit width was 200 µm. The samples were excited using pump pulses obtained from Orpheus‐Lyra (Light Conversion) driven by the same amplifier as the TA setups. The pump repetition rate was reduced to 1 kHz. A suitable long‐pass filter was placed directly in front of the spectrometer to avoid the scattered laser signals entering the camera. The kinetics of PL emissions can be obtained by setting the gate delay steps with respect to the excitation pulse. Overlapping time regions were used to compose the decays at several constant gate widths (typically 5 ns, 50 ns, 500 ns, 5 µs, and 50 µs). Temperature‐dependent measurements were performed using a closed‐circuit pressurized helium cryostat (Optistat Dry BL4, Oxford Instruments), compressor (HC‐4E2, Sumitomo), and temperature controller (Mercury iTC, Oxford Instruments).

### Electron Spin Resonance

X‐band ESR was acquired with a Bruker Biospin E680 or E580 EleXSys spectrometer using a Bruker ER4118‐MD5‐W1 dielectric TE01_δ_ mode resonator (≈9.70 GHz) in an Oxford Instruments CF935 cryostat. Q‐band ESR employed an EN5107QD2 resonator and a conventional 1.5 T electromagnet like X‐band frequencies. The amplifiers for pulsed ESR, Applied Systems Engineering (ASE), had saturated powers of 1.5 kW at X‐band and 180 W at Q‐band. Temperature was maintained with an ITC‐503S temperature controller and a CF‐935 helium flow cryostat (both Oxford Instruments). Temperature control was achieved with liquid nitrogen or helium flow and an Oxford Instruments ITC‐503s temperature controller. For laser‐induced transient signals, photoexcitation was provided by either Ekspla NT230 operating at a repetition rate of 50 Hz or Opotek Opolette HE355 operating at 20 Hz. Laser pulse energies used were 0.5–1 mJ, pulse lengths of 3 ns transmitted at ca. 40% to the sample via the cryostat, microwave shield, and resonator windows. A liquid‐crystal depolarizer (DPP‐25, ThorLabs) was placed in the laser path for all measurements unless indicated. Triggering of the LASER and ESR spectrometer involved synchronization with a Stanford Research Systems delay generator, DG645. Quadrature mixer detection was used in pulsed and continuous wave detection. Transient cw ESR spectra were simulated using EasySpin.^[^ ^41^, ^42^ ^]^

### Quantum Chemical Calculations

All DFT calculations were performed using the ORCA software package.^[^ ^43^ ^]^ Geometry optimization of the ground state was performed with CAM‐B3LYP functional and the def2‐TZVP basis set. Vertical excitations and excited‐state geometry optimizations were performed with time‐dependent DFT within the Tamm‐Dancoff approximation, utilizing the root following scheme at the same level of theory as the ground state optimizations. The absence of imaginary frequencies in the calculated Hessians confirmed the stationary nature of converged geometries. Solvent effects were included in all calculations using the polarizable continuum model (PCM) as implemented in Orca, with an *𝜀* of 2.38, corresponding to toluene. Subsequent wavefunction analysis was performed with the Multiwfn package,^[^ ^44^, ^45^ ^]^ and a combination of homemade scripts.

CCDC 2388206, 2388207, 2388209, 2388205, and 2388208 contain the supplementary crystallographic data for this paper. These data can be obtained free of charge via www.ccdc.cam.ac.uk/data_request/cif, or by emailing data_request@ccdc.cam.ac.uk, or by contacting The Cambridge Crystallographic Data Centre, 12 Union Road, Cambridge CB2 1EZ, United Kingdom; fax: +44 1223 336033.

## Conflict of Interest

The authors declare no conflict of interest.

## Author Contributions

S.G. conceived the project. S.G., D.G.C., and P.M. performed the photophysical measurements. S.G. and W.K.M. performed the ESR measurements. P.M. and D.G.C. synthesized the compounds and performed the chemical characterization. L.M. performed the quantum chemical modelling. A.D.B. carried out the X‐ray crystallography and data analysis. V.R.‐G. carried out the thermal stability measurements. S.G. wrote the manuscript with input from all authors. H.B. and R.H.F. provided resources.

## Supporting information



Supporting Information

## Data Availability

Data are available from the University of Cambridge Apollo Repository at https://doi.org/10.17863/CAM.118024.
