# Supplementary material for: Radical TADF: Quartet‐Derived Luminescence with Dark TEMPO
Source: Adv Mater. 2025 May 15;37(30):2501164. doi: 10.1002/adma.202501164 (PMC12306414; doi:10.1002/adma.202501164)
Supplement: Supplementary file 1 — Supporting Information [file ADMA-37-2501164-s001.docx]

Supporting Information

Radical TADF: Quartet-derived luminescence with dark TEMPO

Sebastian Gorgon^*^, Petri Murto, Daniel G. Congrave, Lujo Matasovic, Andrew D. Bond, Victor Riesgo-Gonzalez, William K. Myers, Hugo Bronstein, Richard H. Friend

Contents:

[1. Synthesis and characterisation 2](#_Toc194413779)

[2. X-Ray crystallography 15](#_Toc194413780)

[3. Cyclic voltammetry 25](#_Toc194413781)

[4. Photophysics 27](#_Toc194413782)

[5. Electron Spin Resonance 31](#_Toc194413783)

[6. System comparison 36](#_Toc194413784)

[7. Quantum chemical calculations 37](#_Toc194413785)

[8. NMR spectra 43](#_Toc194413786)

[9. SI References 53](#_Toc194413787)

# Synthesis and characterisation


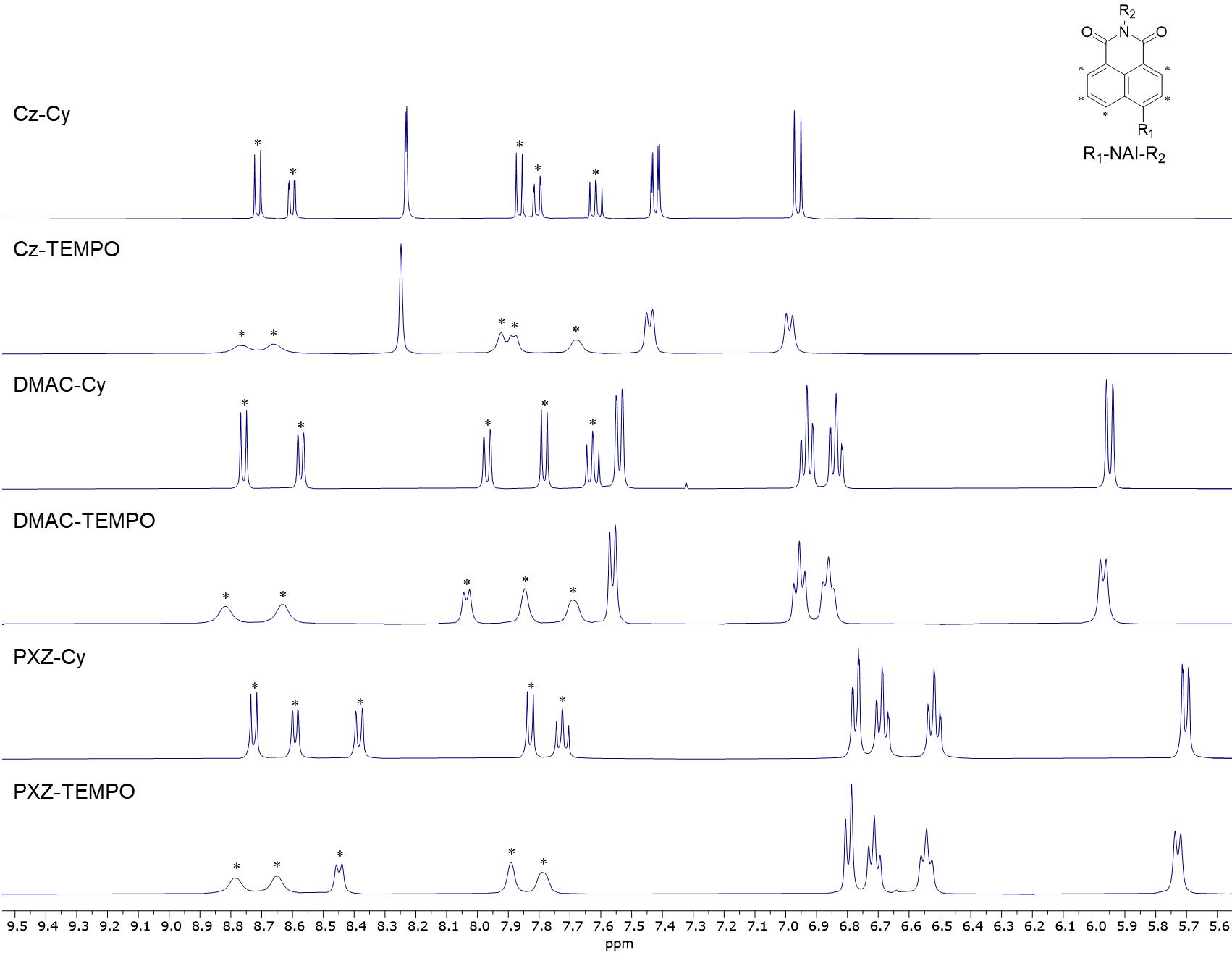


**Fig. S1** ^1^H NMR spectra (CD_2_Cl_2_) of the synthesized TADF and TADF-TEMPO molecules showing broadening of the aromatic signals of the NAI unit (black asterisks) in the radical containing derivatives (see Section 8 for full analysis).


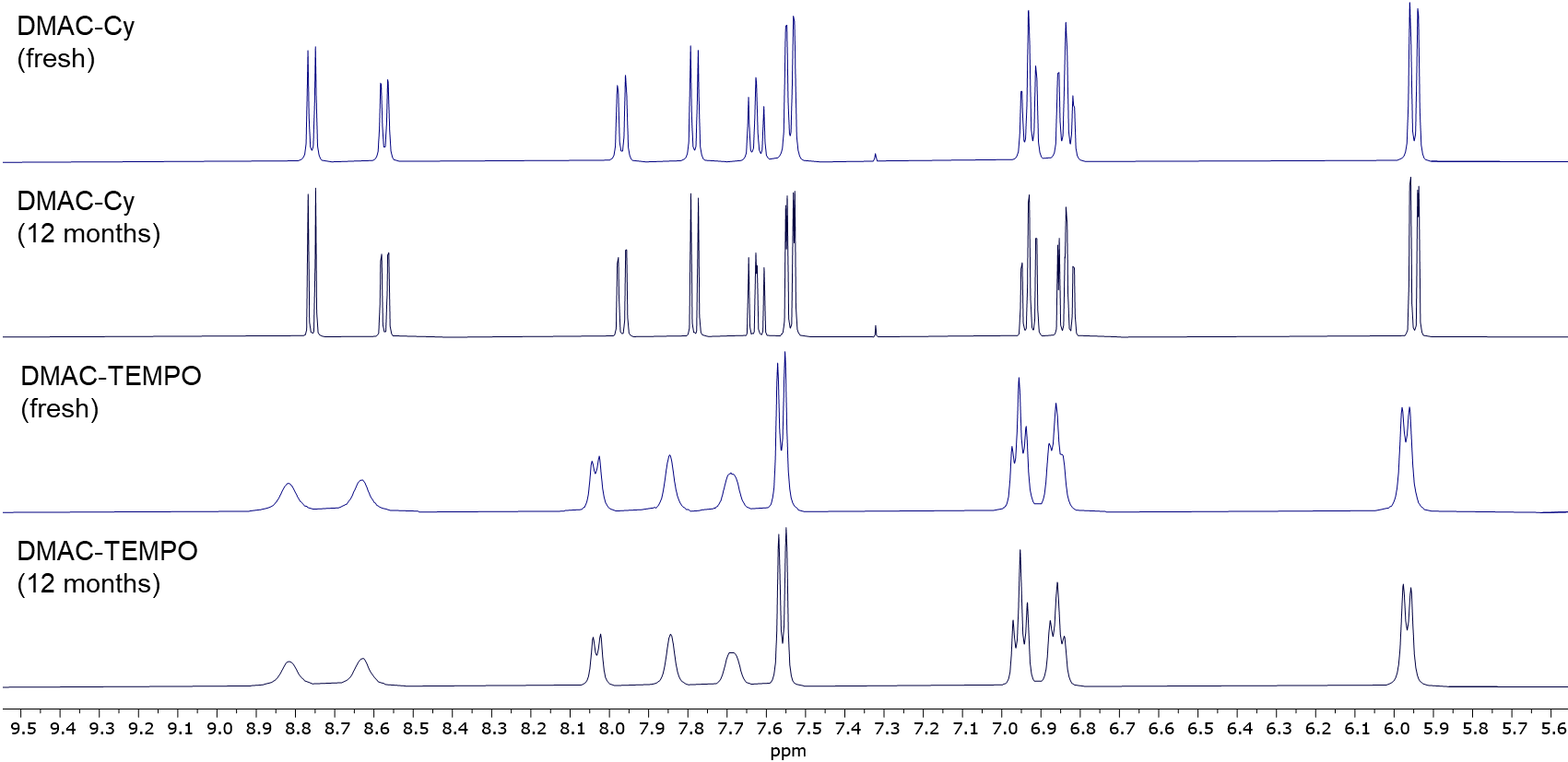


**Fig. S2** Stability of ^1^H NMR spectra (CD_2_Cl_2_) of DMAC-Cy and DMAC-TEMPO. The powder samples were stored in air at ambient conditions in the dark for just over one calendar year. No chemical changes occurred, including no loss of the radical unit, as evidenced by the same paramagnetic shifts and line broadenings as in the fresh sample (cf. Fig. S1).

**Fig. S3** Synthesis of TADF molecules.

**Fig. S4** Synthesis of TADF-TEMPO molecules.

**Characterization and techniques.** NMR spectra were recorded on a 400 MHz Bruker Avance III HD spectrometer (^1^H, 400 MHz; ^13^C, 100 MHz). Chemical shifts are reported in *δ* (ppm) relative to the solvent peak: dichloromethane-*d*_2_ (CD_2_Cl_2_: ^1^H, 5.32 ppm; ^13^C, 53.84 ppm) and chloroform-*d* (CDCl_3_: ^1^H, 7.26 ppm; ^13^C, 77.16 ppm). High resolution mass spectra were obtained using electrospray ionization (ESI) and atmospheric solids analysis probe (ASAP) techniques by the Mass Spectrometry service at Yusuf Hamied Department of Chemistry, University of Cambridge. Flash chromatography was carried out using Biotage^®^ Isolera™ Four System and Biotage^®^ SNAP/Sfär Silica flash cartridges.

**Materials and synthesis.** The 1,8-naphthalic anhydride, 4-bromo-1,8-naphthalic anhydride, 4-amino-TEMPO radical, cyclohexylamine, 3,6-di-*tert*-butylcarbazole, 9,9-dimethyl-9,10-dihydroacridine and phenoxazine monomers and other reagents, catalysts and solvents were purchased from Merck, Fluorochem, Alfa Aesar and Acros Organics and used as received.

*Note: While the syntheses reported here for* ***Cy-NAI*** *and* ***Cy-NAI-Br*** *are rather low yielding, they were sufficient for our needs, given the cheap commercial availability of the starting materials and the facile isolation of high purity crystalline product direct from the reaction mixture. We expect higher yields could be obtained upon further optimisation of the purification procedure.*

**2-Cyclohexyl-1*H*-benzo[*de*]isoquinoline-1,3(2*H*)-dione (Cy-NAI).**^[1]^ To a flask equipped with a condenser was added 1,8-naphthalic anhydride (2.77 g, 14.0 mmol, 1 equiv.), cyclohexylamine (1.72 g, 17.0 mmol, 1.2 equiv.) and ethanol (110 mL). The mixture was refluxed overnight and then cooled to room temperature and left to sit for 24 h. The precipitated crystals were collected by filtration, washed with ethanol (3 × 20 mL) and dried under suction to obtain the target compound as colourless needles (0.75 g, 19%). ^1^H NMR (400 MHz, CD_2_Cl_2_) δ 8.53 (dd, *J* = 7.3, 1.2 Hz, 2H), 8.21 (dd, *J* = 8.4, 1.0 Hz, 2H), 7.74 (dd, *J* = 8.3, 7.2 Hz, 2H), 5.00 (tt, *J* = 12.1, 3.7 Hz, 1H), 2.53 (qd, *J* = 12.2, 3.4 Hz, 2H), 1.93–1.84 (m, 2H), 1.76–1.68 (m, 3H), 1.52–1.39 (m, 2H), 1.39–1.28 (m, 1H). FTMS-ESI^+^ Calcd. for [C_18_H_17_NO_2_]^H+^: 280.1332. Found: *m/z* = 280.1331.

**6-Bromo-2-cyclohexyl-1*H*-benzo[*de*]isoquinoline-1,3(2*H*)-dione (Cy-NAI-Br).**^[2]^ To a flask equipped with a condenser was added 4-bromo-1,8-naphthalic anhydride (4.00 g, 14.0 mmol, 1 equiv.), cyclohexylamine (1.72 g, 17.0 mmol, 1.2 equiv.) and ethanol (110 mL). The mixture was refluxed overnight and then cooled to room temperature and left to sit for 24 h. The precipitated crystals were collected by filtration, washed with ethanol (3 × 20 mL) and dried under suction to obtain the target compound as colourless needles (1.93 g, 39%). ^1^H NMR (400 MHz, CDCl_3_) δ 8.62 (dd, *J* = 7.2, 1.1 Hz, 1H), 8.54 (dd, *J* = 8.5, 1.1 Hz, 1H), 8.38 (d, *J* = 7.8 Hz, 1H), 8.02 (d, *J* = 7.8 Hz, 1H), 7.83 (dd, *J* = 8.5, 7.3 Hz, 1H), 5.00 (tt, *J* = 12.2, 3.9 Hz, 1H), 2.53 (qd, *J* = 12.5, 3.4 Hz, 2H), 1.94–1.85 (m, 2H), 1.77–1.68 (m, 3H), 1.52–1.38 (m, 2H), 1.38–1.28 (m, 1H). FTMS-ESI^+^ Calcd. for [C_18_H_16_BrNO_2_]^H+^: 358.0437. Found: *m/z* = 358.0428.

**2-Cyclohexyl-6-(3,6-di-*tert*-butyl-9*H*-carbazol-9-yl)-1*H*-benzo[*de*]isoquinoline-1,3(2*H*)-dione (Cz-Cy).** Adapted from a literature procedure,^[3]^ to an oven-dried crimp cap vial was added 3,6-di-*tert*-butylcarbazole (0.108 g, 0.39 mmol, 1.2 equiv.) and the vial was subjected to three vacuum/Ar gas refill cycles. Anhydrous THF (0.5 mL) and toluene (1 mL) were added, and the mixture was bubbled with Ar gas for 5 min, and then cooled to 5 °C in an ice bath. 1 M solution of *t*-BuMgCl in THF (0.50 mL, 0.50 mmol, 1.3 equiv.) was added dropwise. After the addition was complete, the mixture was allowed to warm to room temperature. To a separate oven-dried crimp cap vial was added Cy-NAI-Br (0.115 g, 0.32 mmol, 1 equiv.), [Pd(allyl)Cl]_2_ (0.0012 g, 0.003 mmol, 0.01 equiv.) and *t*-BuXPhos (0.0055 g, 0.013 mmol, 0.04 equiv.), and the vial was subjected to three vacuum/Ar gas refill cycles. Anhydrous THF (1 mL) and toluene (1 mL) were added, and the mixture was bubbled with Ar gas for 5 min, and then cooled to 5 °C in an ice bath. To this second vial was transferred the carbazole-Grignard reaction mixture from the first vial dropwise, using a cannula. After the transfer was complete, the reaction mixture was stirred at 85 °C for 4 h. After cooling to room temperature, the mixture was diluted with DCM and extracted with water. The organic phase was dried over anhydrous MgSO_4_. Solvent was removed and the crude product was purified with column chromatography over silica gel by gradually increasing the eluent polarity from hexane to 10 vol% DCM in hexane. Finally, recrystallisation from MeOH/DCM gave the target compound, after drying *in vacuo*, as yellow solid (0.064 g, 32%). ^1^H NMR (400 MHz, CD_2_Cl_2_) δ 8.71 (d, *J* = 7.8 Hz, 1H), 8.60 (dd, *J* = 7.3, 1.2 Hz, 1H), 8.23 (d, *J* = 1.5 Hz, 2H), 7.86 (d, *J* = 7.8 Hz, 1H), 7.81 (dd, *J* = 8.4, 1.2 Hz, 1H), 7.62 (dd, *J* = 8.5, 7.3 Hz, 1H), 7.42 (dd, *J* = 8.7, 2.0 Hz, 2H), 6.96 (d, *J* = 8.5 Hz, 2H), 5.05 (tt, *J* = 12.2, 3.8 Hz, 1H), 2.58 (qd, *J* = 12.1, 3.3 Hz, 2H), 1.96–1.88 (m, 2H), 1.84–1.70 (m, 3H), 1.50–1.39 (m, 2H), 1.46 (s, 18H), 1.39–1.28 (m, 1H). ^13^C NMR (100 MHz, CD_2_Cl_2_) δ 164.23, 163.85, 143.63, 140.26, 140.21, 131.40, 131.20, 129.73, 129.67, 128.75, 127.30, 126.94, 124.06, 123.93, 123.68, 123.04, 116.47, 109.40, 34.65, 31.66, 29.10, 26.58, 25.53. FTMS-ESI^+^ Calcd. for [C_38_H_40_N_2_O_2_]^H+^: 557.3163. Found: *m/z* = 557.3154.

**2-Cyclohexyl-6-(9,9-dimethylacridin-10(9*H*)-yl)-1*H*-benzo[*de*]isoquinoline-1,3(2*H*)-dione (DMAC-Cy).** Adapted from a literature procedure,^[4]^ to an oven-dried crimp cap vial was added Cy-NAI-Br (0.100 g, 0.28 mmol, 1 equiv.), 9,9-dimethyl-9,10-dihydroacridine (0.088 g, 0.42 mmol, 1.5 equiv.), Pd(OAc)_2_ (0.0006 g, 0.003 mmol, 0.01 equiv.), (*t*-Bu)_3_PHBF_4_ (0.0024 g, 0.008 mmol, 0.03 equiv.) and sodium *tert*-amoxide (0.043 g, 0.39 mmol, 1.4 equiv.), and the vial was subjected to three vacuum/Ar gas refill cycles. Anhydrous toluene (4 mL) was added, and the mixture was bubbled with Ar gas for 15 min, and then stirred at 120 °C in an oil bath for 20 h. After cooling to room temperature, the mixture was diluted with DCM and extracted with water. The organic phase was dried over anhydrous MgSO_4_. Solvent was removed and the crude product was purified with column chromatography over silica gel by gradually increasing the eluent polarity from hexane to 40 vol% DCM in hexane. Recrystallisation from MeOH/DCM gave the target compound, after drying *in vacuo*, as orange solid (0.120 g, 88%). ^1^H NMR (400 MHz, CD_2_Cl_2_) δ 8.76 (d, *J* = 7.6 Hz, 1H), 8.57 (dd, *J* = 7.3, 1.2 Hz, 1H), 7.97 (dd, *J* = 8.4, 1.2 Hz, 1H), 7.78 (d, *J* = 7.7 Hz, 1H), 7.63 (dd, *J* = 8.5, 7.2 Hz, 1H), 7.54 (dd, *J* = 7.8, 1.7 Hz, 2H), 6.97–6.89 (m, 2H), 6.88–6.80 (m, 2H), 5.95 (dd, *J* = 8.2, 1.3 Hz, 2H), 5.05 (tt, *J* = 12.2, 3.9 Hz, 1H), 2.58 (qd, *J* = 12.3, 3.5 Hz, 2H), 1.96–1.88 (m, 2H), 1.85–1.72 (m, 9H), 1.52–1.29 (m, 3H). ^13^C NMR (100 MHz, CD_2_Cl_2_) δ 164.56, 164.26, 143.93, 140.56, 132.58, 131.85, 131.12, 130.82, 130.74, 130.36, 129.83, 128.28, 127.00, 126.38, 124.88, 124.14, 121.51, 114.34, 36.39, 32.92, 32.42, 29.53, 27.02, 25.97. FTMS-ESI^+^ Calcd. for [C_33_H_30_N_2_O_2_]^+^: 486.2302. Found: *m/z* = 486.2293.

**2-Cyclohexyl-6-(10*H*-phenoxazin-10-yl)-1*H*-benzo[*de*]isoquinoline-1,3(2*H*)-dione (PXZ-Cy).** Following the procedure described above for DMAC-Cy, Cy-NAI-Br (0.200 g, 0.56 mmol, 1 equiv.), phenoxazine (0.153 g, 0.84 mmol, 1.5 equiv.), Pd(OAc)_2_ (0.0013 g, 0.006 mmol, 0.01 equiv.), (*t*-Bu)_3_PHBF_4_ (0.0049 g, 0.017 mmol, 0.03 equiv.), sodium *tert*-amoxide (0.086 g, 0.78 mmol, 1.4 equiv.) and anhydrous toluene (8 mL) were used. Column chromatography was carried out by gradually increasing the eluent polarity from hexane to 20 vol% DCM in hexane. Recrystallisation from MeOH/DCM gave the target compound, after drying *in vacuo*, as red solid (0.205 g, 80%). ^1^H NMR (400 MHz, CD_2_Cl_2_) δ 8.73 (d, *J* = 7.7 Hz, 1H), 8.59 (dd, *J* = 7.3, 1.3 Hz, 1H), 8.38 (dd, *J* = 8.5, 1.3 Hz, 1H), 7.83 (d, *J* = 7.7 Hz, 1H), 7.77–7.68 (m, 1H), 6.81–6.73 (m, 2H), 6.69 (td, *J* = 7.6, 1.5 Hz, 2H), 6.52 (td, *J* = 7.8, 1.6 Hz, 2H), 5.70 (dd, *J* = 8.0, 1.5 Hz, 2H), 5.03 (tt, *J* = 12.2, 3.7 Hz, 1H), 2.56 (qd, *J* = 12.3, 3.4 Hz, 2H), 1.96–1.86 (m, 2H), 1.83–1.68 (m, 3H), 1.53–1.42 (m, 2H), 1.40–1.30 (m, 1H). ^13^C NMR (100 MHz, CD_2_Cl_2_) δ 164.46, 164.13, 144.22, 141.28, 134.18, 132.49, 131.96, 130.83, 130.75, 130.45, 129.45, 128.43, 124.89, 124.35, 123.87, 122.43, 116.08, 113.78, 29.51, 27.00, 25.94. FTMS-ESI^+^ Calcd. for [C_30_H_24_N_2_O_3_]^+^: 460.1781. Found: *m/z* = 460.1777.

**2-TEMPO-1*H*-benzo[*de*]isoquinoline-1,3(2*H*)-dione radical (TEMPO-NAI).** Adapted from literature procedures,^[5,6]^ to an oven-dried flask equipped with a condenser was added 1,8-naphthalic anhydride (0.622 g, 3.14 mmol, 1 equiv.) and zinc acetate (0.137 g, 0.63 mmol, 0.2 equiv.), and the flask was subjected to three vacuum/Ar gas refill cycles. 4-Amino-TEMPO radical (2.15 g, 12.6 mmol, 4 equiv.) was dissolved in anhydrous pyridine (10 mL) and added to the flask. The mixture was bubbled with Ar gas for 15 min and then stirred at 110 °C in an oil bath for 20 h until all naphthalic anhydride had reacted. After cooling to room temperature, the mixture was poured into 1 M HCl (200 mL). The precipitated was collected by filtration and washed with methanol (3 × 10 mL). The crude product was purified with column chromatography over silica gel by gradually increasing the eluent polarity from hexane to 100 vol% DCM. Finally, the solvent was removed, and the solids were dried *in vacuo*. The target compound was collected as orange solid (1.00 g, 74%). ^1^H NMR (400 MHz, CD_2_Cl_2_) δ 8.59 (d, *J* = 6.6 Hz, 2H), 8.27 (d, *J* = 7.8 Hz, 2H), 7.81 (br, 2H). Broad aromatic signals (NAI, 2H). Aliphatic signals not resolved (TEMPO, 17H). FTMS-ESI^+^ Calcd. for [C_21_H_23_N_2_O_3_]^+^: 351.1703. Found: *m/z* = 351.1700.

**6-Bromo-2-TEMPO-1*H*-benzo[*de*]isoquinoline-1,3(2*H*)-dione radical (TEMPO-NAI-Br).** Adapted from literature procedures,^[5,6]^ to an oven-dried flask equipped with a condenser was added 4-bromo-1,8-naphthalic anhydride (1.00 g, 3.6 mmol, 1 equiv.) and zinc acetate (0.158 g, 0.72 mmol, 0.2 equiv.), and the flask was subjected to three vacuum/Ar gas refill cycles. 4-Amino-TEMPO radical (2.47 g, 14.4 mmol, 4 equiv.) was dissolved in anhydrous pyridine and added to the flask, followed by addition of anhydrous pyridine (total 70 mL). The mixture was bubbled with Ar gas for 15 min and then stirred at 110 °C in an oil bath for 20 h until all naphthalic anhydride had reacted. After cooling to room temperature, the mixture was diluted with chloroform and extracted with water. The aqueous phase was acidified with 1 M HCl solution, and the separated organic phase was extracted again with water. The organic phase was dried over anhydrous MgSO_4_. Solvent was removed and the crude product was purified with column chromatography over silica gel by gradually increasing the eluent polarity from hexane to 49 vol% DCM and 2 vol% MeOH in hexane. Finally, the solvent was removed, and the solids were dried *in vacuo*. The target compound was collected as orange solid (1.44 g, 92%). ^1^H NMR (400 MHz, CD_2_Cl_2_) δ 8.66 (br, 1H), 8.60 (d, *J* = 7.8 Hz, 1H), 8.41 (br, 1H), 8.09 (br, 1H), 7.91 (br, 1H). Broad aromatic signals (NAI, 4H). Aliphatic signals not resolved (TEMPO, 17H). TOF-MS-ASAP^+^ Calcd. for [C_21_H_22_N_2_O_3_Br]^+^: 429.0814. Found: *m/z* = 429.0824.

**2-TEMPO-6-(3,6-di-*tert*-butyl-9*H*-carbazol-9-yl)-1*H*-benzo[*de*]isoquinoline-1,3(2*H*)-dione radical (Cz-TEMPO).** Following the procedure described above for Cz-Cy, 3,6-di-*tert*-butylcarbazole (0.078 g, 0.28 mmol, 1.2 equiv.), anhydrous THF (0.5 mL) and toluene (1 mL) and 1 M solution of *t*-BuMgCl in THF (0.36 mL, 0.36 mmol, 1.3 equiv.) were used to make the carbazole-Grignard reaction mixture, followed by TEMPO-NAI-Br (0.100 g, 0.23 mmol, 1 equiv.), [Pd(allyl)Cl]_2_ (0.0009 g, 0.002 mmol, 0.01 equiv.), *t*-BuXPhos (0.0039 g, 0.009 mmol, 0.04 equiv.) and anhydrous THF (1 mL) and toluene (1 mL). Column chromatography was carried out by gradually increasing the eluent polarity from hexane to 4 vol% MeOH in DCM. Recrystallisation from MeOH/DCM gave the target compound, after drying *in vacuo*, as yellow solid (0.030 g, 21%). ^1^H NMR (400 MHz, CD_2_Cl_2_) δ 8.77 (br, 1H), 8.66 (br, 1H), 8.25 (s, 2H), 7.92 (br, 1H), 7.88 (d, *J* = 6.7 Hz, 1H), 7.68 (br, 1H), 7.44 (d, *J* = 7.5 Hz, 2H), 6.99 (d, *J* = 8.4 Hz, 2H), 1.48 (s, 18H). Broad aromatic signals (NAI, 4H). Remaining aliphatic signals not resolved (TEMPO, 17H). FTMS-ESI^+^ Calcd. for [C_41_H_46_N_3_O_3_]^H+^: 629.3612. Found: *m/z* = 629.3594.

**2-TEMPO-6-(9,9-dimethylacridin-10(9*H*)-yl)-1*H*-benzo[*de*]isoquinoline-1,3(2*H*)-dione radical (DMAC-TEMPO).** Following the procedure described above for DMAC-Cy, TEMPO-NAI-Br (0.200 g, 0.46 mmol, 1 equiv.), 9,9-dimethyl-9,10-dihydroacridine (0.146 g, 0.70 mmol, 1.5 equiv.), Pd(OAc)_2_ (0.0010 g, 0.005 mmol, 0.01 equiv.), (*t*-Bu)_3_PHBF_4_ (0.0040 g, 0.014 mmol, 0.03 equiv.), sodium *tert*-amoxide (0.072 g, 0.65 mmol, 1.4 equiv.) and anhydrous toluene (7 mL) were used. Column chromatography was carried out by gradually increasing the eluent polarity from hexane to 1 vol% MeOH in DCM. Finally, the solvent was removed, and the solids were dried *in vacuo*. The target compound was collected as orange solid (0.161 g, 62%). ^1^H NMR (400 MHz, CD_2_Cl_2_) δ 8.82 (br, 1H), 8.63 (br, 1H), 8.04 (d, *J* = 7.9 Hz, 1H), 7.85 (br, 1H), 7.69 (br, 1H), 7.56 (d, *J* = 7.6 Hz, 2H), 6.96 (t, *J* = 7.3 Hz, 2H), 6.86 (t, *J* = 6.9 Hz, 2H), 5.97 (d, *J* = 8.4 Hz, 2H), 1.85 (s, 3H), 1.78 (s, 3H). Broad aromatic signals (NAI, 4H). Remaining aliphatic signals not resolved (TEMPO, 17H). FTMS-ESI^+^ Calcd. for [C_36_H_36_N_3_O_3_]^+^: 558.2751. Found: *m/z* = 558.2743.

**2-TEMPO-6-(10*H*-phenoxazin-10-yl)-1*H*-benzo[*de*]isoquinoline-1,3(2*H*)-dione radical (PXZ-TEMPO).** Following the procedure described above for DMAC-Cy, TEMPO-NAI-Br (0.100 g, 0.23 mmol, 1 equiv.), phenoxazine (0.064 g, 0.35 mmol, 1.5 equiv.), Pd(OAc)_2_ (0.0005 g, 0.002 mmol, 0.01 equiv.), (*t*-Bu)_3_PHBF_4_ (0.0020 g, 0.007 mmol, 0.03 equiv.), sodium *tert*-amoxide (0.036 g, 0.33 mmol, 1.4 equiv.) and anhydrous toluene (5 mL) were used. Column chromatography was carried out by gradually increasing the eluent polarity from hexane to 3 vol% MeOH in DCM. Finally, recrystallisation from MeOH/DCM gave the target compound, after drying *in vacuo*, as red solid (0.083 g, 67%). ^1^H NMR (400 MHz, CD_2_Cl_2_) δ 8.78 (br, 1H), 8.65 (br, 1H), 8.45 (d, *J* = 7.6 Hz, 1H), 7.89 (br, 1H), 7.79 (br, 1H), 6.80 (d, *J* = 7.9 Hz, 2H), 6.71 (t, *J* = 7.5 Hz, 2H), 6.54 (t, *J* = 7.2 Hz, 2H), 5.73 (d, *J* = 7.3 Hz, 2H). Broad aromatic signals (NAI, 4H). Aliphatic signals not resolved (TEMPO, 17H). FTMS-ESI^+^ Calcd. for [C_33_H_30_N_3_O_4_]^+^: 532.2231. Found: *m/z* = 532.2224.

**Thermal Gravimetry Analysis (TGA)**

The measurements were performed using a TGA5500 system from TA Instruments, samples were prepared under air and measured while heating from 30 °C to 700 °C, at a rate of 10 °C min^-1^ under N_2_ flow.

| **Compound** | **T_D_ 95% (°C)** |
| --- | --- |
| **Cz-Cy** | 319 |
| **Cz-TEMPO** | 271 |
| **DMAC-Cy** | 269 |
| **DMAC-TEMPO** | 210 |
| **PXZ-Cy** | 312 |
| **PXZ-TEMPO** | 298 |

**Fig. S5** TGA data of all six compounds.

# X-Ray crystallography

Crystals were prepared by dissolving the sample in DCM in an NMR tube with MeOH added on top as an antisolvent and the solvents were allowed to mix slowly in the dark. Single-crystal X-ray diffraction data were collected on a Bruker D8-QUEST diffractometer, equipped with an Incoatec IμS Cu microsource (*λ* = 1.5418 Å) and a PHOTON-III detector operating in shutterless mode. The temperature was controlled at either 180(2) or 220(2) K using an Oxford Cryosystems open-flow N_2_ Cryostream. The control and processing software was Bruker *APEX5*. The diffraction images were integrated using *SAINT* in *APEX5* and a multi-scan correction was applied using *SADABS*. The final unit-cell parameters were refined against all reflections over the full data range. Structures were solved using *SHELXT* and refined using *SHELXL*.^[7]^ The crystal structures were visualized using the *Mercury* software.^[8]^ Crystal structures of DMAC-Cy, PXZ-Cy and PHX-TEMPO showed disorder related to an approximate 2-fold symmetry of the molecules. Structures are established for DMAC-Cy and PXZ-Cy, but the structure of PHX-TEMPO could not be confidently determined; see below for details.

**Details of X-ray crystallography**

**Cz-Cy.** The crystal structure clearly approximates space group *Pbcn* with *Z*' = 1, but refinement in that group stalled at *wR*2 ≈ 0.65 with unreasonably distorted displacement ellipsoids. The refinement in *Pnc2* with *Z*' = 2 gives a much better result.

**Cz-TEMPO.** The crystal contains DCM solvent molecules. These proved difficult to model as discrete molecules, so the solvent is omitted and *SQUEEZE* has been applied.^[9]^ *SQUEEZE* corrects for 156 electrons in the unit cell, contained in two symmetry-equivalent voids. In each void, 78 electrons corresponds to two DCM molecules (42 electrons per DCM). Hence, the unit cell contains 4 x DCM, and the best estimate of the empirical formula is Cz-TEMPO:DCM = 1:1.

**DMAC-TEMPO.** The crystals contain solvent. It is not clear from the Fourier map whether the molecules are DCM or MeOH (or both). Omitting the solvent and applying *SQUEEZE* gave a substantial improvement in the R-factors and precision of the structure. *SQUEEZE* corrects for 78 electrons per unit cell, contained in two symmetry-equivalent voids. In each void, 39 electrons corresponds approximately to one DCM molecule (42 electrons). It agrees less well with an EtOH molecule (26 electrons). A reasonable estimate of the empirical formula is therefore DMAC-TEMPO:DCM = 2:1.

**PXZ-Cy.** The best description identified for this crystal structure includes two orientations of the molecule overlaid, related to each other by a 2-fold rotation approximately aligned with the molecule’s long axis. Two whole sets of atoms were refined with the geometry controlled by a SAME restraint relating molecule 1 to molecule 2. A single parameter was refined for the site occupancies, converging to 0.609(3):0.391(3) for the two orientations. Anisotropic ADPs were applied to all non-H atoms, controlled through ISOR restraints. Introducing anisotropic ADPs reduced the R-factors substantially, and the resulting values indicate a good fit of the model to the X-ray data. Attempts to eliminate the disorder by reducing the symmetry to *P*1 or using a larger unit cell did not yield any obviously better result.

The two orientations of the molecule were extracted to yield two structures (each in space group P –1) that were subjected to periodic (plane-wave) DFT optimisation. Both structures converged to an energy minimum with minimal deviation from the refined X-ray coordinates. Overlay of the two DFT-optimised structures reproduces the X-ray refinement results very closely. The two structures show no significant difference in molecular conformation, but they are formally different crystal structures. It is not possible to conclude whether the crystals contain domains of the two structure types or whether the PHX-Cy molecule can simply adopt either orientation in each molecular site in the crystal.

**DMAC-Cy**. The structure contains disorder comparable to that seen in PHX-Cy. Two whole sets of atoms were refined with the geometry controlled by a SAME restraint relating molecule 1 to molecule 2. A single parameter was refined for the site occupancies, converging to 0.610(8):0.390(8) for the two orientations. In this case, all atoms were refined with isotropic displacement parameters. The major component supported anisotropic refinement with ISOR restraints, but the minor component yielded several non-positive definite atoms. Isotropic atoms were retained to simplify the model and to recognise the more limited data resolution and quality. Several crystals of DMAC-Cy were examined at different temperatures. Comparable results were obtained in all cases, but the reported result at 220(2) K is marginally better than other data sets.

The geometry of the overall structure resembles monoclinic. The 2-fold axes around which the molecules are disordered are perpendicular to the *ab* plane (*i.e.* parallel to *c**), and the structure can be transformed to space group *C*2/c with the approximate unit cell: *a* = 10.8, *b* = 23.5, *c* = 10.1 Å, α = 90, β = 105.5, γ = 90°. (transformation matrix: 0 -1 0 / 0 1 -2 / 1 0 0). The asymmetric unit then comprises one whole molecule, with the whole-molecule disorder imposed by the 2-fold rotation axes of the space group. Attempts to refine the structure in *C*2/*c* were significantly less successful than the reported refinement in *P*–1. The structure is refined as a 2-component twin, with the twin law corresponding to the 2-fold rotation parallel to *c**. This was implemented using *PLATON-TWINROTMAT* after data integration using the triclinic unit cell.^[10]^

The two orientations of the molecule were extracted to yield two structures (each in space group P –1) that were subjected to periodic (plane-wave) DFT optimisation. Both structures converge to an energy minimum with minimal deviation from the refined coordinates. Overlay of the two DFT-optimised structures reproduces the X-ray refinement results very closely, adding confidence that the whole-molecule disorder adequately describes the average situation in the crystal. The two optimised structures are identical, but in a different orientation. Applying *PLATON-ADDSYM* to either of the DFT-optimised structures does not suggest any higher-symmetry space group.

**PXZ-TEMPO (partial structure determination).** A crystal structure was determined for PXZ-TEMPO in space group *I*4_1_/*a*, with unit-cell parameters *a* = *b* ≈ 31.6, *c* ≈ 12.6 Å, α = β = γ = 90°, but the refinement stalled with *wR*2 ≈ 0.60. The structure showed whole-molecule disorder similar to that seen for DMAC-Cy and PXZ-Cy, and also substantial channels running along the crystallographic *c* axis containing diffuse solvent. Efforts to model the disorder, apply *SQUEEZE*, investigate other symmetry, *etc.*, ultimately did not yield a result that we could have sufficient confidence to include with this paper.

**Table S1 Experimental and crystal refinement parameters**

|  | **Cz-Cy** | **Cz-TEMPO**** | **DMAC-TEMPO**** | **DMAC-Cy** | **PXZ-Cy** |
| --- | --- | --- | --- | --- | --- |
| CCDC No. | 2388206 | 2388207 | 2388209 | 2388205 | 2388208 |
| Cambridge data number | HB_B1_0126 | HB_B1_0123 | HB_B1_0115 | HB_B1_0117 | HB_B1_0124 |
| Chemical formula | C_38_H_40_N_2_O_2_ | C_41_H_46_N_3_O_3_ | C_36_H_36_N_3_O_3_ | C_33_H_30_N_2_O_2_ | C_30_H_24_N_2_O_3_ |
| Formula weight | 556.72 | 628.81 | 558.68 | 486.59 | 460.51 |
| Temperature / K | 180(2) | 180(2) | 180(2) | 220(2) | 180(2) |
| Crystal system | orthorhombic | triclinic | monoclinic | triclinic | triclinic |
| Space group | P n c 2 | P –1 | P 2_1_/n | P –1 | P –1 |
| a / Å | 44.0323(11) | 13.2014(5) | 12.7968(4) | 10.1390(8) | 8.5292(3) |
| b / Å | 16.3858(5) | 14.5591(5) | 11.3020(4) | 10.8195(13) | 12.0386(4) |
| c / Å | 8.4979(2) | 20.8016(8) | 21.3810(7) | 12.9182(12) | 12.5514(4) |
| α / ° | 90 | 78.136(2) | 90 | 65.559(7) | 65.231(2) |
| β / ° | 90 | 75.400(2) | 96.927(2) | 83.716(6) | 74.281(2) |
| γ / ° | 90 | 84.976(2) | 90 | 74.591(7) | 81.402(2) |
| Unit-cell volume / Å^3^ | 6131.3(3) | 3783.6(2) | 3069.75(18) | 1243.7(2) | 1125.49(7) |
| Z, Z′ | 8, 2 | 4,2 | 4, 1 | 2, 1 | 2, 1 |
| Calc. density / g cm^–3^ | 1.206 | 1.104 | 1.209 | 1.299 | 1.359 |
| F(000) | 2384 | 1348 | 1188 | 516 | 484 |
| Radiation type | CuKα | CuKα | CuKα | CuKα | CuKα |
| Absorption coeff / mm^–1^ | 0.574 | 0.544 | 0.611 | 0.634 | 0.706 |
| Crystal size / mm^3^ | 0.20 x 0.14 x 0.06 | 0.14 x 0.14 x 0.04 | 0.18 x 0.10 x 0.04 | 0.18 x 0.10 x 0.02 | 0.22 x 0.10 x 0.06 |
| 2-Theta range / ° | 5.75-136.74 | 6.93-136.74 | 7.67-136.62 | 7.52-108.52 | 7.97-136.56 |
| Completeness to max 2θ | 0.997 | 0.993 | 0.998 | 0.999 | 0.990 |
| No. of refl. measured | 54476 | 60509 | 56715 | 3046 | 17217 |
| No. of independent refl. | 11198 | 13807 | 5631 | 3046 | 4093 |
| R(int) | 0.0770 | 0.1046 | 0.0625 | 0.1243 | 0.0465 |
| No. parameters / restraints | 771 / 13 | 899 / 48 | 385 / 0 | 300 / 109 | 632 / 522 |
| Final R1 values (I > 2σ(I)) | 0.0791 | 0.0598 | 0.0463 | 0.1249 | 0.0508 |
| Final *wR*(F^2^) values (all data) | 0.1949 | 0.1793 | 0.1320 | 0.3883 | 0.1378 |
| Goodness-of-fit on F^2^ | 1.059 | 1.046 | 1.049 | 1.071 | 1.114 |
| Largest difference peak & hole / e Å^–3^ | 0.501, -0.354 | 0.555, -0.251 | 0.246, -0.236 | 0.395, -0.381 | 0.164, -0.152 |

** The *SQUEEZE* algorithm has been applied.

**Donor-acceptor dihedral angles and corresponding bond lengths**

Donor-acceptor dihedral angles are given as the angle between the least-squares planes defined by the 1,8-naphthalimide (NAI) core (15 atoms) and either Cz (13 atoms), DMAC (14 atoms), or PXZ (14 atoms). For example:


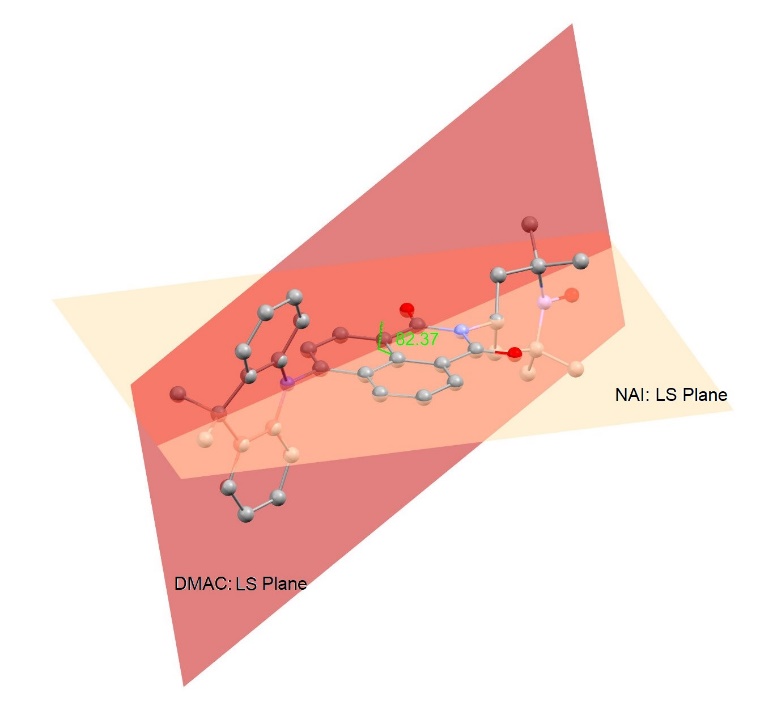


Uncertainties are produced using SHELXL. Due to the complex disorder in the structures of DMAC-Cy and PXZ-Cy, the values should be viewed as indicative.

**Table S2 Donor-acceptor dihedrals and corresponding bond lengths**

|  | **Donor-acceptor dihedral (°)** | **C–N bond length (Å)** |
| --- | --- | --- |
| Cz-Cy [molecule 1] | 67.18(7) | C21A–N1A: 1.412(8) |
| Cz-Cy [molecule 2] | 69.40(7) | C21B–N1B: 1.431(6) |
| Cz-TEMPO [molecule 1] | 49.04(4) | C21A–N1A: 1.415(3) |
| Cz-TEMPO [molecule 2] | 70.39(3) | C21B–N1B: 1.423(3) |
| DMAC-TEMPO | 82.37(2) | C16–N1: 1.4380(19) |
| DMAC-Cy [disorder 1] | 79.8(2) | C16–N1: 1.426(13) |
| DMAC-Cy [disorder 2] | 79.1(3) | C16A–N1A: 1.426(14) |
| PXZ-Cy [disorder 1] | 86.71(14) | C16–N1: 1.433(4) |
| PXZ-Cy [disorder 2] | 83.90(18) | C16A–N1A: 1.434(5) |

**
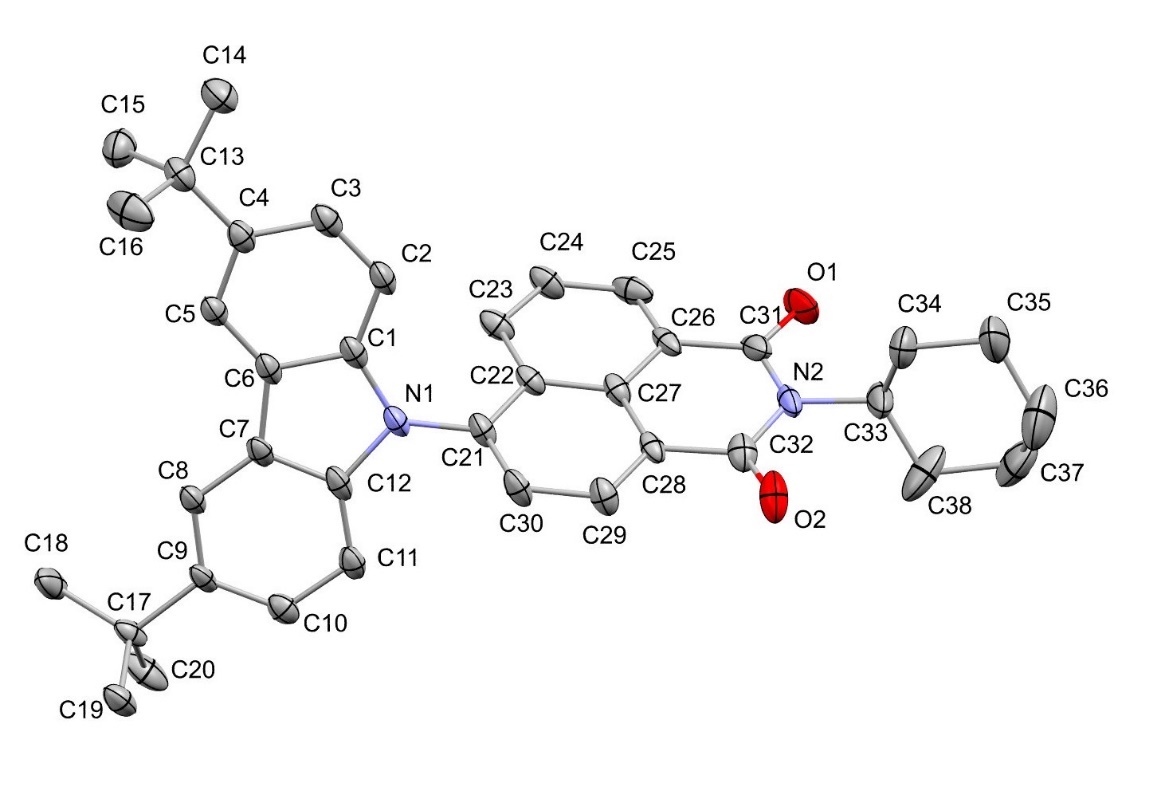
**

**Fig. S6** Molecular structure of Cz-Cy showing displacement ellipsoids at 50% probability. H atoms are omitted. Only one of two crystallographically independent molecules is shown; the other is closely comparable.

**
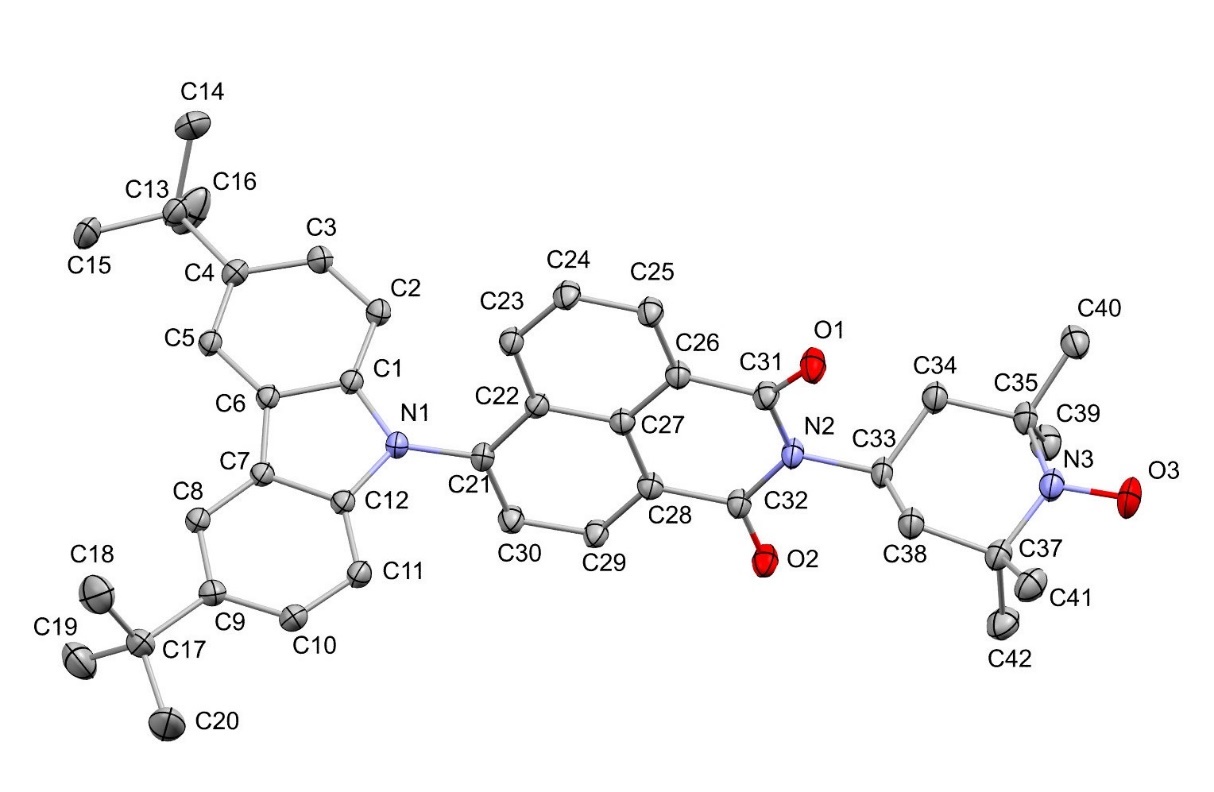
**

**Fig. S7** Molecular structure of Cz-TEMPO showing displacement ellipsoids at 50% probability. H atoms are omitted. Only one of two crystallographically independent molecules is shown; the other is closely comparable.

**
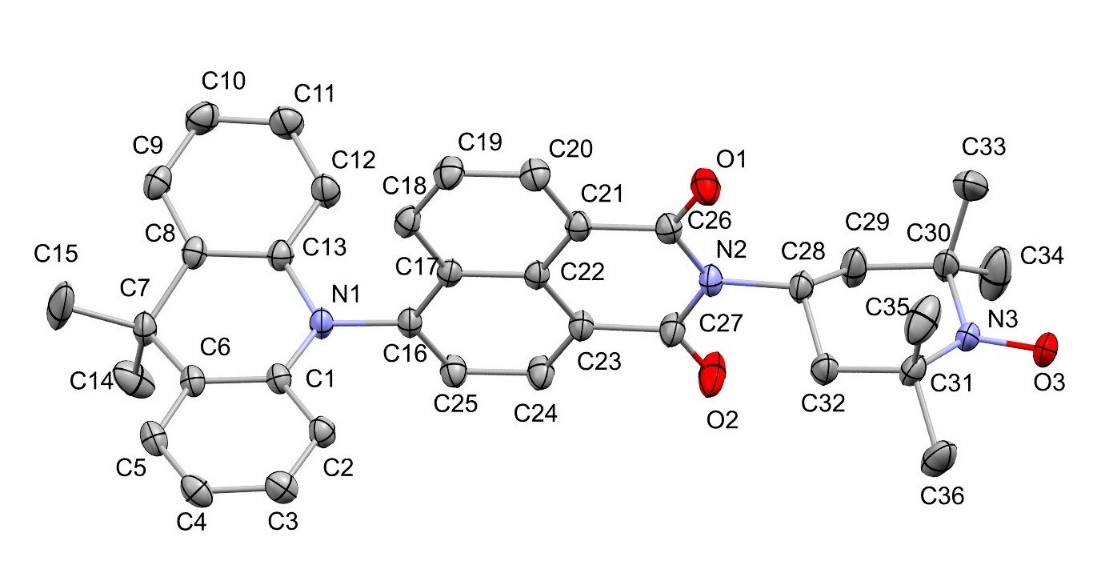
**

**Fig. S8** Molecular structure of DMAC-TEMPO showing displacement ellipsoids at 50% probability. H atoms are omitted.

**
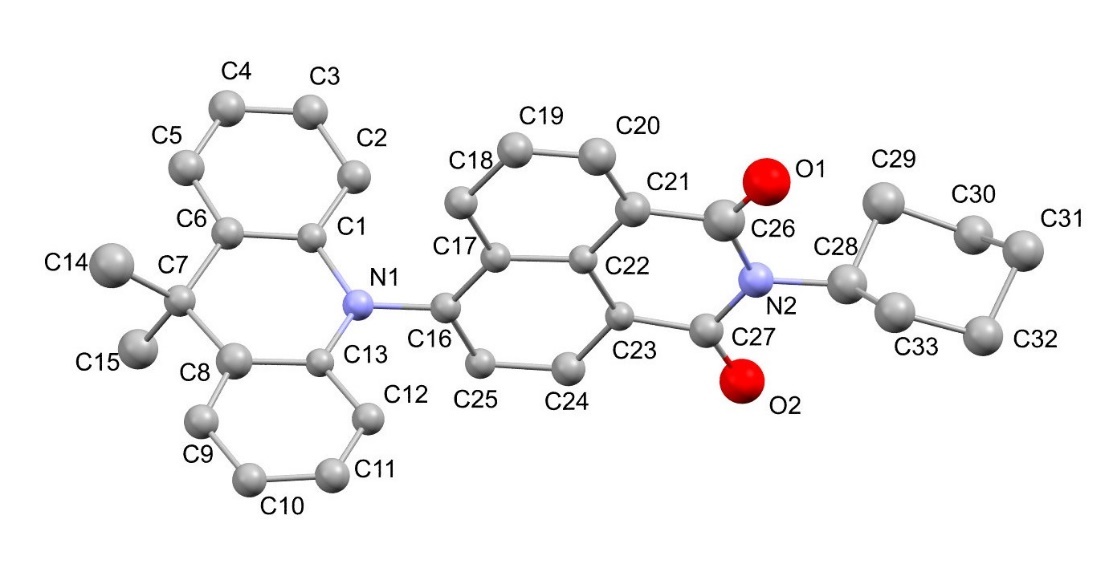
**

**Fig. S9** Molecular structure of DMAC-Cy showing (isotropic) displacement ellipsoids at 50% probability. H atoms are omitted. The crystal structure shows whole-molecule disorder; only one of two orientations of the molecule is shown.

**
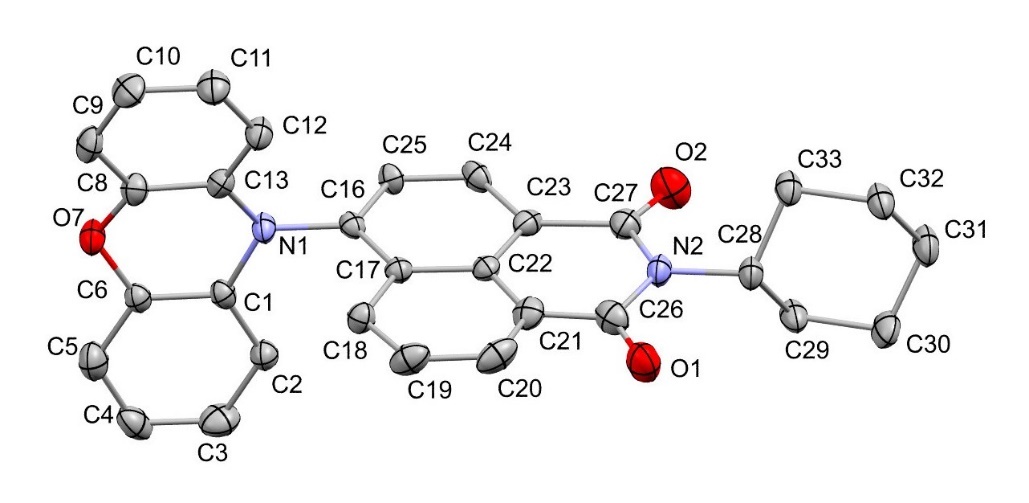
**

**Fig. S10** Molecular structure of PXZ-Cy showing displacement ellipsoids at 50% probability for non-H atoms. The crystal structure shows whole-molecule disorder; only one of two orientations of the molecule is shown.

| **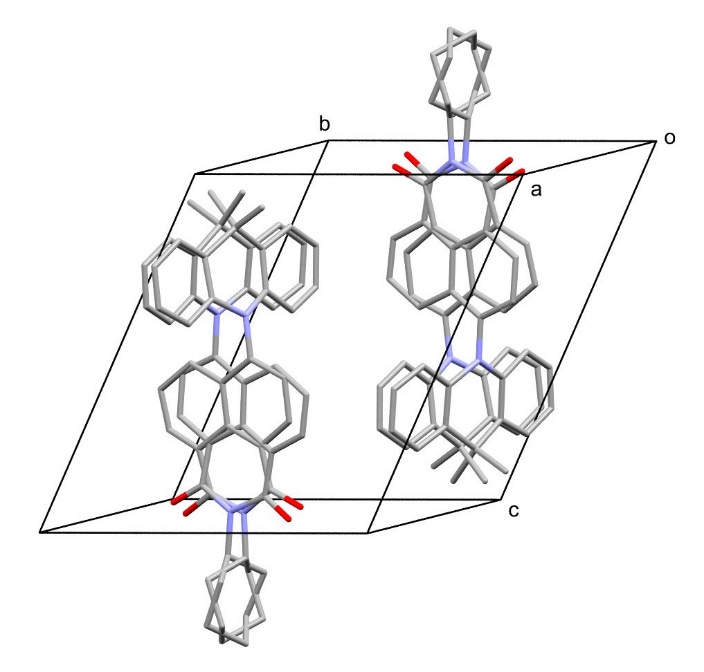**  **a** | **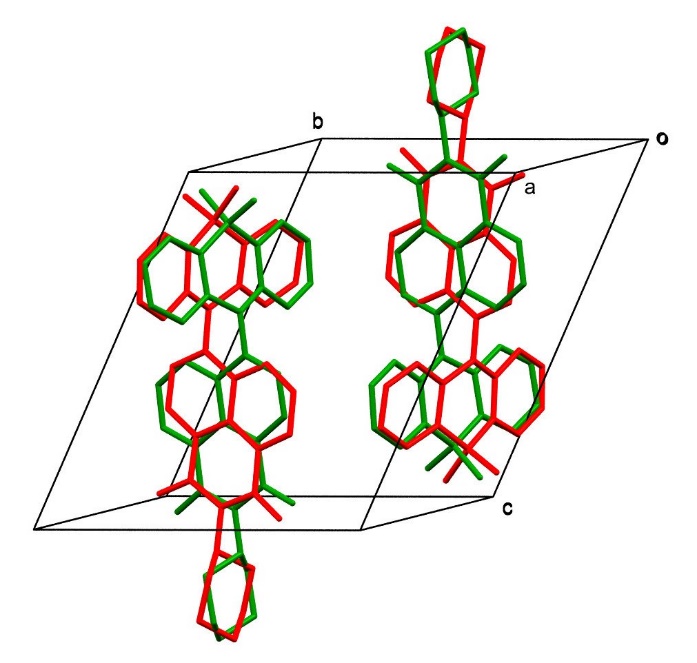**  **b** |
| --- | --- |

**Fig. S11** **a**, Refined unit-cell contents for DMAC-Cy, showing the two orientations of the molecule in each molecular site. **b**, Overlay of two extracted crystal structures after periodic (plane-wave) DFT optimisation. The agreement between the refined X-ray coordinates and the DFT structures is good.

| **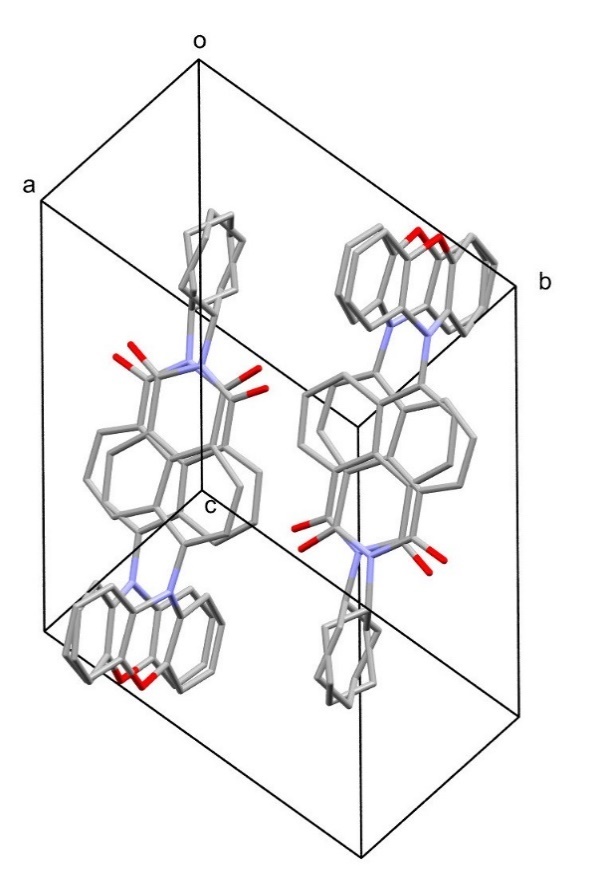**  **a** | **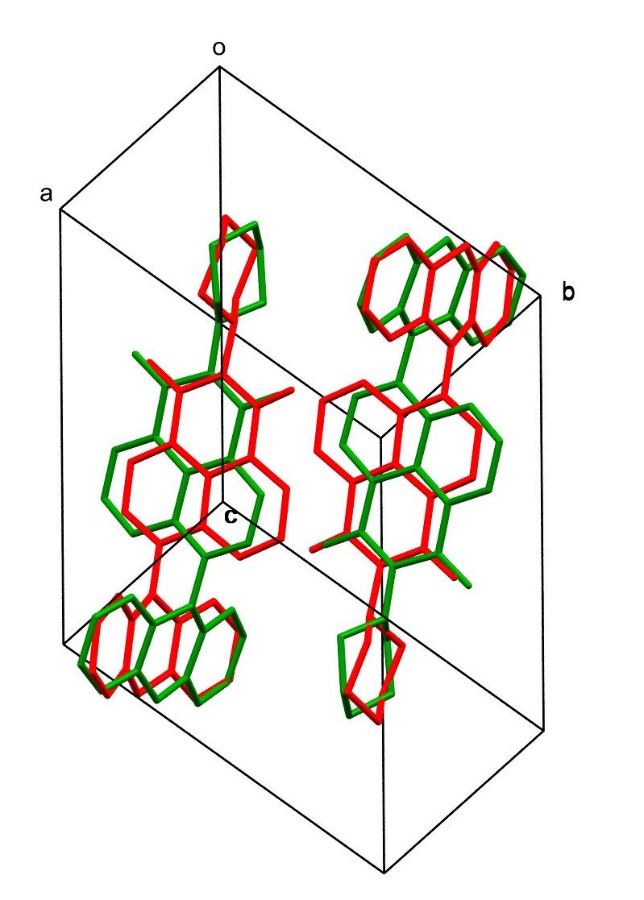**  **b** |
| --- | --- |

**Fig. S12** **a**, Refined unit-cell contents for PXZ-Cy, showing the two orientations of the molecule in each molecular site. **b**, Overlay of two extracted crystal structures after periodic (plane-wave) DFT optimisation. The agreement between the refined X-ray coordinates and the DFT structures is good.

# Cyclic voltammetry


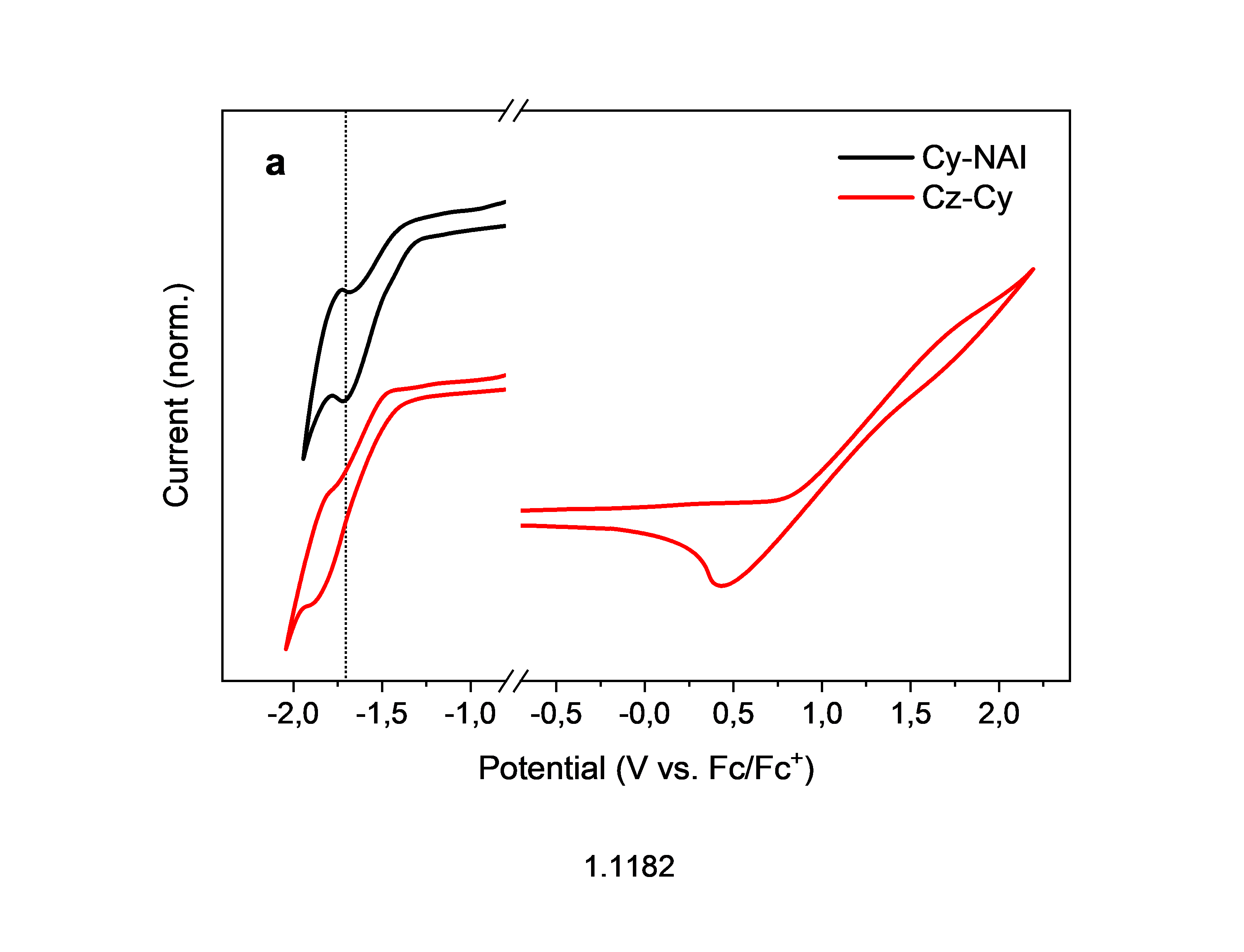

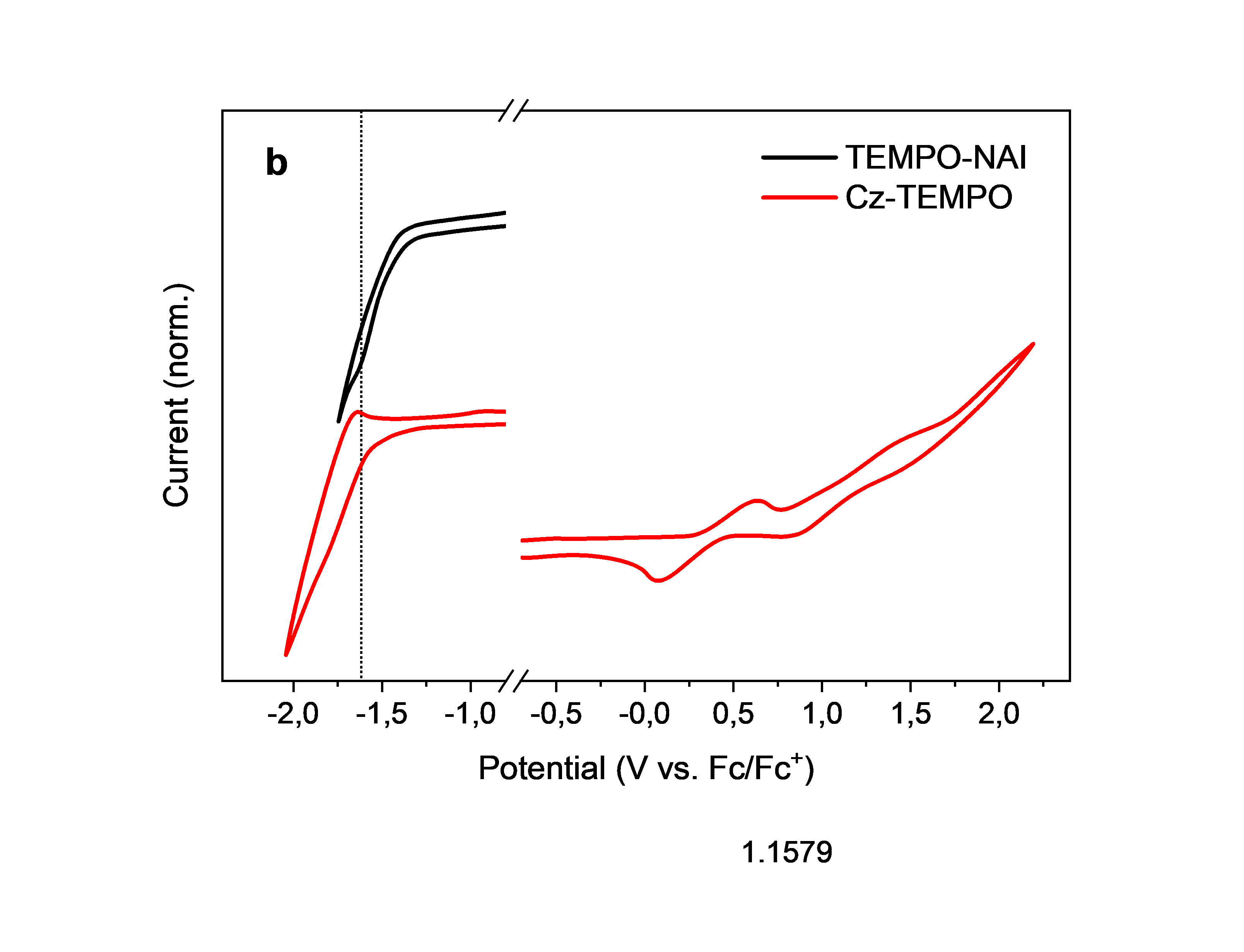


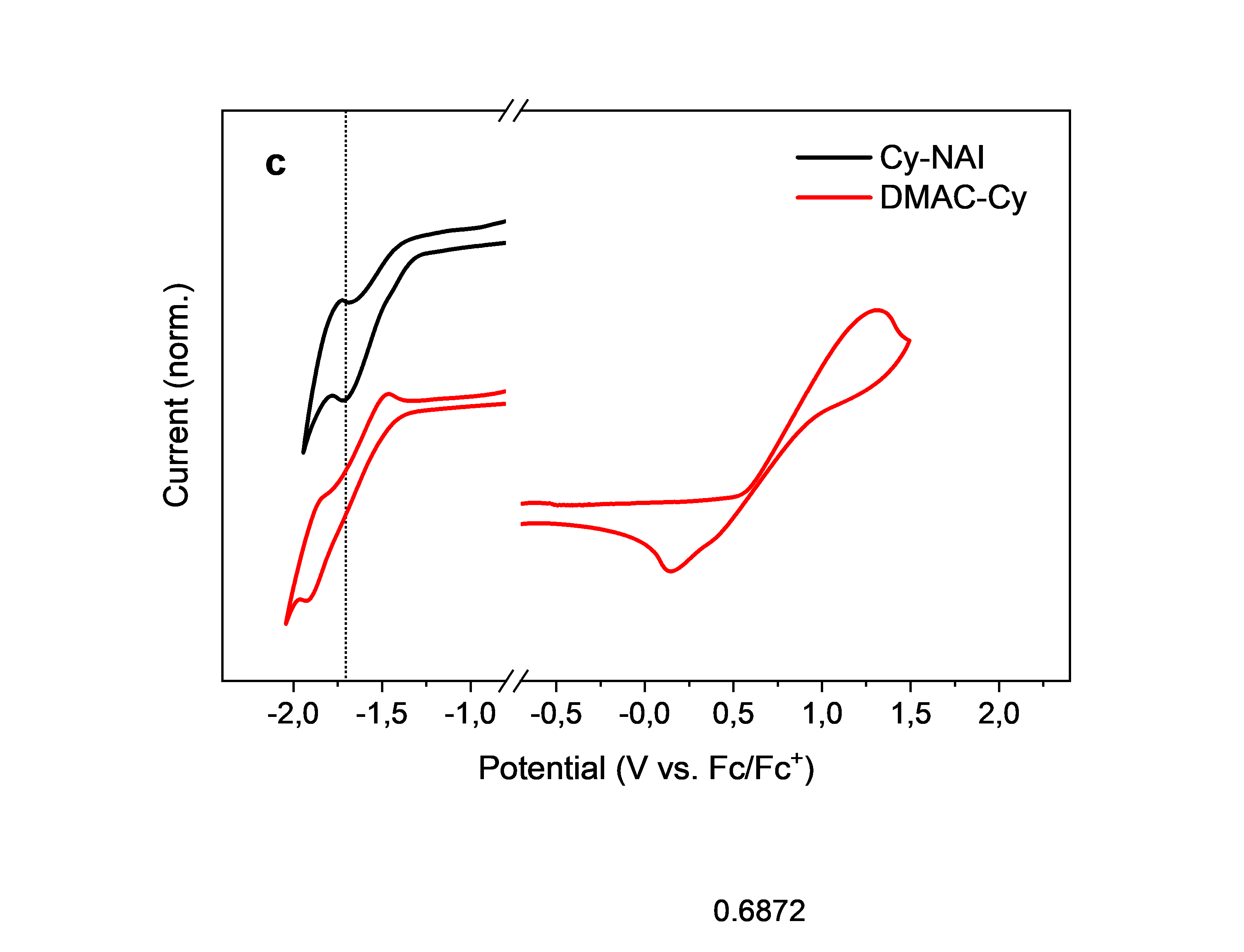

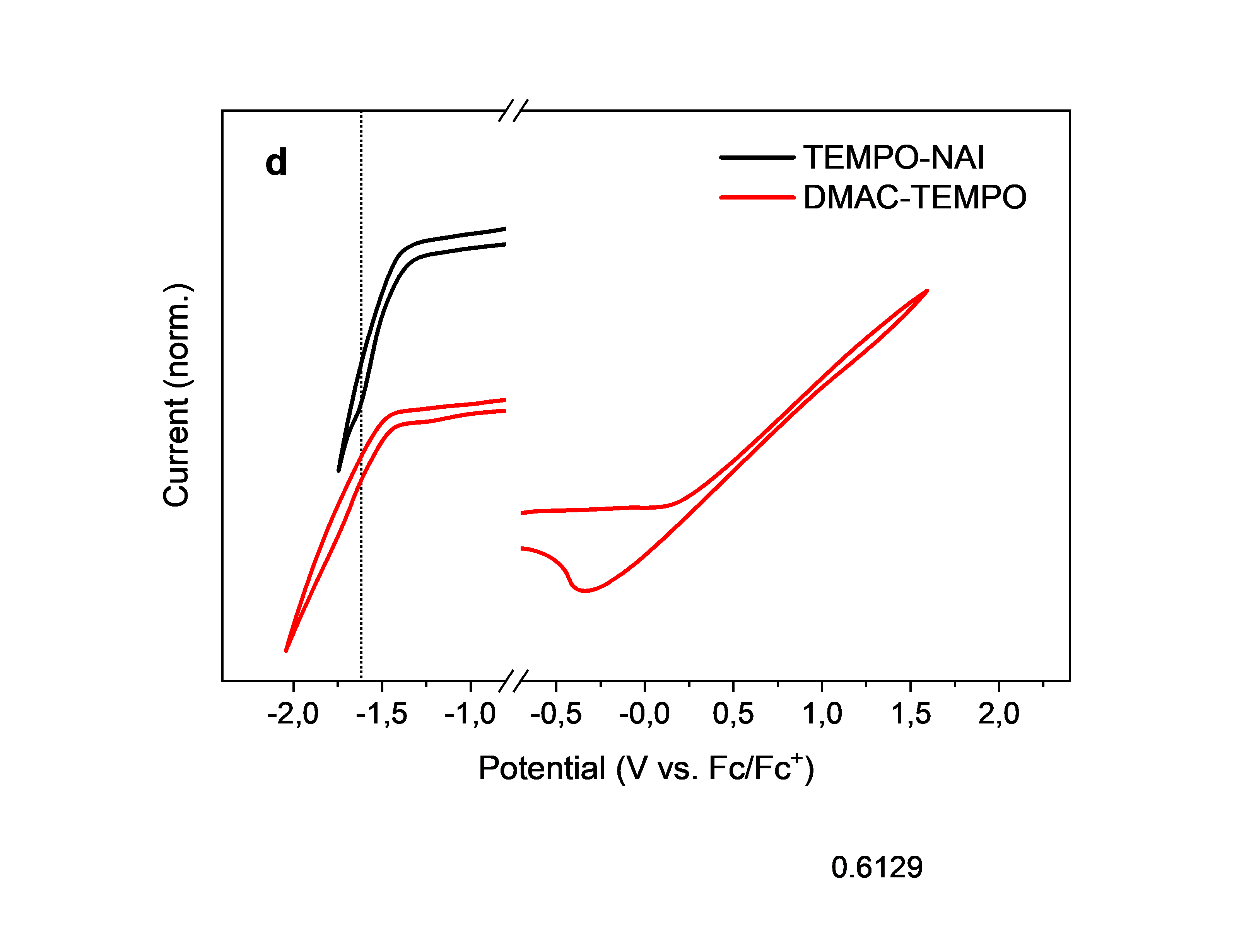


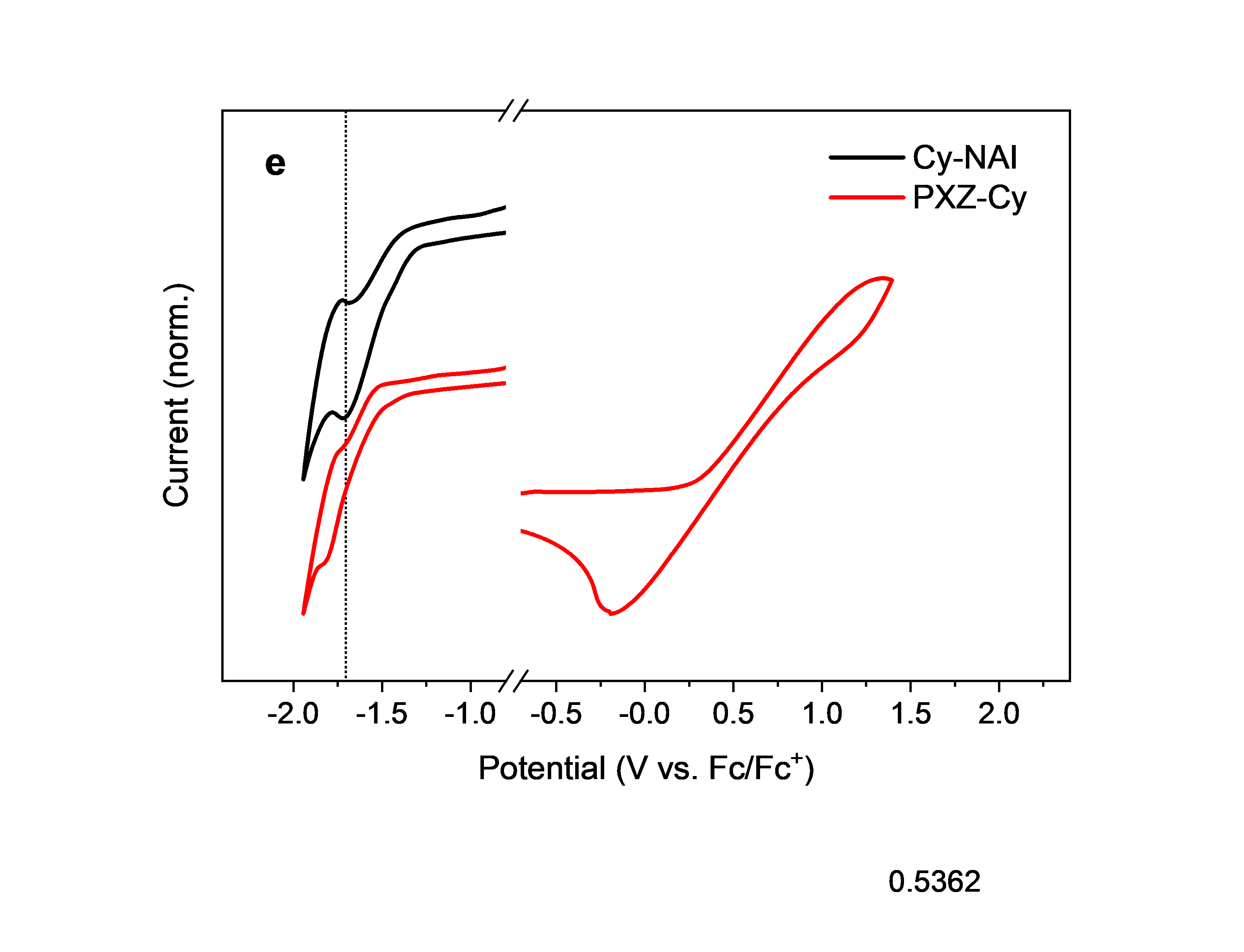

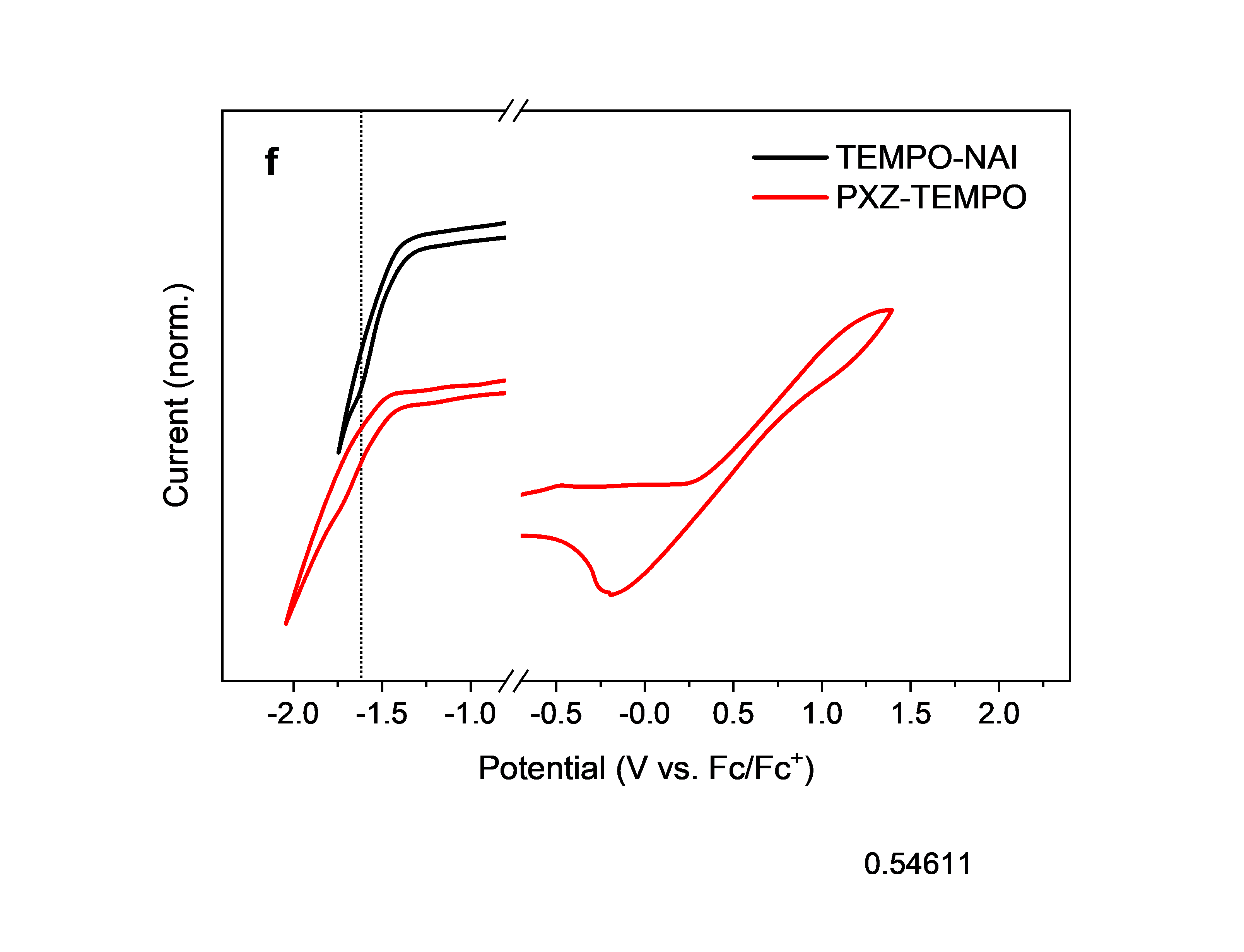


**Fig. S13** Cyclic voltammetry traces for **a**,**c**,**e**, TADF and **b**,**d**,**f**, TADF-TEMPO molecules (red traces). Oxidation and reduction reactions were measured separately in the anodic and cathodic range, respectively, due to a more intense oxidation wave with respect to the reduction. Dashed vertical line indicates a shift in reduction potential by less than 0.1 V when going from acceptor structure Cy-NAI (**a**,**c**,**e**) to TEMPO-NAI (**b**,**d**,**f**) (black traces). The redox reaction of TEMPO radical is centred at 0.3 V,^[11]^ and it is visible in Cz-TEMPO (**b**) where it is not overlapping with the oxidation of the donor unit at higher potential.

Cyclic voltammetry was carried out on a PalmSens EmStat4S potentiostat in a three-electrode setup using a glassy carbon electrode (3.0 mm diameter) as the working electrode, platinum wire as the counter electrode and freshly activated silver wire as the Ag/Ag^+^ reference electrode. The silver wire was activated by immersing in concentrated HCl solution to remove any silver oxides or other impurities, then rinsed with water and acetone and dried prior to each measurement. The reference electrode was calibrated against ferrocene/ferrocenium (Fc/Fc^+^) redox couple at the end of each measurement (the Fc/Fc^+^ half-wave potential, E_1/2_, was determined at 0.20 V vs. Ag/Ag^+^). The supporting electrolyte was 0.1 M solution of Bu_4_NPF_6_ in anhydrous THF and the scan rate was 0.1 V s^–1^. The electrolyte was bubbled with Ar gas before each measurement to remove any dissolved oxygen. Sample concentration was in the order of 10^–5^ M.

# Photophysics

**
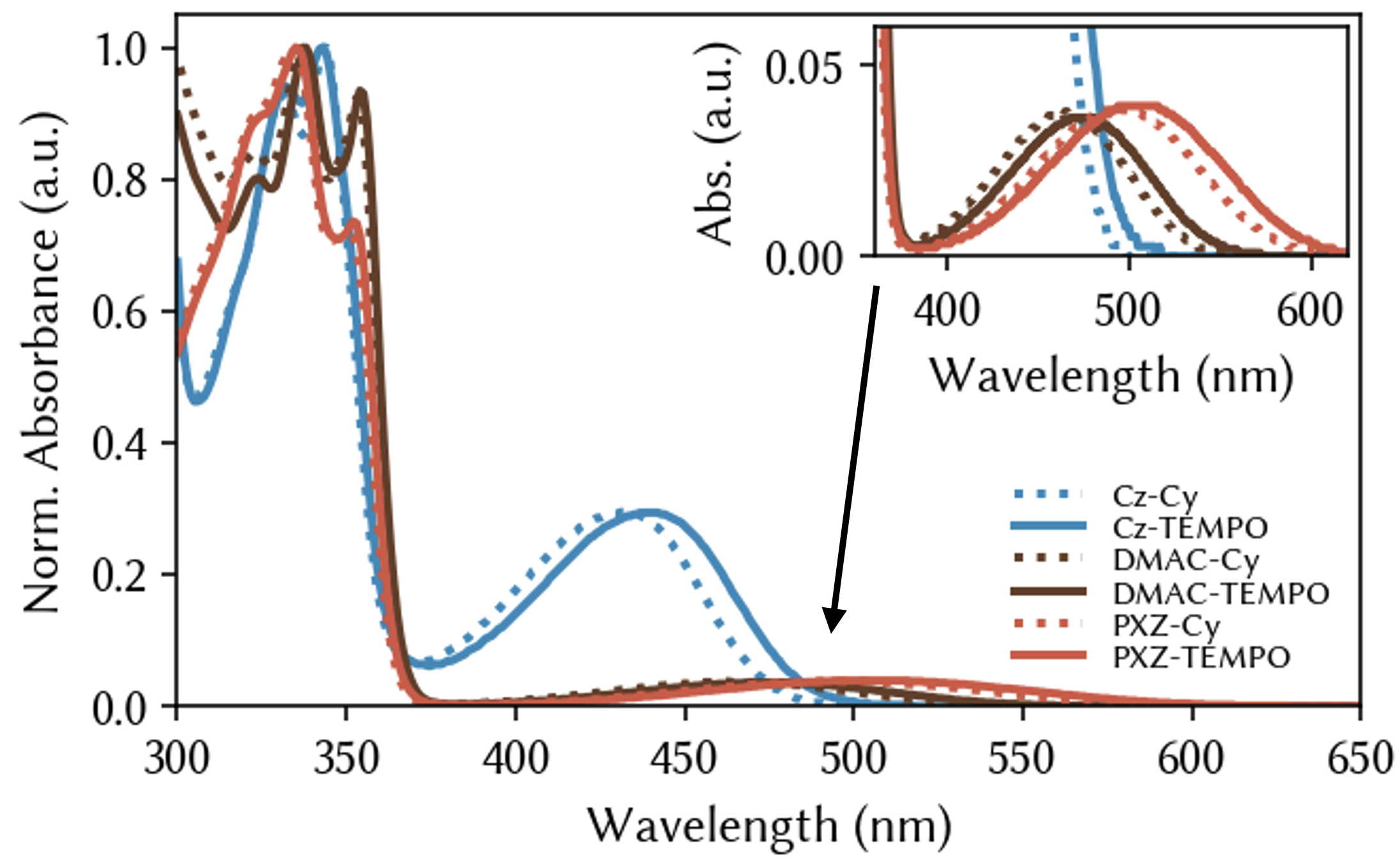
**

**Fig. S14** Steady-state absorption spectra of 100 µM toluene solutions at 293 K. Inset shows a magnified region at the band onset from the same dataset.


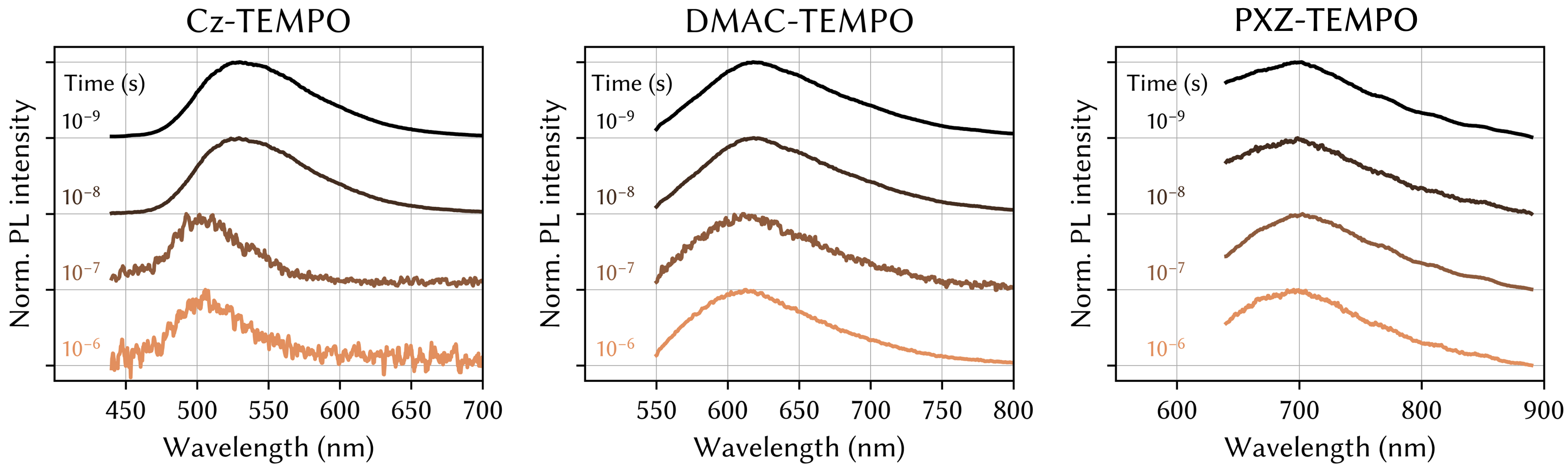


**Fig. S15** Transient Photoluminescence (trPL) time-gated spectra of 100 µM toluene solutions following excitation with 415 nm pulses at 293 K.

**
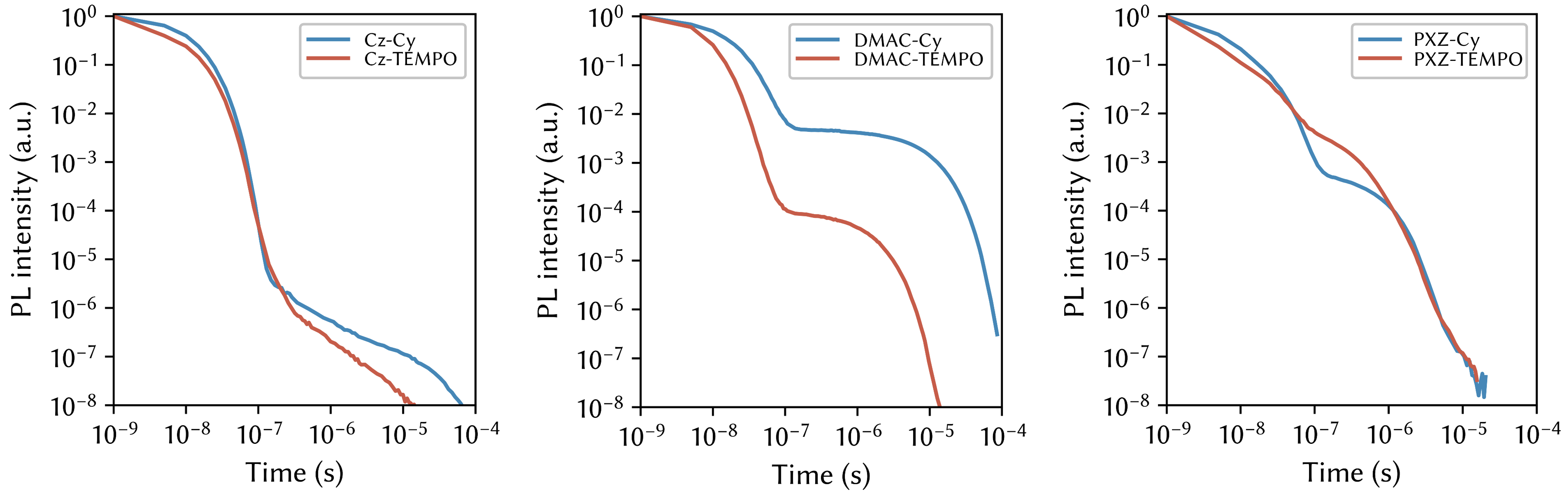
**

**Fig. S16** Transient Photoluminescence (trPL) traces of 100 µM toluene solutions following excitation with 415 nm pulses at 293 K. Data is normalised to the peak populations. The traces arise from summing across the whole emission line.


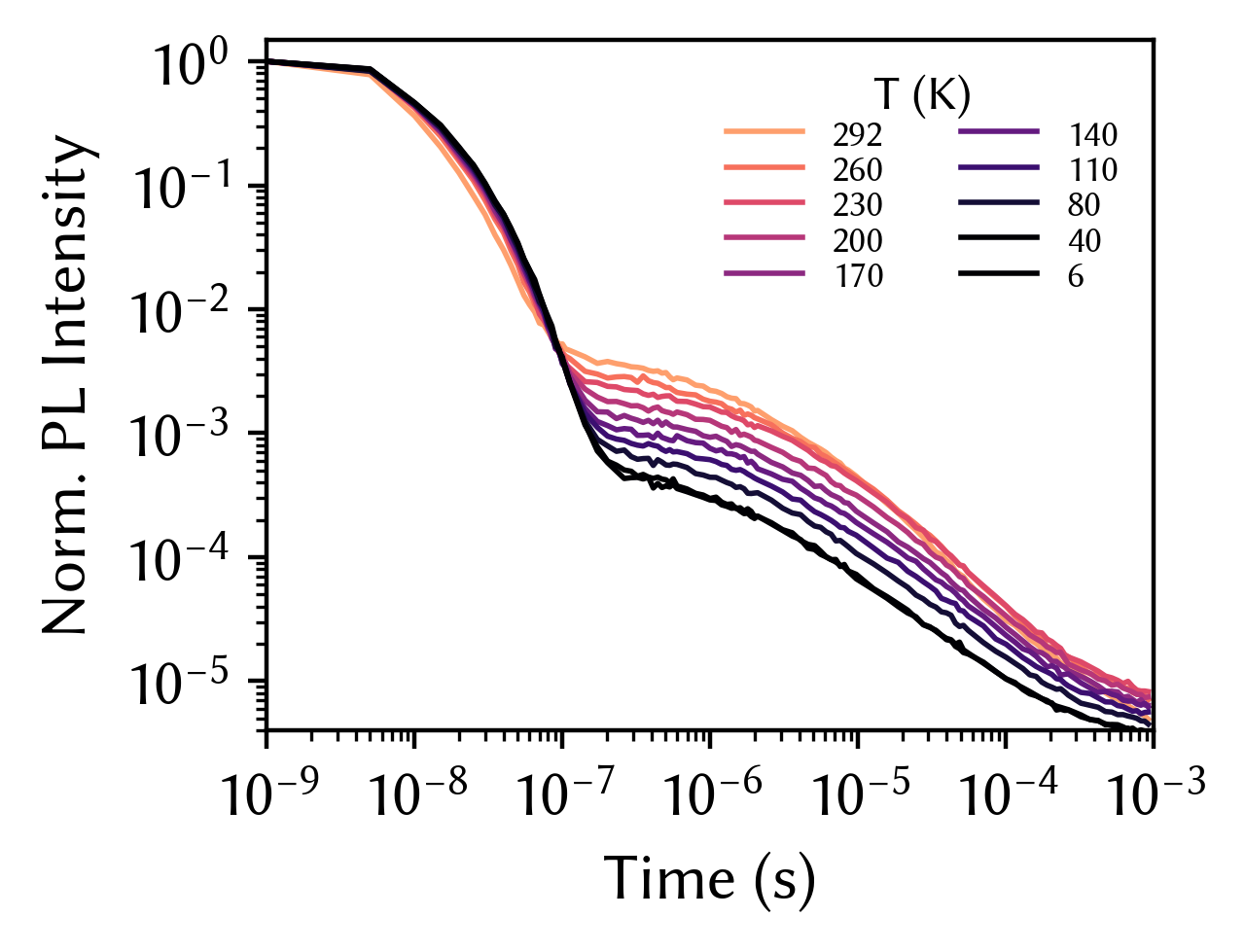


**Fig. S17** Transient Photoluminescence (trPL) time-gated kinetics of 1% DMAC-TEMPO in PMMA film following excitation with 415 nm 3.2 µJ/cm^2^ pulses. The integrated data from this experiment is plotted in Main Text Fig. 2c.


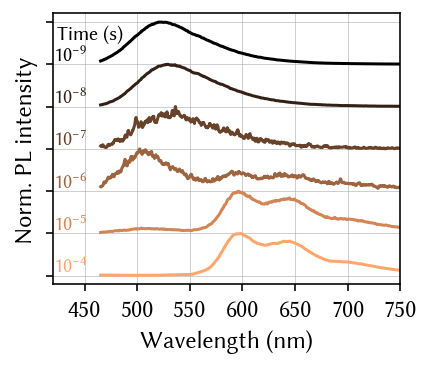


**Fig. S18** Transient Photoluminescence (trPL) time-gated spectra of 1% Cz-TEMPO in PMMA films following excitation with 415 nm 3.2 µJ/cm^2^ pulses at 10 K. Structured, redshifted and delayed emission is due to phosphorescence from the ^2^D_0_T_1_^(LE)^ state.

**
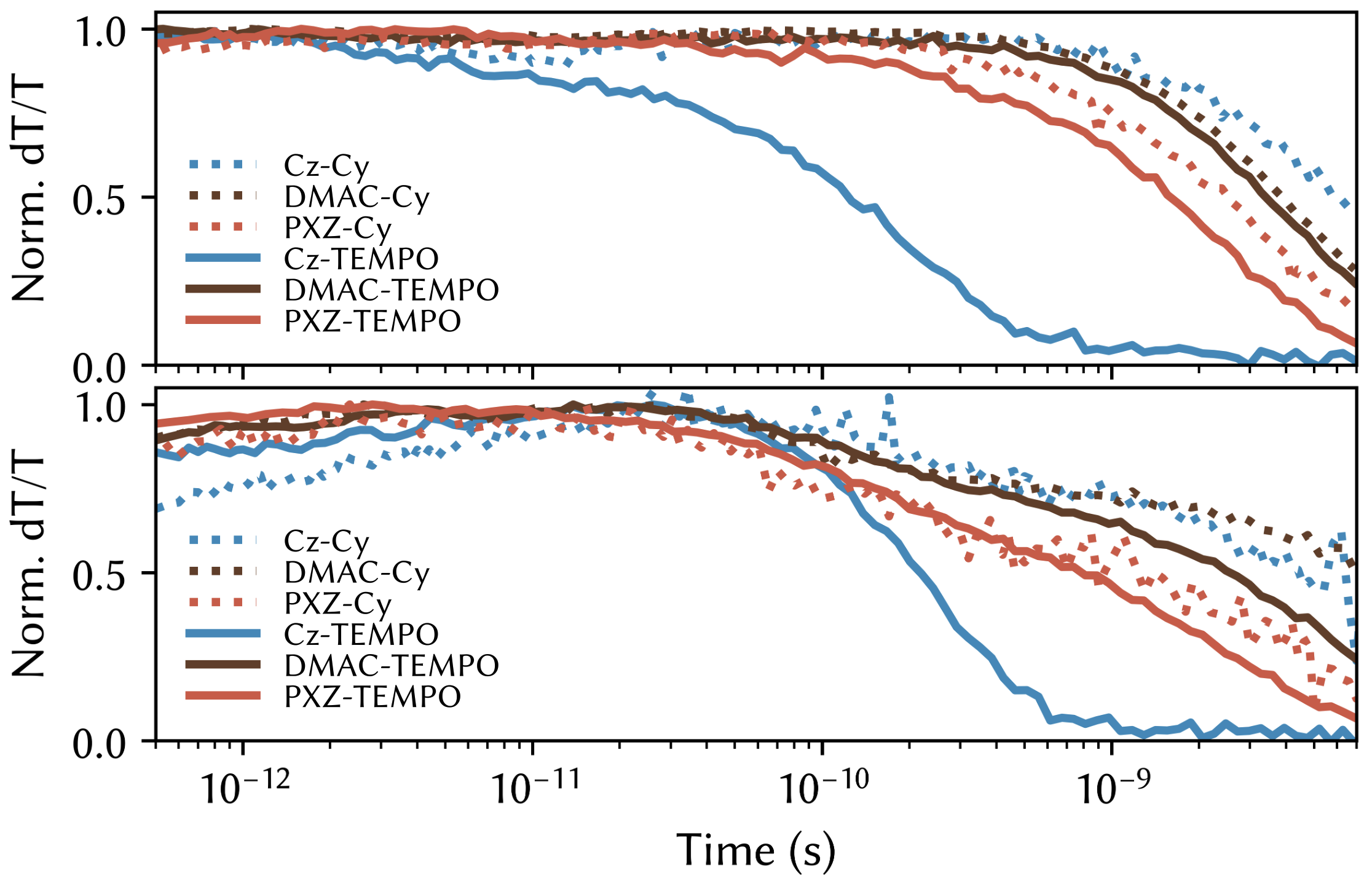
**

**Fig. S19** Transient Absorption (TA) kinetic traces of 100 µM toluene solutions at 293 K. **a,** The UV (370-500 nm) probe range dataset was acquired following excitation with 530 nm 28 µJ/cm^2^ pulses. **b,** The visible (550-950 nm) probe range dataset was acquired following excitation with 470 nm 21 µJ/cm^2^ pulses. Data is normalised to the peak populations.


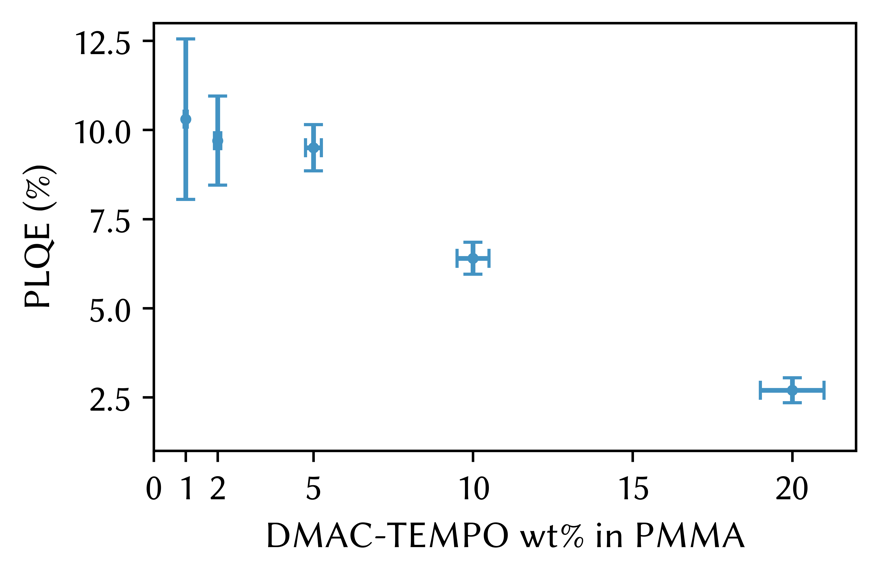


**Fig. S20** Concentration dependence of PLQE in PMMA films of DMAC-TEMPO. Measurement on encapsulated films at room temperature under continuous 375 nm excitation at fluence of 6 mW/cm^2^.


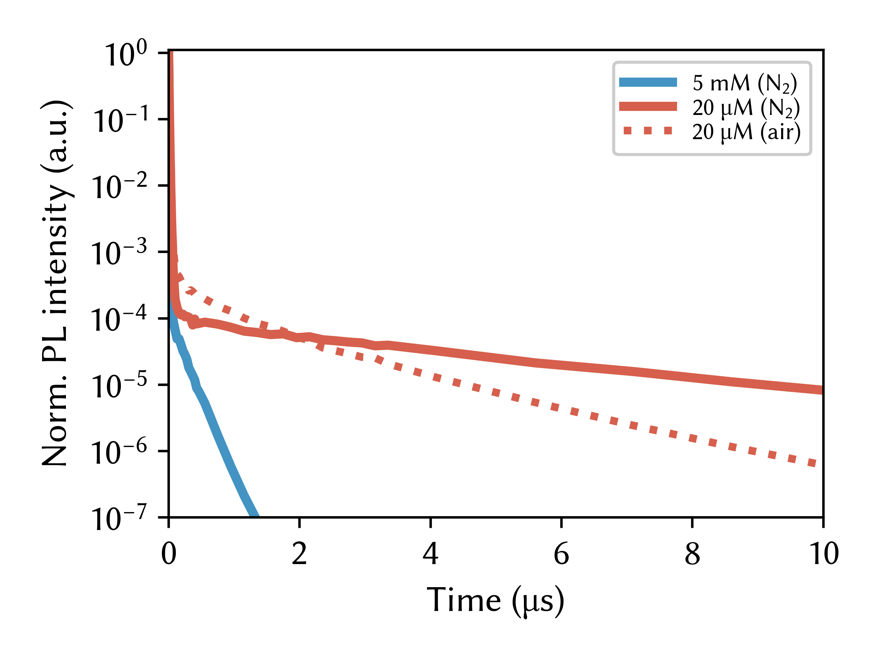


**Fig. S21** Concentration dependence of emission kinetics in toluene solutions of DMAC-TEMPO. Performed under pulsed 415 nm excitation at fluence of 3.2 µJ/cm2 at 293 K.


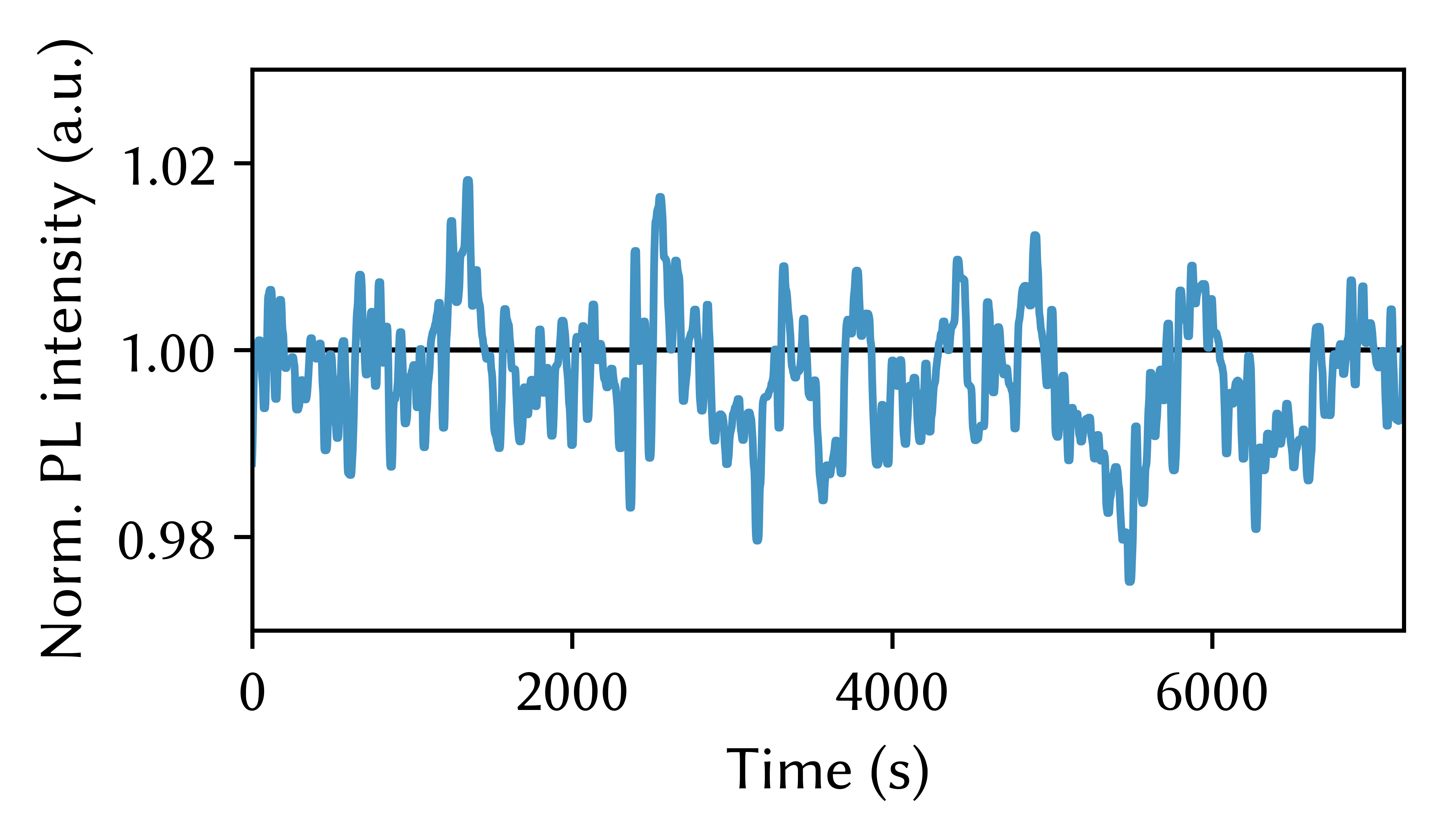


**Fig. S22** Photostability of 50 µM toluene solution of DMAC-TEMPO. Performed under cw 375 nm excitation at fluence of 108 W/cm^2^ at 293 K.

# Electron Spin Resonance


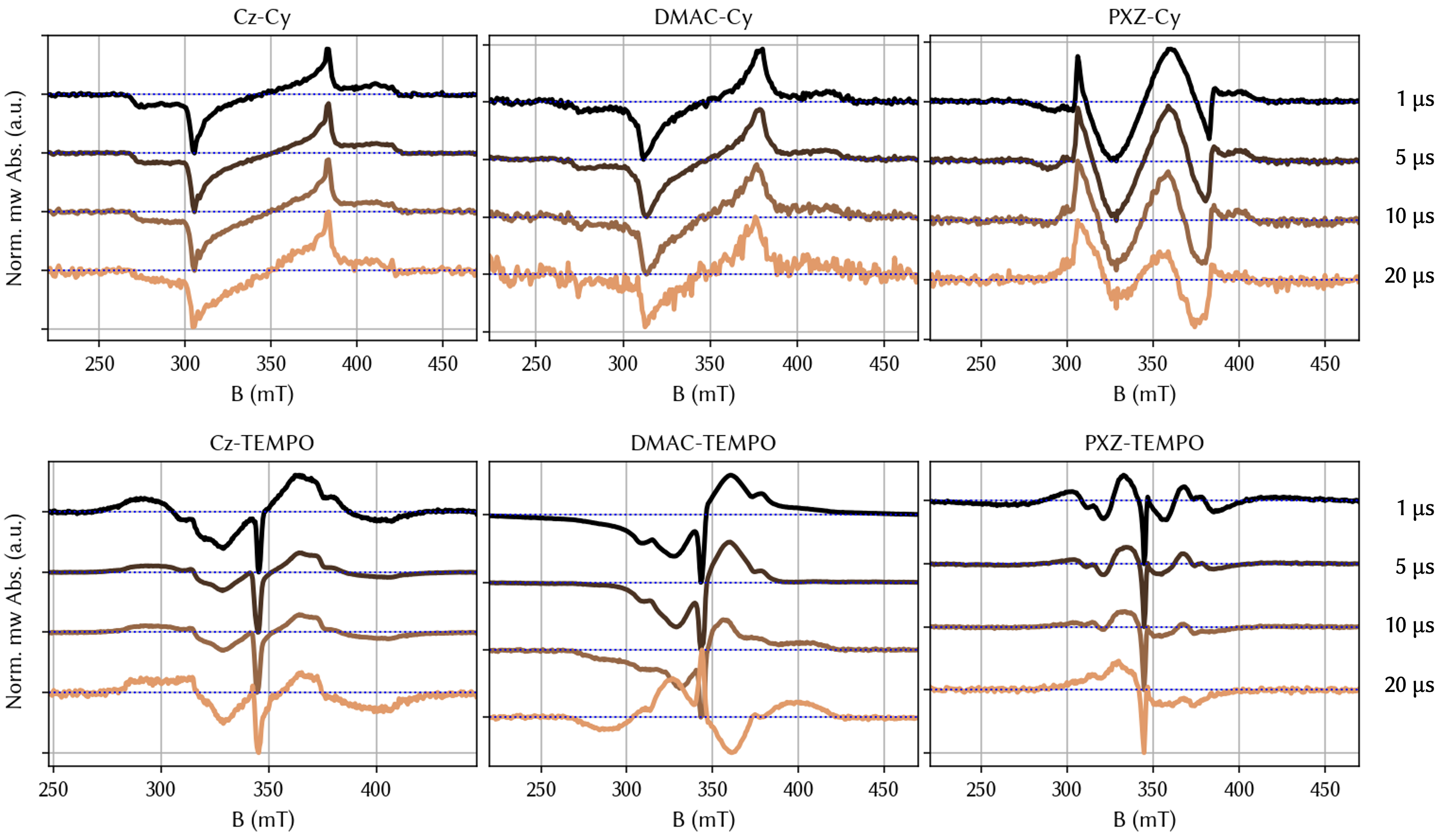


**Fig. S23** Time evolution of X-band trESR spectra, performed on 100 µM toluene solutions after 450 nm 0.5 mJ excitation at 80 K. In Cz-Cy and DMAC-Cy the signal does not evolve with time, consistent with a single (LE) triplet species having an accumulated population at µs timescales. In PXZ-Cy the signal consists of two triplet species are visible and their population ratio shifts towards the LE triplet at later times. In the TEMPO-substituted compounds, more complex time evolution is observed. In Cz-TEMPO the lineshape does not change with time. In both DMAC-TEMPO and PXZ-TEMPO the late times species shows a growing contribution of the CT quartet, which is repopulated during RISC.


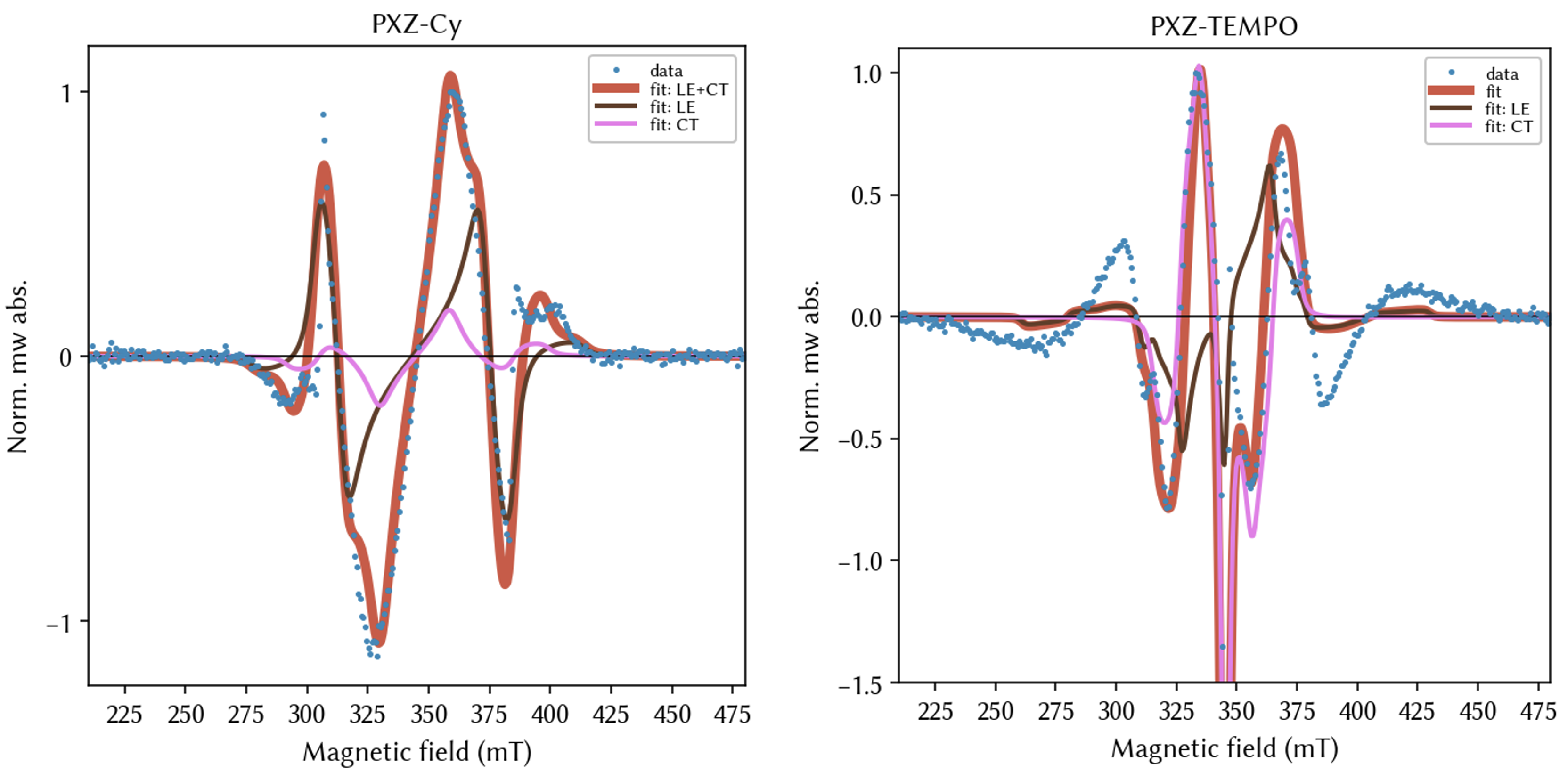


**Fig. S24** Fitting of prompt X-band trESR data of PXZ-Cy (Fig. 3c) and PXZ-TEMPO (Fig. 3f) showing the lineshapes of the 2 contributing species associated with the LE and CT excitons.


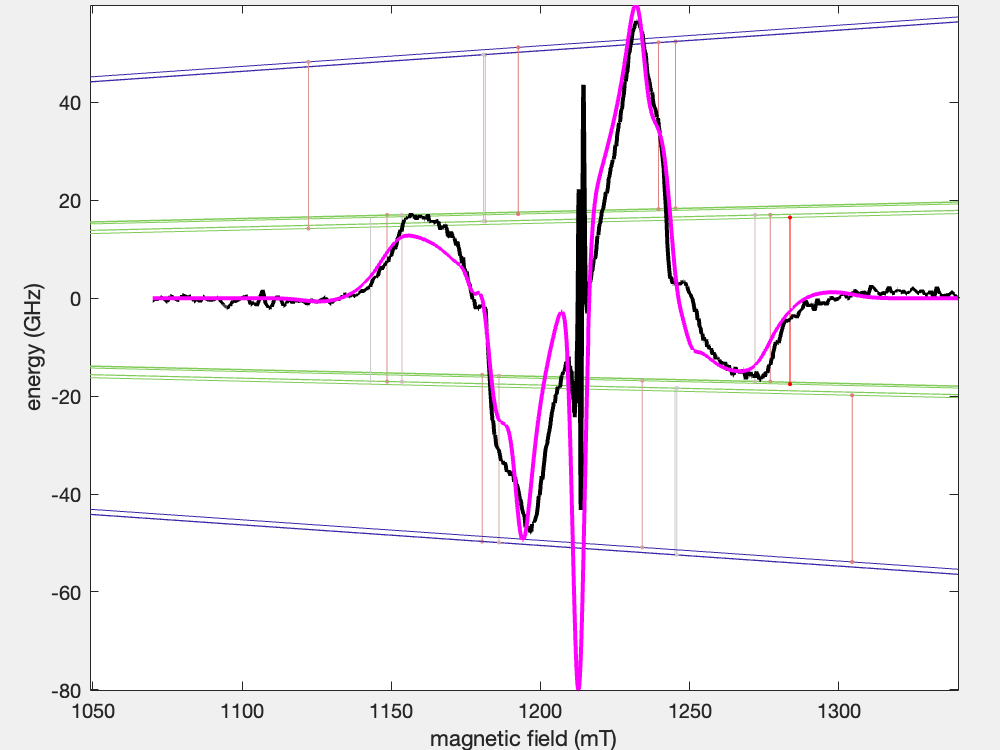


**Fig. S25** Q-band trESR on frozen 100 µM toluene solution of Cz-TEMPO 1 µs after 450 nm 0.5 mJ excitation at 80 K. Data (black) and simulation (pink) are superimposed on the calculated energy eigenstates along the x,y,z canonical orientations.


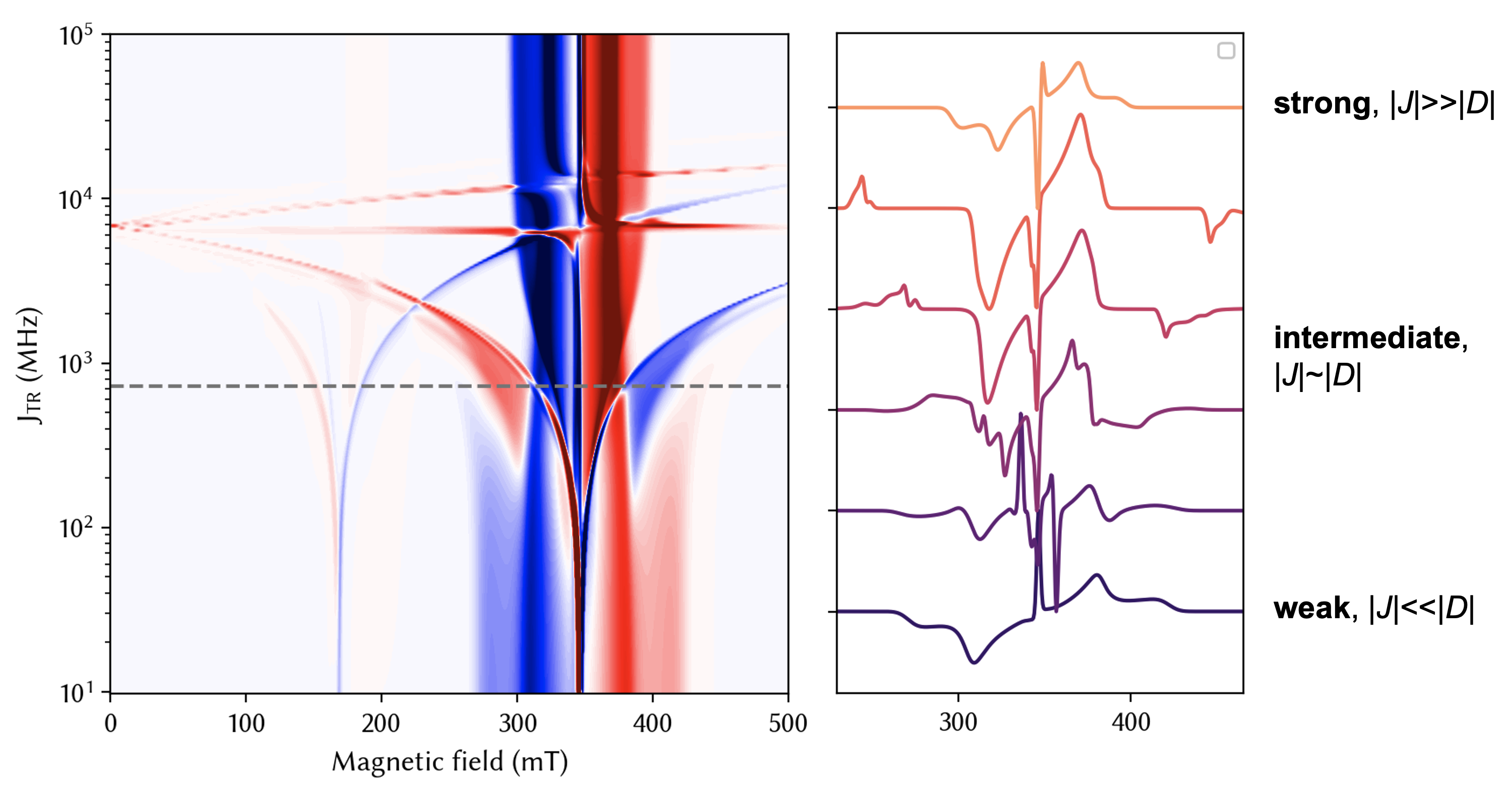
 **Fig. S26** Calculated lineshapes of prompt X-band trESR signals from the ^2,4^D_0_T_1_^(LE)^ states in Cz-TEMPO and DMAC-TEMPO as a function radical-triplet exchange strength, *J*_TR_. Dashed line corresponds to *J*_TR_ which yields the best fit to experimental traces in Fig. 3d.


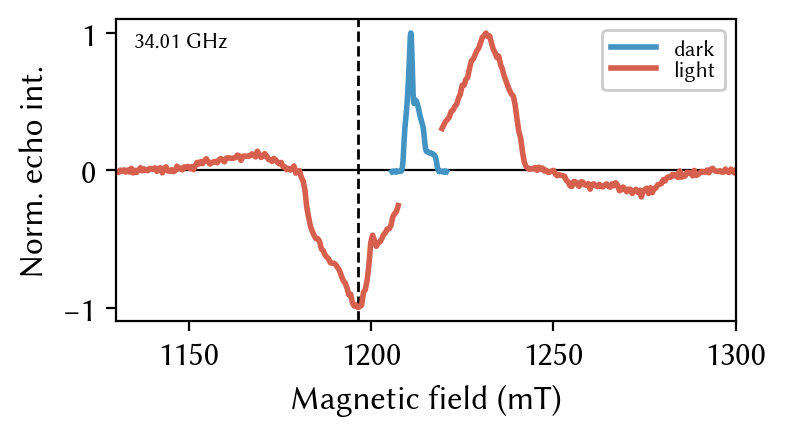


**Fig. S27** Q-band light-induced pulsed ESR on frozen 100 µM toluene solution of Cz-TEMPO at 80 K. Echo detected spectra with and without light excitation. Dashed line shows field position of *g* = 2.033. Light induced signal collected at DAF = 2 µs after 450 nm 0.5 mJ excitation.


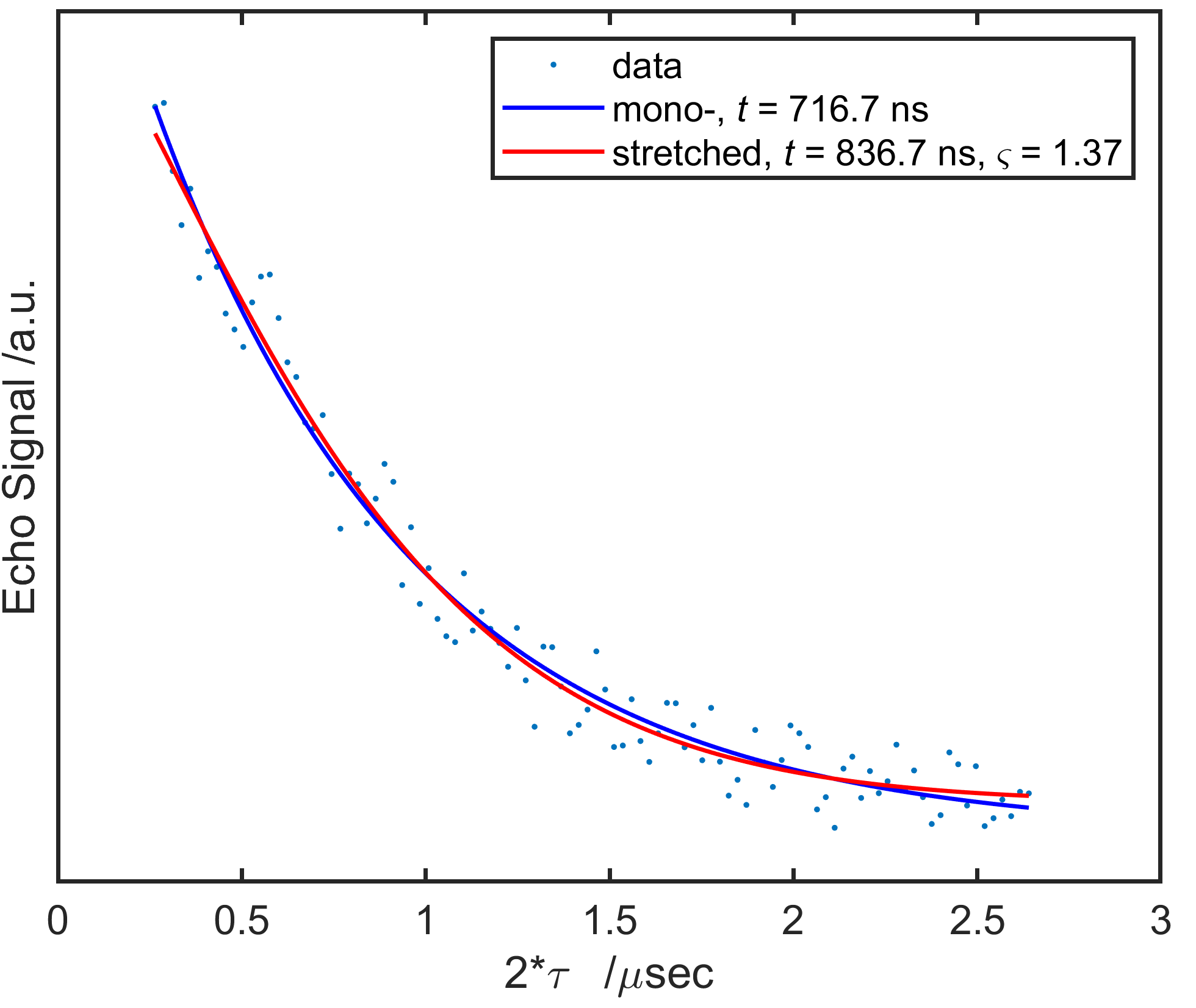


**Fig. S28** Coherence time measurement at Q-band on frozen 100 µM toluene solution of Cz-TEMPO at 80 K and at *g* = 2.033. Performed at DAF = 2 µs after 450 nm 0.5 mJ excitation.


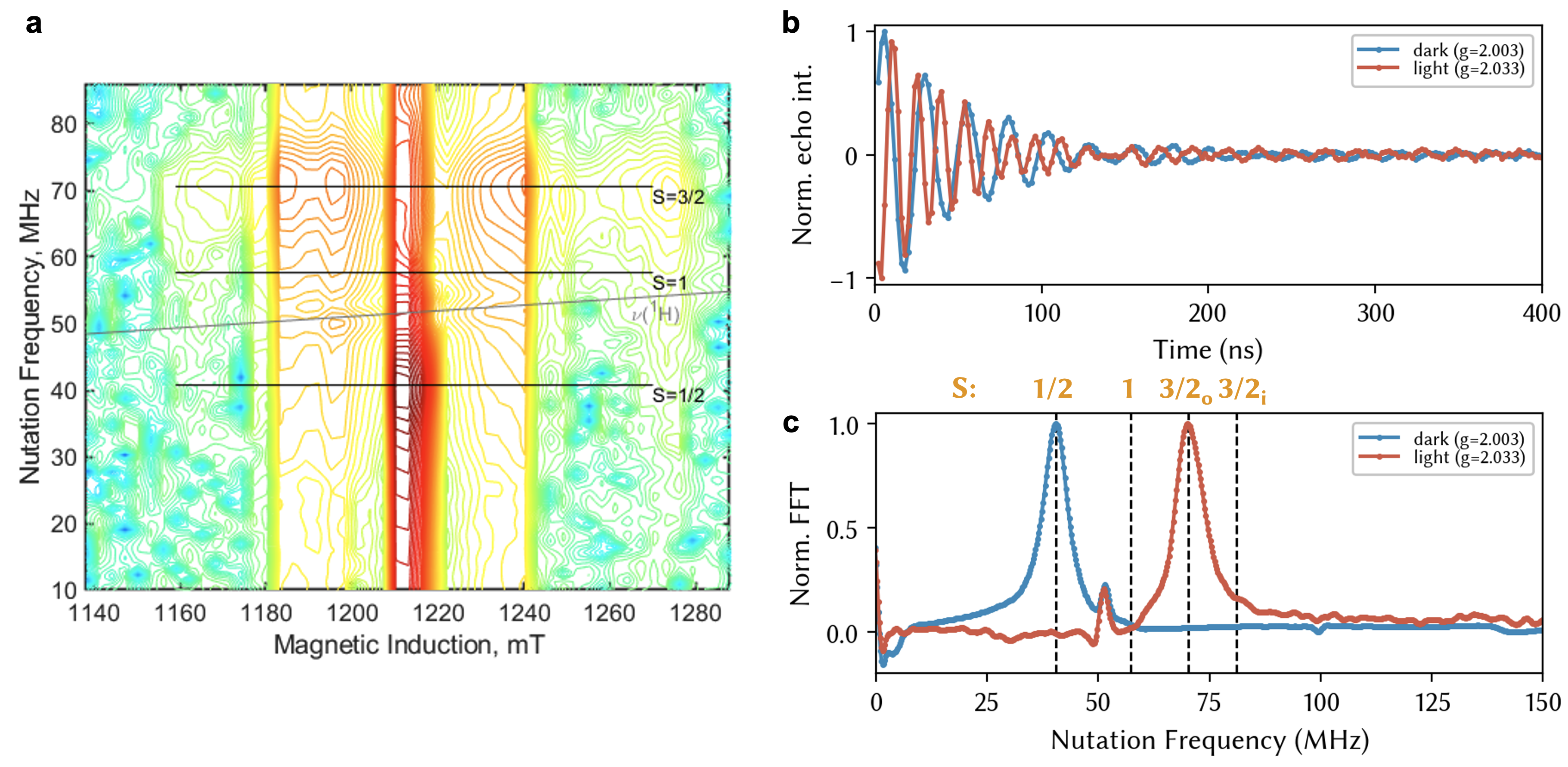


**Fig. S29** Rabi nutations measurement at Q-band on frozen 100 µM toluene solution of Cz-TEMPO at 80 K at DAF = 2 µs after 450 nm 0.5 mJ excitation. **a,** Field-dependence of transient nutations. **b,** Time-domain cut of the echo intensity showing Rabi oscillations on the central TEMPO radical resonance in the dark, and multiplet polarisation after light excitation. **c,** FFT of time-domain traces, revealing that the multiplet polarisation arises from a quartet rather than a triplet state. The small signal at 52 MHz is due to Larmor precession of ^1^H nuclei.


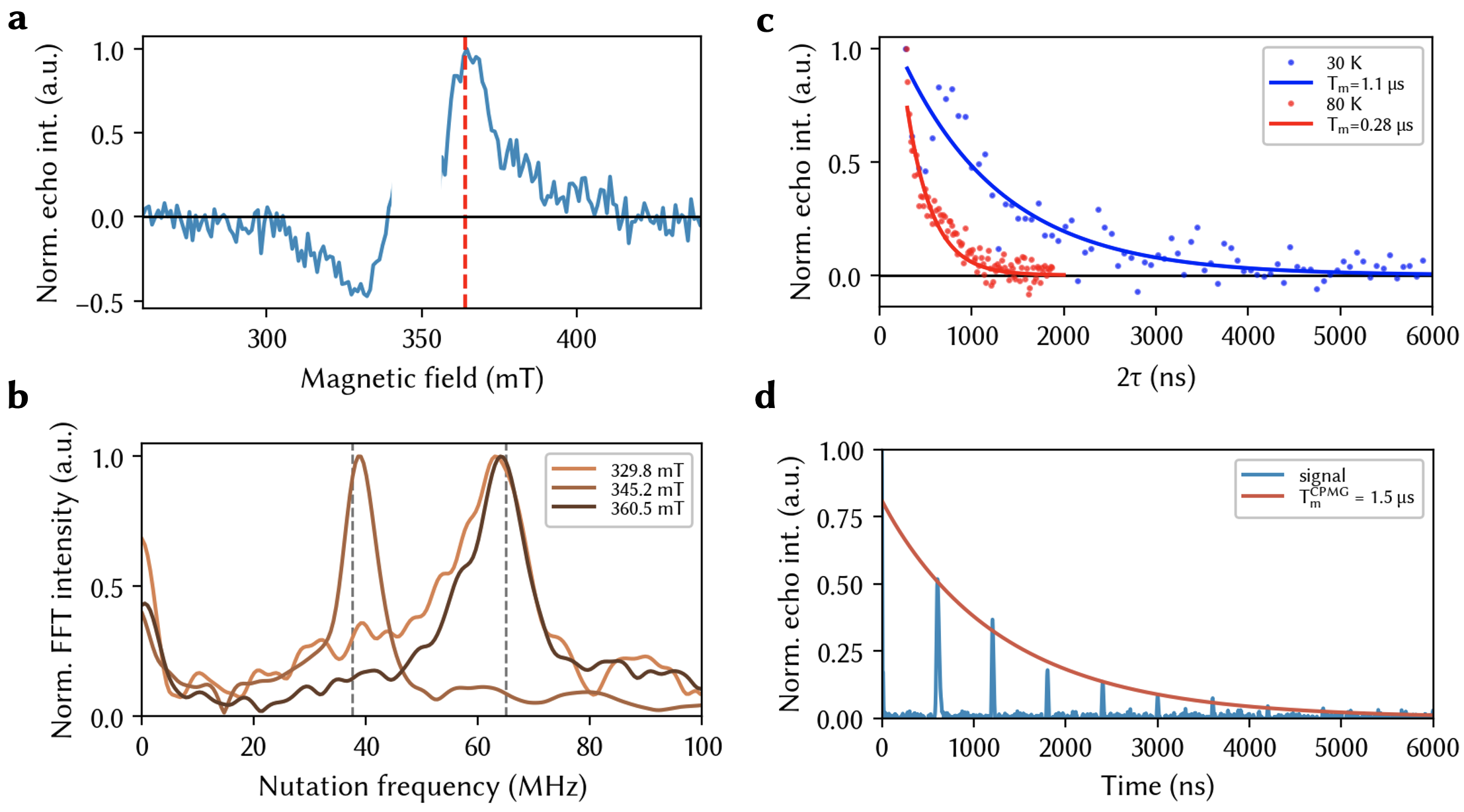


**Fig. S30** Pulsed X-band ESR on 100 µᴍ DMAC-TEMPO in d8-toluene solution performed after 470 nm 1 mJ excitation. a) Echo-detected field sweep at 30 K. b) Nutation frequency at key field positions at 30 K. Dashed lines are relative to the doublet nutation frequency measured in the dark. c) Coherence time measurements as a function of temperature at quartet field position indicated with the dashed line in panel a. d) Dynamic decoupling sequence showing effective quartet coherence time extended to 1.5 µs at 30 K. CPMG pulse sequence used.^[12]^


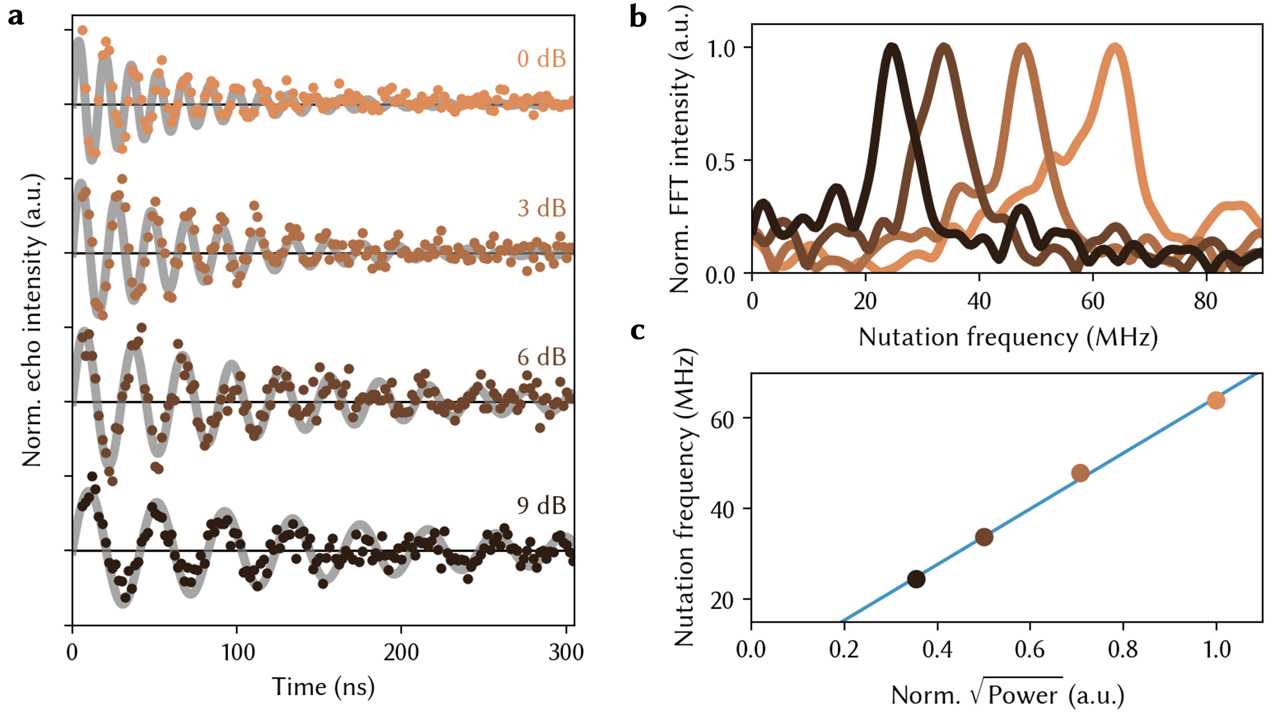


**Fig. S31** Transient Rabi oscillations as a function of microwave drive attenuation on 100 µᴍ DMAC-TEMPO in d8-toluene solution at 30 K. Performed after 470 nm 1 mJ excitation at a quartet field position, *g* = 1.919, at X-band. a) Time domain data (dots) and fits (grey solid lines). b) Fourier transforms. c) Linear dependence confirms the quartet spin system can be placed at an arbitrary superposition.

# System comparison

**Table S3** Examples of light-emitting materials with access to electron spin manipulation

| Platform | Ref. | λ_PL_ (nm) | *S* | Example compound | Temp. (K) | τ_2_  (µs) | PLQE  (%) | Ω_M_ |
| --- | --- | --- | --- | --- | --- | --- | --- | --- |
| Organometallics | ^[13]^ | 1000-1100 | 1 | Cr(o-tolyl)_4_ | 5 | 0.6 | 10 | 9 |
|  |  |  |  |  | - | - | - | - |
| Organic dyes | ^[14]^ | 600-650 | 1 | Pentacene  in o-terphenyl | - | - | - | - |
|  |  |  |  |  | 293 | 1.2 | 7 | 22 |
| Trityl radical chromophores | ^[15]^ | 700-750 | 3/2, 2 | TTM-1Cz-An | 80 | 1.5 | 12 | 70 |
|  |  |  |  |  | 295 | 0.2 | 32 | - |
| Radical TADF | *This work* | 530-710 | 3/2 | DMAC-TEMPO | 30 | 1.1 | 5 | 90 |
|  |  |  |  |  | 295 | - | 10 | - |

*S* – electron spin of the wavefunction manipulated with microwaves

τ_2_ – spin coherence time under standard Hahn echo pulse sequence

Ω_M_ – quantum fidelity, the maximum possible number of spin manipulations under employed conditions

# Quantum chemical calculations

We model the vertical and adiabatic transition energies of our closed-shell materials in toluene (𝜖 = 2.38). Using hole-particle analysis, we identify the first excited singlet, ^1^CT, and two lowest-lying triplet states, ^3^CT and ^3^LE_NAI_, for each system. Excited-state optimization of each state allowed us to construct the adiabatic state diagrams for closed-shell molecules (Figure S25). The excited state geometries of ^1^CT and ^3^CT are almost identical, accompanied by a similar configuration composition of their respective transitions, with negligible energy differences of about 0.02 eV (Tables S4-6). The most substantial structural changes are present for the relaxed ^3^LE_NAI_ state, stabilised by reducing the twisting angle between the donor and NAI moieties (Table S7).

The adiabatic state diagrams of Cz-Cy and DMAC-Cy are similar, with ^3^LE_NAI_ remaining the lowest-lying excited state along the curve. The main difference is the energy gap between ^3^LE_NAI_ and ^1,3^CT states, which is consistently smaller for the DMAC-Cy molecule. We also note a substantial degree of CT and LE mixing in Cz-Cy transitions compared to the rest of the series, as indicated by the electron-hole overlap integrals (*S_r_*) describing the states’ character.

In Cz-Cy and DMAC-Cy, ^1^CT and ^3^CT states are almost degenerate in their relaxed geometries, and ^3^LE_NAI_ lies below, preserving the trend observed in vertical excitations. In contrast, PXZ-Cy has all three excited states almost iso-energetic at the same geometries, with ^3^LE_NAI_ above the CT states at the ^1^CT minimum. This reveals that the energetic ordering the triplet states depends on molecular conformation for PXZ-Cy.

| 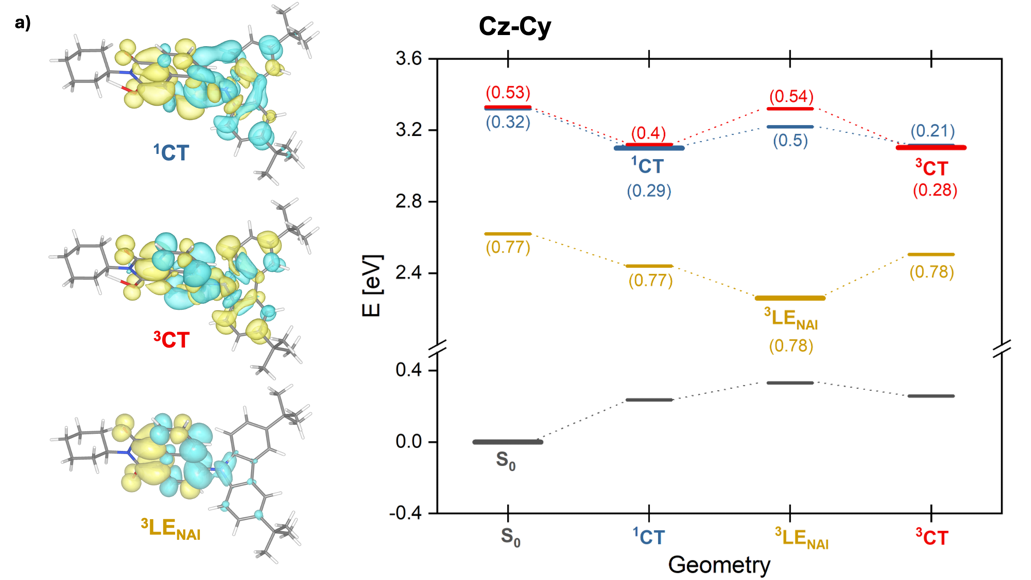 |
| --- |
| 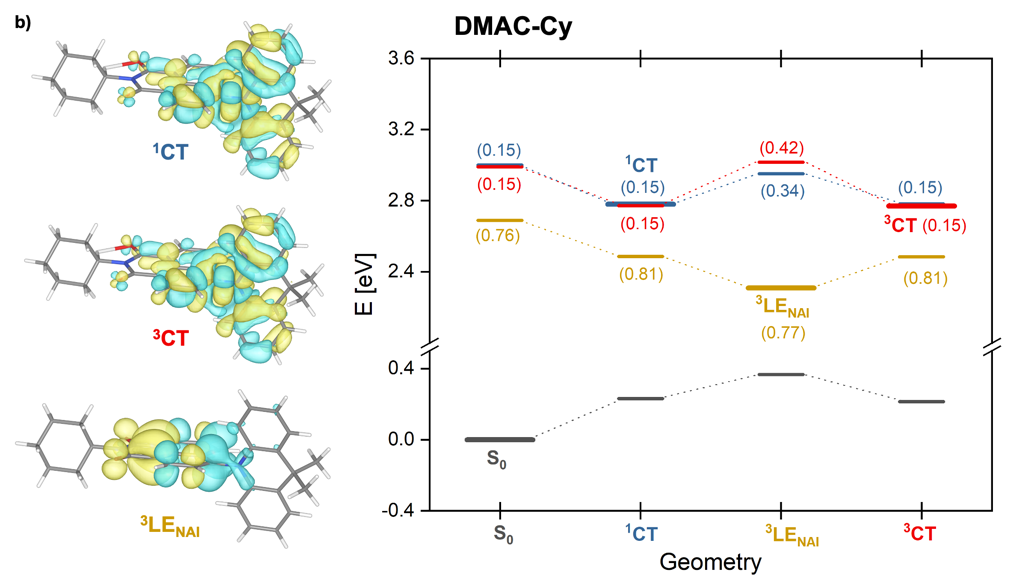 |
| 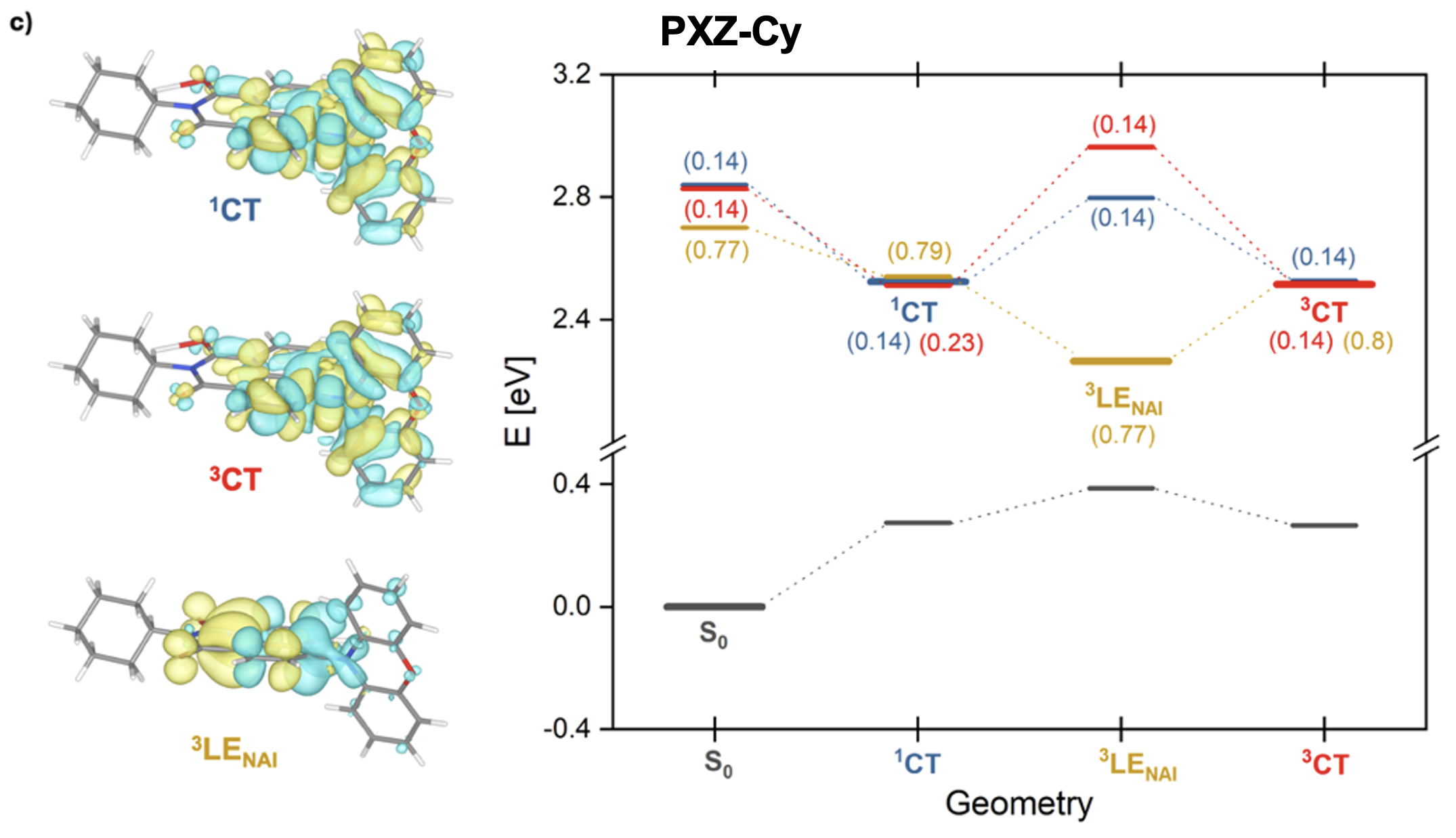 |

**Fig. S32** *Left*: adiabatic transition densities of ^1^CT (blue), ^3^LE_NAI_ (yellow) and ^3^CT (red) states in toluene (ε=2.38). *Right:* state diagrams for a) Cz-Cy, b) DMAC-Cy and c) PXZ-Cy molecules. S_0_ panel represents vertical excitation energies, and other panels refer to excitations from the excited state geometries denoted on the abscissa. Electron-hole overlap integrals (*S_r_*) for each excited state are given in parentheses.

**Table S4** Configuration, electron-hole overlap (*S*_r_) and excitation energy (*E*) for vertical (S_0_ geometry) and adiabatic excitations (^1^CT, ^3^LE_NAI_, ^3^CT geometry) of Cz-Cy molecule in toluene.

| Cz-Cy | | | | |
| --- | --- | --- | --- | --- |
|  | *State* | Configuration^a^ | *S_r_* | *E* [eV] |
| S_0_ geometry | **S_0_** |  |  | 0.00 |
|  | ^1^CT | 93% *h*_0_ → *l*_0_ | 0.32 | 3.32 |
|  | ^3^LE_NAI_ | 23% *h*_0_ → *l*_0_ 64% *h_-2_* → *l*_0_ | 0.77 | 2.62 |
|  | ^3^CT | 58% *h*_0_ → *l*_0_ 19% *h_-2_* → *l*_0_ | 0.53 | 3.33 |
| ^1^CT geometry | S_0_ |  |  | 0.24 |
|  | **^1^CT** | 95% *h*_0_ → *l*_0_ | 0.29 | 3.10 |
|  | ^3^LE_NAI_ | 22% *h*_0_ → *l*_0_ 69% *h_-2_* → *l*_0_ | 0.77 | 2.44 |
|  | ^3^CT | 68% *h*_0_ → *l*_0_ 21% *h_-2_* → *l*_0_ | 0.40 | 3.12 |
| ^3^LE_NAI_ geometry | S_0_ |  |  | 0.33 |
|  | ^1^CT | 94% *h*_0_ → *l*_0_ | 0.50 | 3.22 |
|  | **^3^LE_NAI_** | 52% *h*_0_ → *l*_0_ 42% *h_-2_* → *l*_0_ | 0.78 | 2.26 |
|  | ^3^CT | 44% *h_-2_* → *l*_0_ 34% *h*_0_ → *l*_0_ | 0.54 | 3.32 |
| ^3^CT geometry | S_0_ |  |  | 0.26 |
|  | ^1^CT | 95% *h*_0_ → *l*_0_ | 0.21 | 3.12 |
|  | ^3^LE_NAI_ | 80% *h_-2_* → *l*_0_ | 0.78 | 2.51 |
|  | **^3^CT** | 82% *h*_0_ → *l*_0_ | 0.28 | 3.10 |
| ^a^Dominant configurations for each state. *h*_x_ → *l_y_* refers to HOMO-x → LUMO-y transition. Relevant orbitals are shown in Fig. S33 | | | | |

**Table S5** Configuration, electron-hole overlap (*S*_r_) and excitation energy (*E*) for vertical (S_0_ geometry) and adiabatic excitations (^1^CT, ^3^LE_NAI_, ^3^CT geometry) of DMAC-Cy molecule in toluene.

| DMAC-Cy | | | | |
| --- | --- | --- | --- | --- |
|  | *State* | Configuration^a^ | *S_r_* | *E* [eV] |
| S_0_ geometry | **S_0_** |  |  | 0.00 |
|  | ^1^CT | 94% *h*_0_ → *l*_0_ | 0.15 | 3.00 |
|  | ^3^LE_NAI_ | 88% *h_-1_* → *l*_0_ | 0.76 | 2.69 |
|  | ^3^CT | 94% *h*_0_ → *l*_0_ | 0.15 | 2.99 |
| ^1^CT geometry | S_0_ |  |  | 0.23 |
|  | **^1^CT** | 95% *h*_0_ → *l*_0_ | 0.15 | 2.78 |
|  | ^3^LE_NAI_ | 91% *h_-1_* → *l*_0_ | 0.81 | 2.49 |
|  | ^3^CT | 95% *h*_0_ → *l*_0_ | 0.15 | 2.77 |
| ^3^LE_NAI_ geometry | S_0_ |  |  | 0.37 |
|  | ^1^CT | 95% *h_-1_* → *l*_0_ | 0.34 | 2.95 |
|  | **^3^LE_NAI_** | 60% *h_-1_* → *l*_0_ 33% *h*_0_ → *l*_0_ | 0.77 | 2.31 |
|  | ^3^CT | 59% *h*_0_ → *l*_0_ 32% *h_-1_* → *l*_0_ | 0.42 | 3.02 |
| ^3^CT geometry | S_0_ |  |  | 0.21 |
|  | ^1^CT | 95% *h*_0_ → *l*_0_ | 0.15 | 2.78 |
|  | ^3^LE_NAI_ | 91% *h_-1_* → *l*_0_ | 0.81 | 2.48 |
|  | **^3^CT** | 95% *h*_0_ → *l*_0_ | 0.15 | 2.77 |
| ^a^Dominant configurations for each state. *h*_x_ → *l_y_* refers to HOMO-x → LUMO-y transition. Relevant orbitals are shown in Fig. S33 | | | | |

**Table S6** Configuration, electron-hole overlap (*S*_r_) and excitation energy (*E*) for vertical (S_0_ geometry) and adiabatic excitations (^1^CT, ^3^LE_NAI_, ^3^CT geometry) of PXZ-Cy molecule in toluene.

| PXZ-Cy | | | | |
| --- | --- | --- | --- | --- |
|  | *State* | Configuration^a^ | *S_r_* | *E* [eV] |
| S_0_ geometry | **S_0_** |  |  | 0.00 |
|  | ^1^CT | 94% *h*_0_ → *l*_0_ | 0.14 | 2.84 |
|  | ^3^LE_NAI_ | 89% *h_-1_* → *l*_0_ | 0.77 | 2.70 |
|  | ^3^CT | 93% *h*_0_ → *l*_0_ | 0.14 | 2.83 |
| ^1^CT geometry | S_0_ |  |  | 0.27 |
|  | **^1^CT** | 95% *h*_0_ → *l*_0_ | 0.14 | 2.52 |
|  | ^3^LE_NAI_ | 92% *h*_0_ → *l*_0_ | 0.23 | 2.51 |
|  | ^3^CT | 89% *h_-1_* → *l*_0_ | 0.79 | 2.54 |
| ^3^LE_NAI_ geometry | S_0_ |  |  | 0.39 |
|  | ^1^CT | 94% *h*_0_ → *l*_0_ | 0.34 | 2.80 |
|  | **^3^LE_NAI_** | 51% *h*_0_ → *l*_0_ 41% *h_-1_* → *l*_0_ | 0.70 | 2.26 |
|  | ^3^CT | 43% *h_-1_* → *l*_0_ 40% *h*_0_ → *l*_0_ | 0.55 | 2.96 |
| ^3^CT geometry | S_0_ |  |  | 0.27 |
|  | ^1^CT | 95% *h*_0_ → *l*_0_ | 0.14 | 2.53 |
|  | ^3^LE_NAI_ | 91% *h_-1_* → *l*_0_ | 0.80 | 2.51 |
|  | **^3^CT** | 94% *h*_0_ → *l*_0_ | 0.14 | 2.52 |
| ^a^Dominant configurations for each state. *h*_x_ → *l_y_* refers to HOMO-x → LUMO-y transition. Relevant orbitals are shown in Fig. S33 | | | | |

| 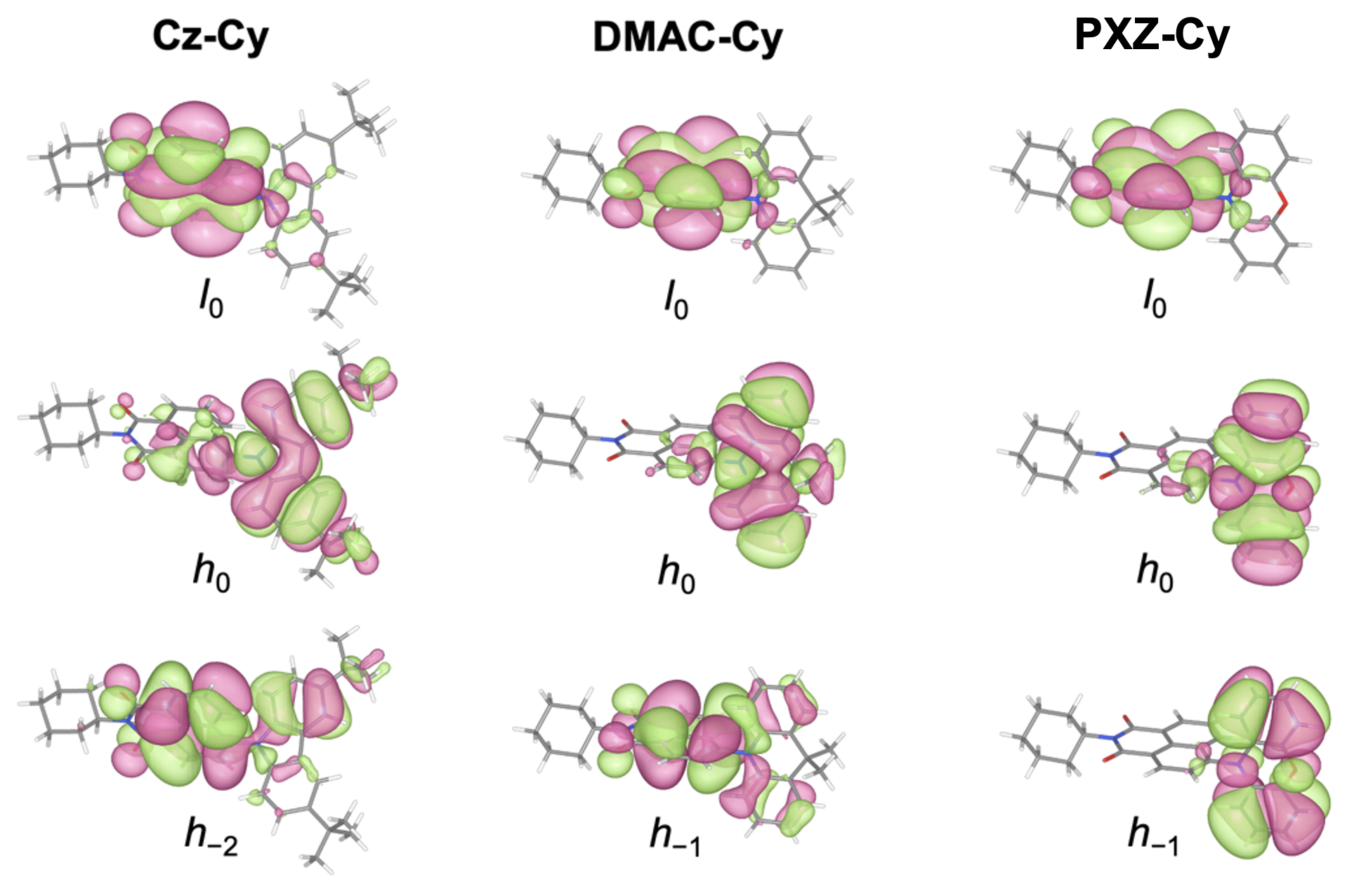 |
| --- |

**Fig. S33** Molecular orbitals of Cz-Cy, DMAC-Cy and PXZ-Cy relevant for low-lying transitions described in Tables X-Y. *h* and *l* refer to occupied and virtual frontier orbitals, respectively.

**Table S7** Torsion angles between NAI and electron donating moieties (Cz, DMAC and PXZ) of studied closed-shell TADF materials for each calculated adiabatic state geometry. Angles are presented in degrees.

| **State** | **Cz-Cy** | **DMAC-Cy** | **PXZ-Cy** |
| --- | --- | --- | --- |
| **S_0_** | 62.4 | 88.4 | 83.2 |
| **^1^CT** | 67.8 | 89.7 | 89.6 |
| **^3^LE_NAI_** | 57.3 | 66.9 | 59.4 |
| **^3^CT** | 74.0 | 89.6 | 89.4 |

# NMR spectra


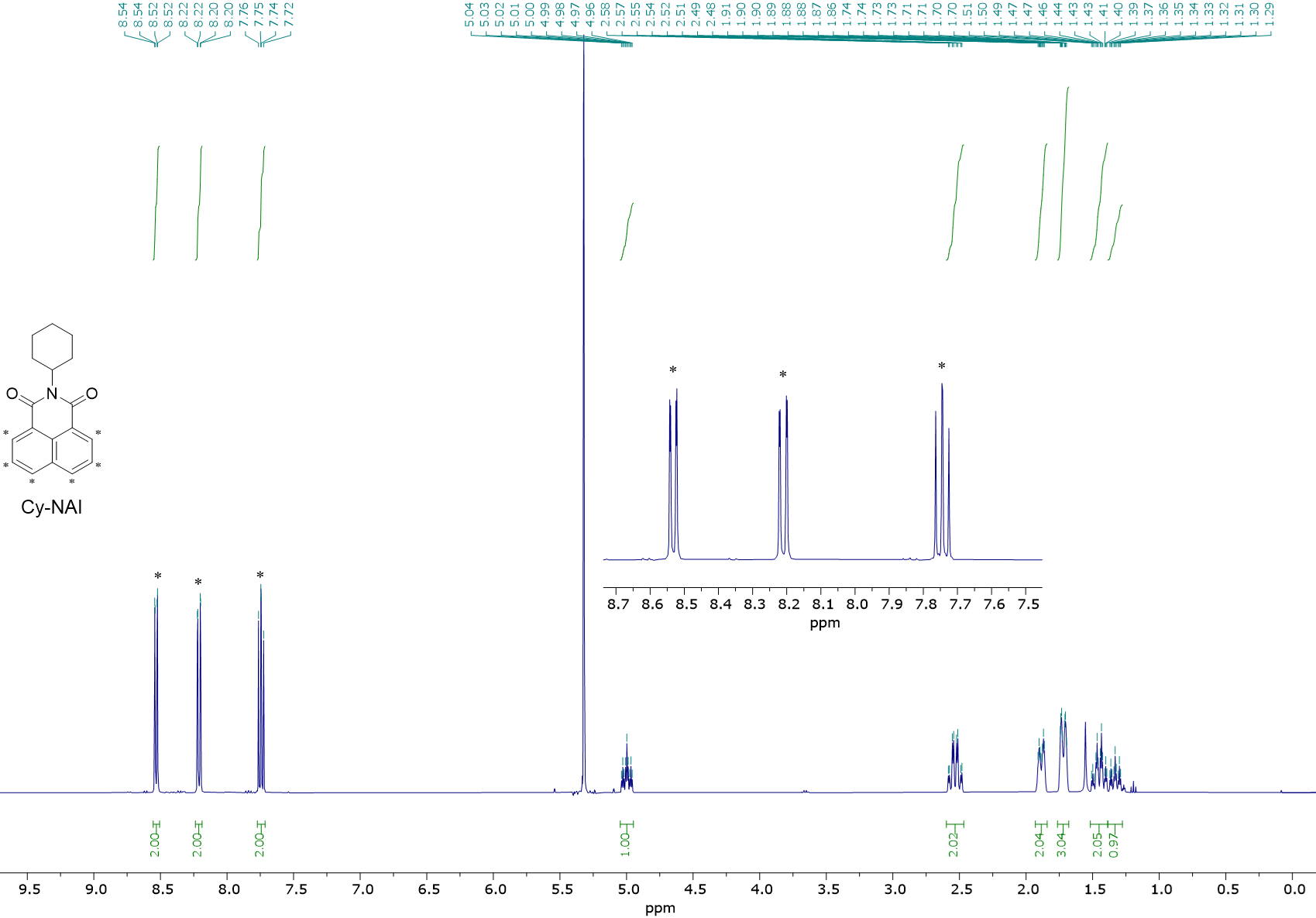


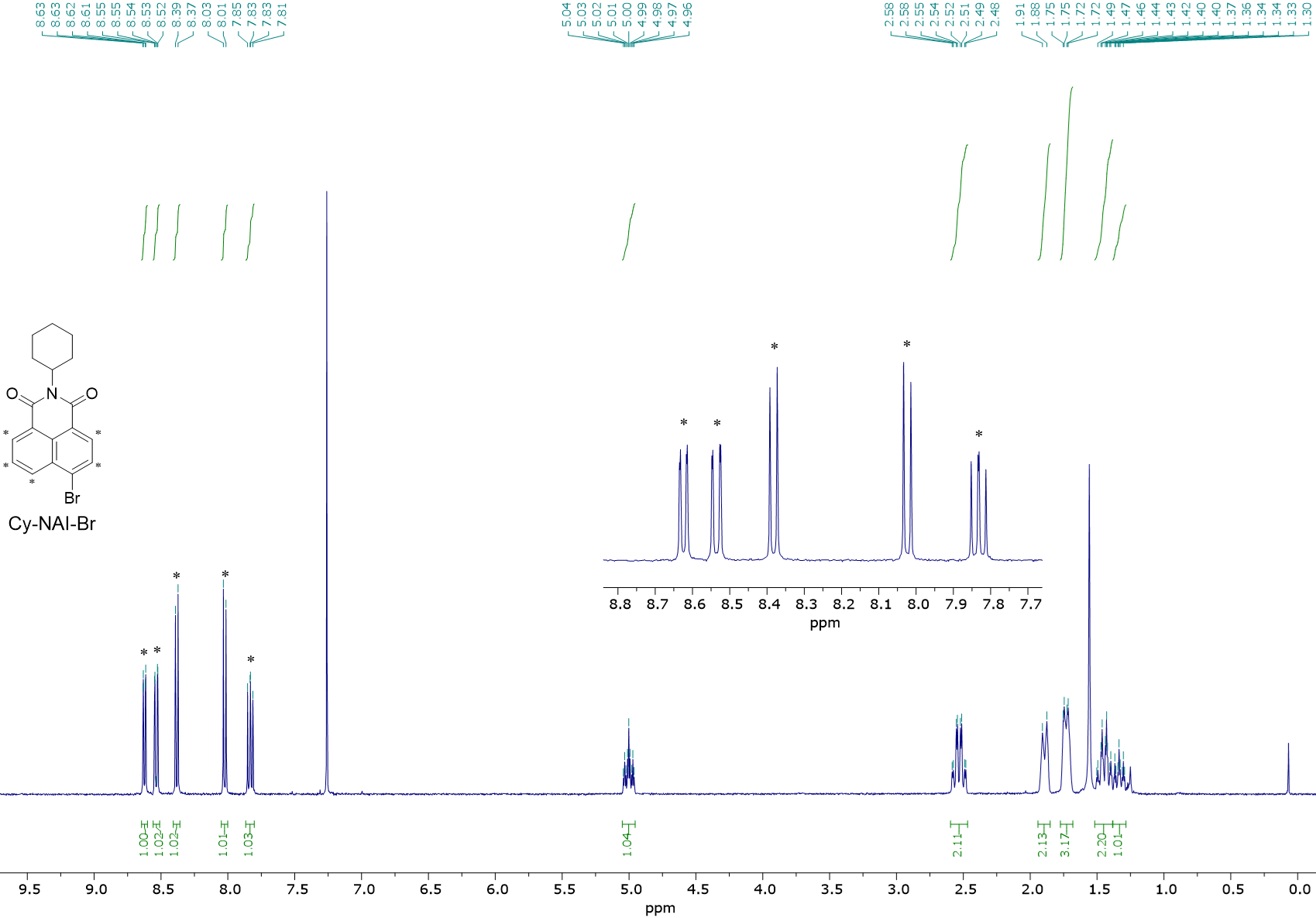


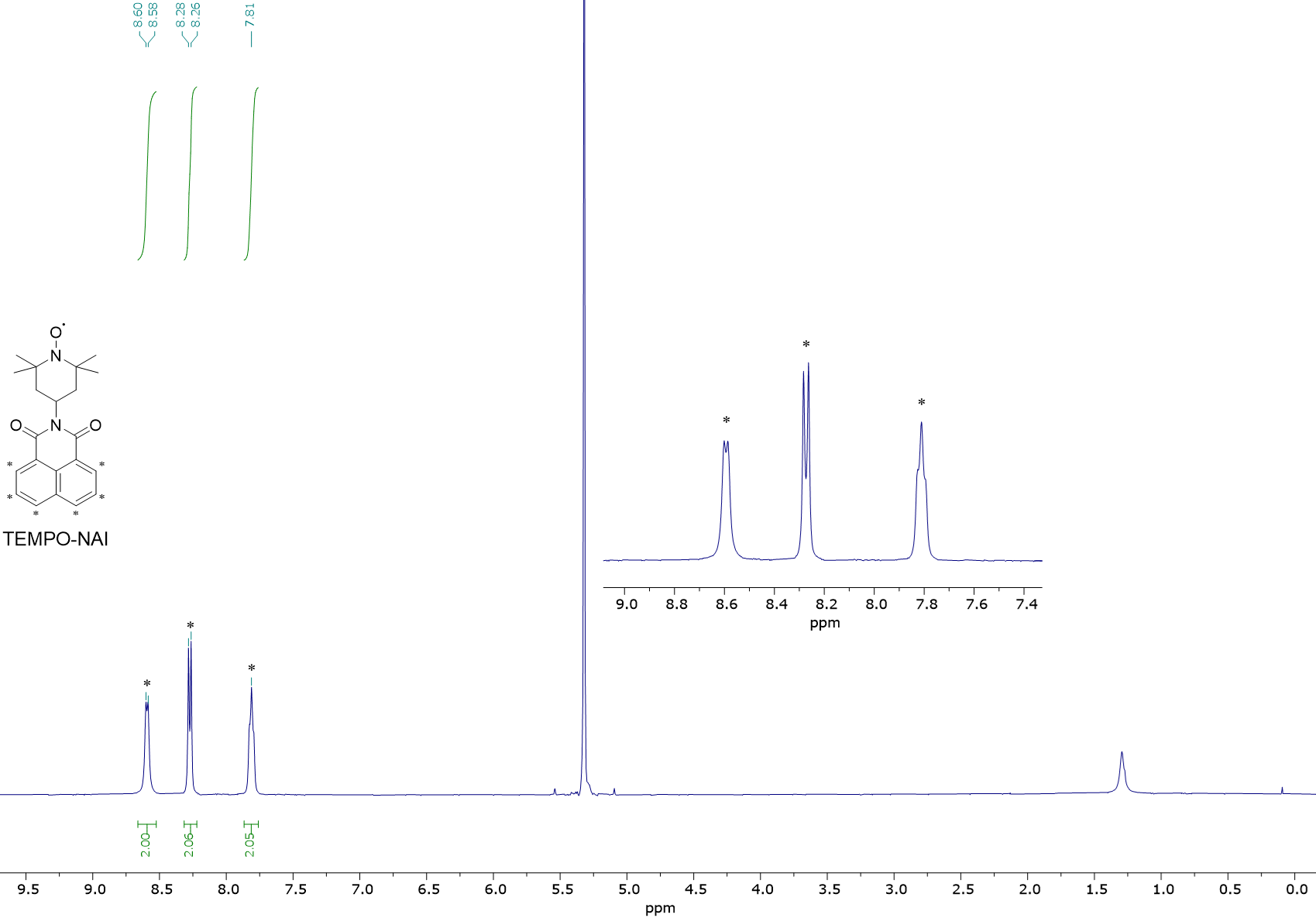


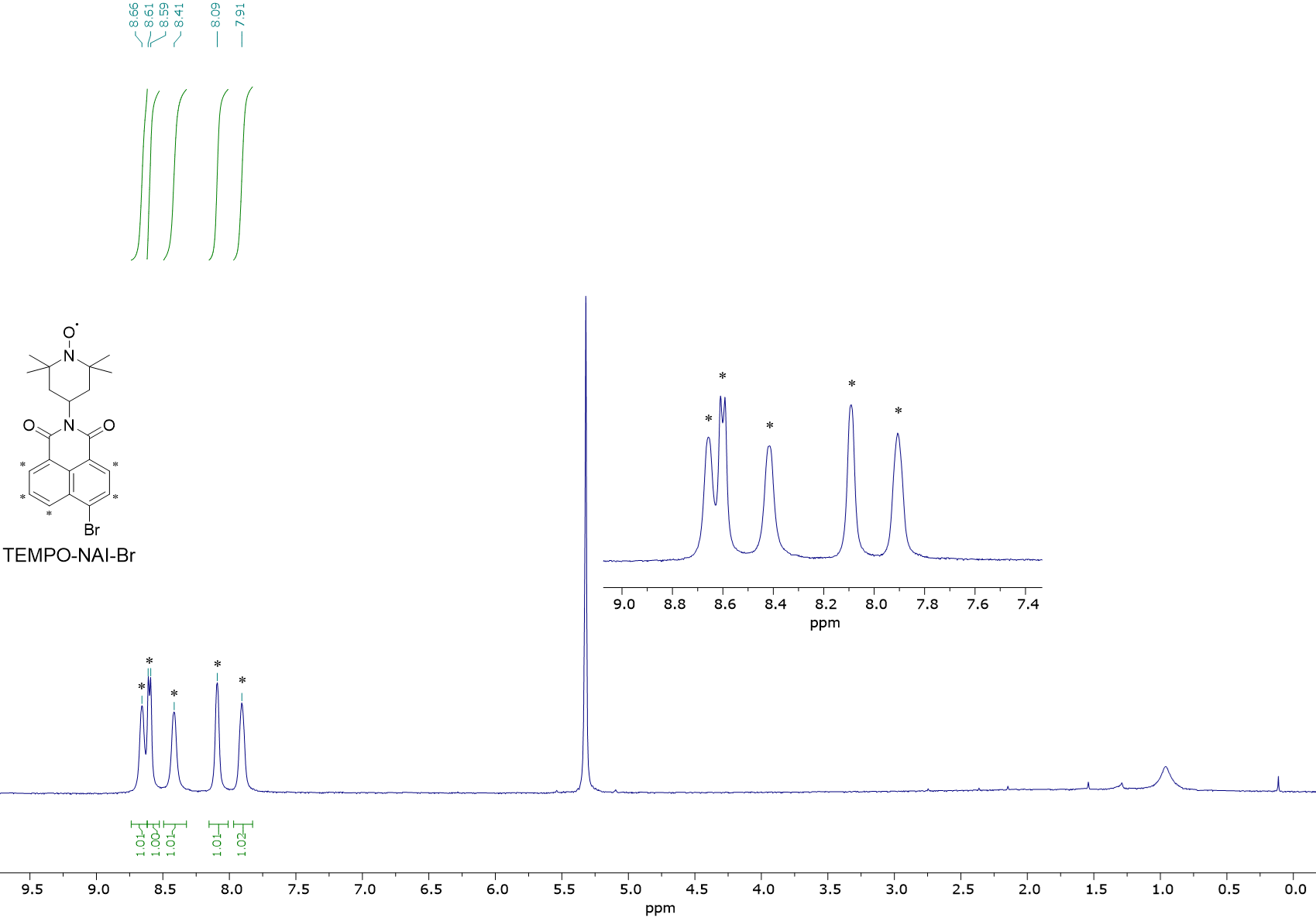


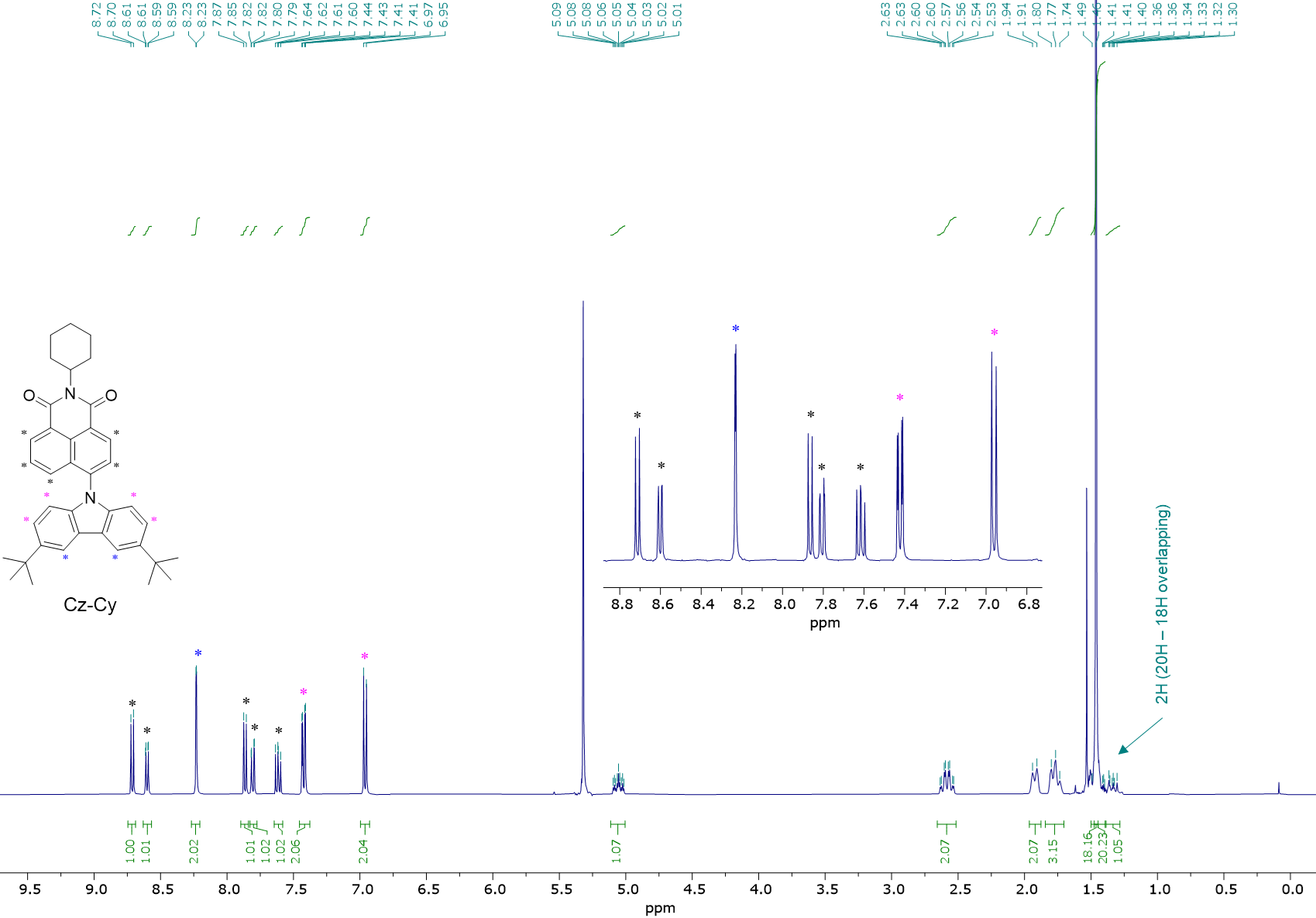


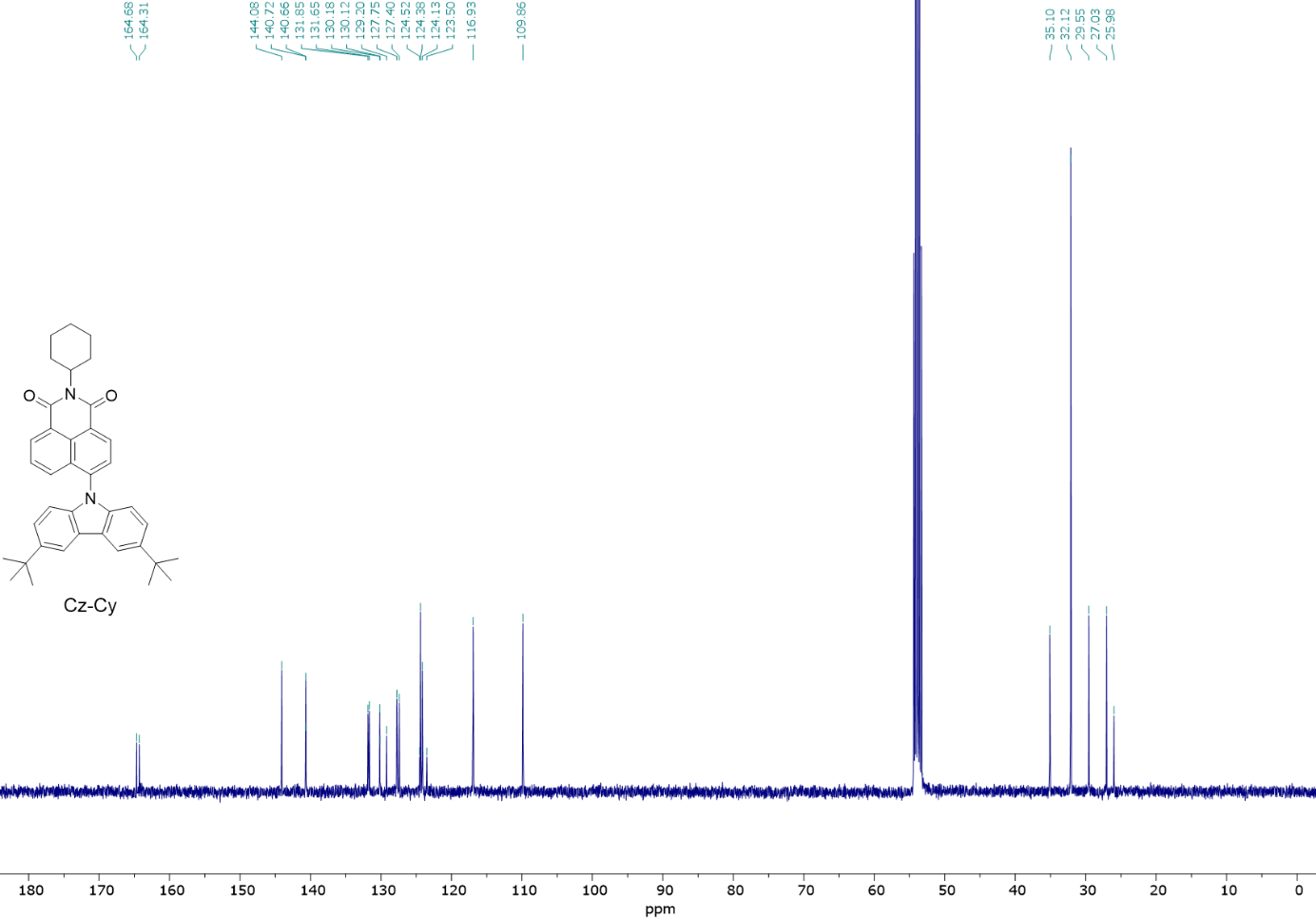


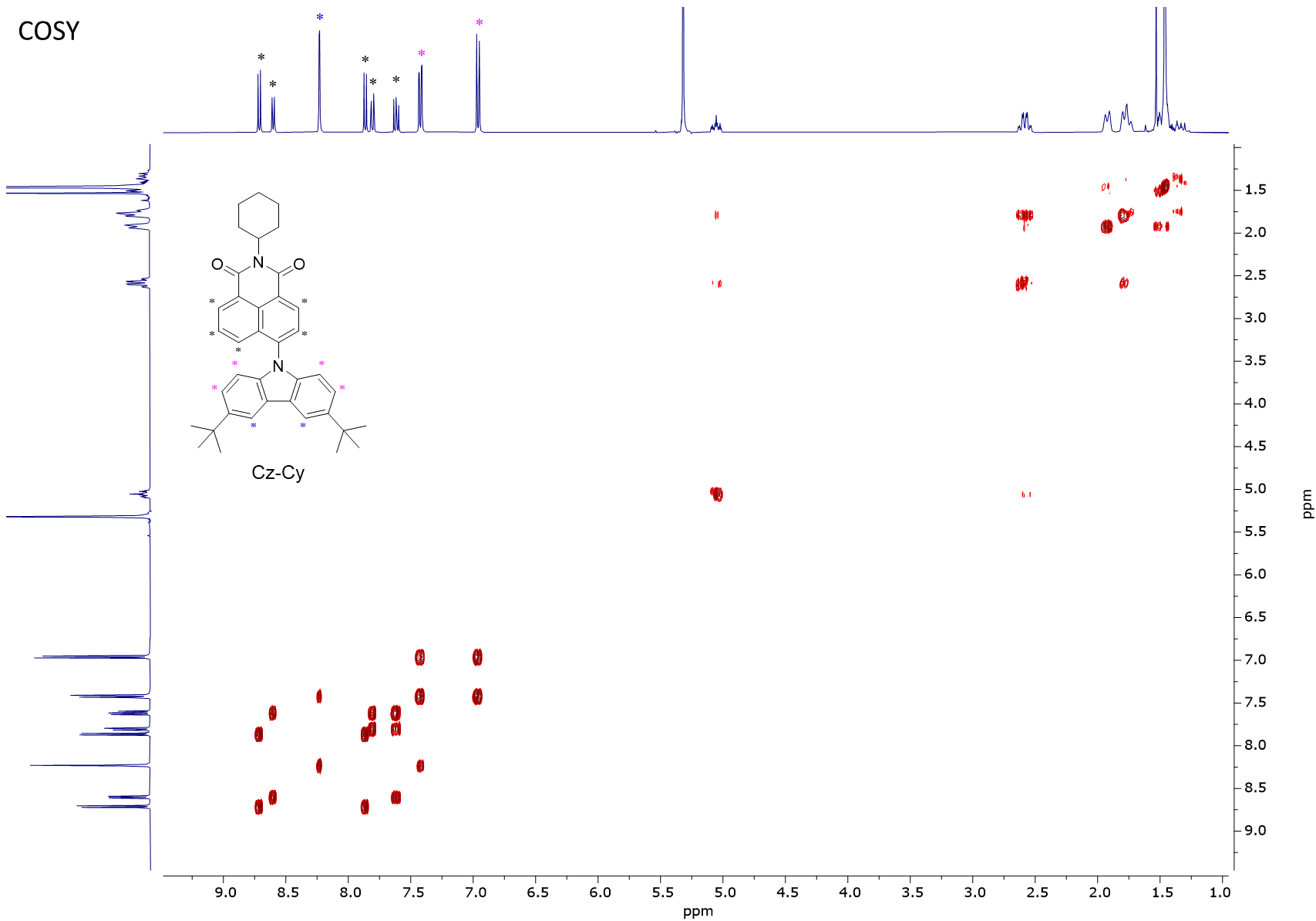


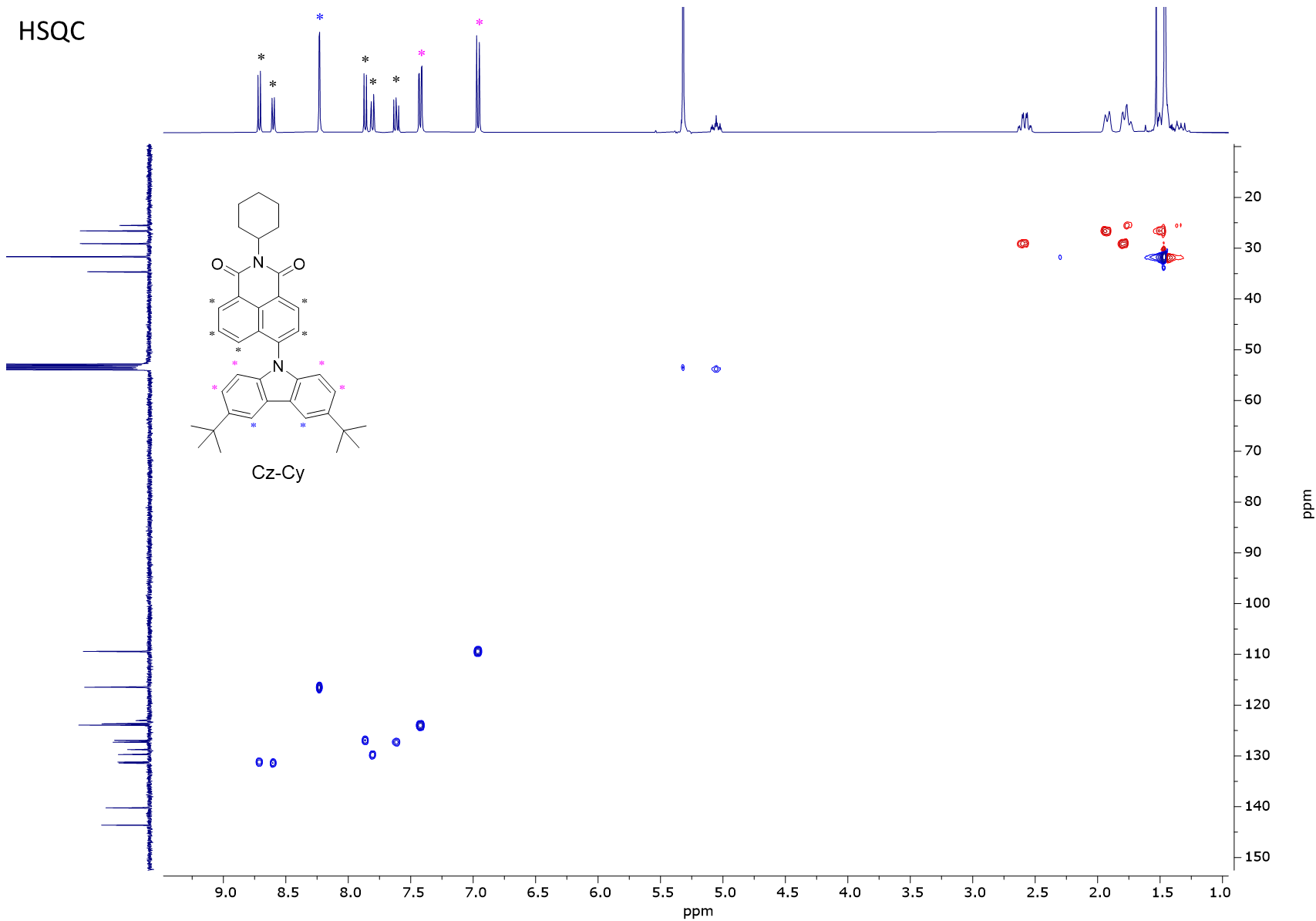


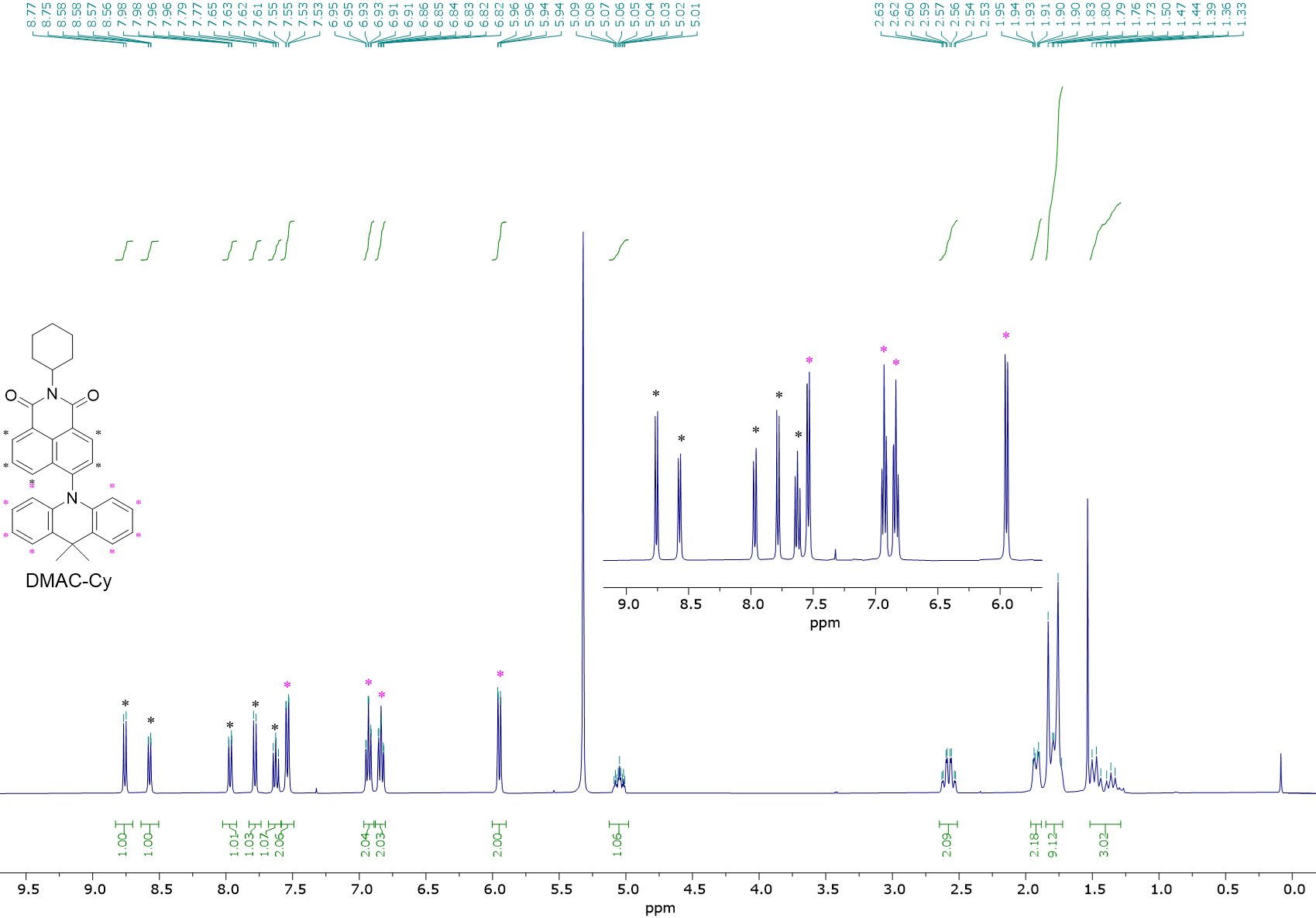


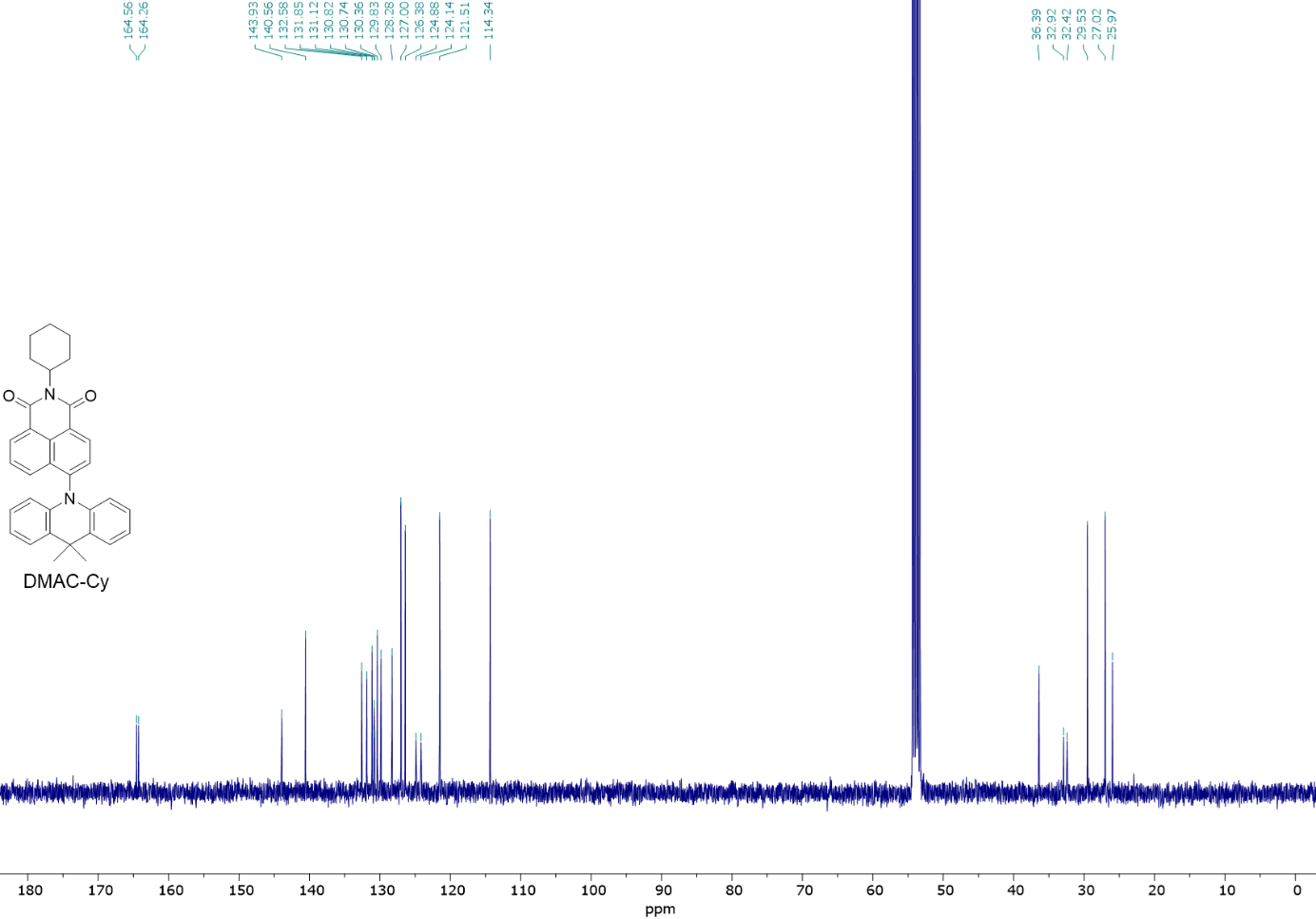


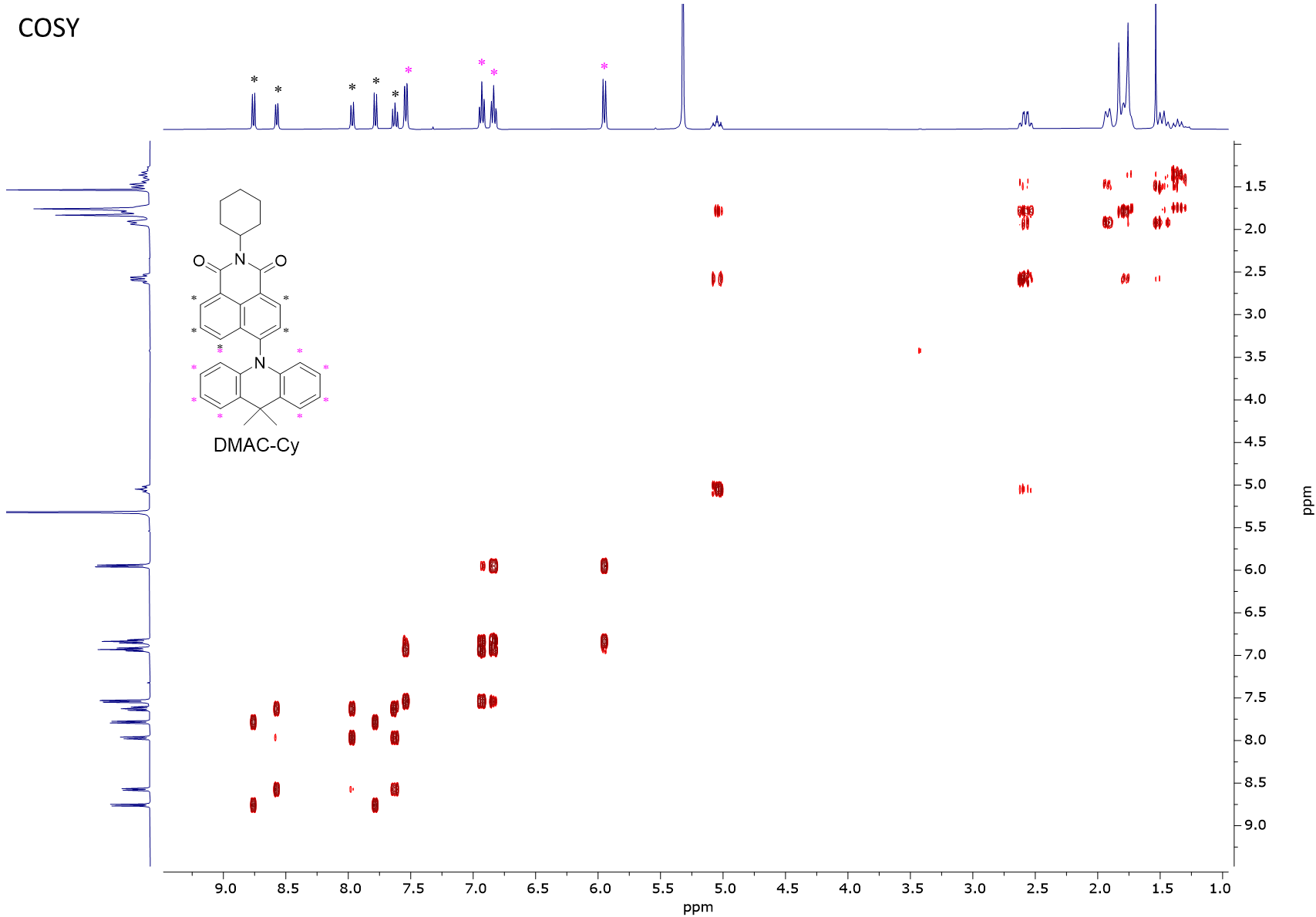


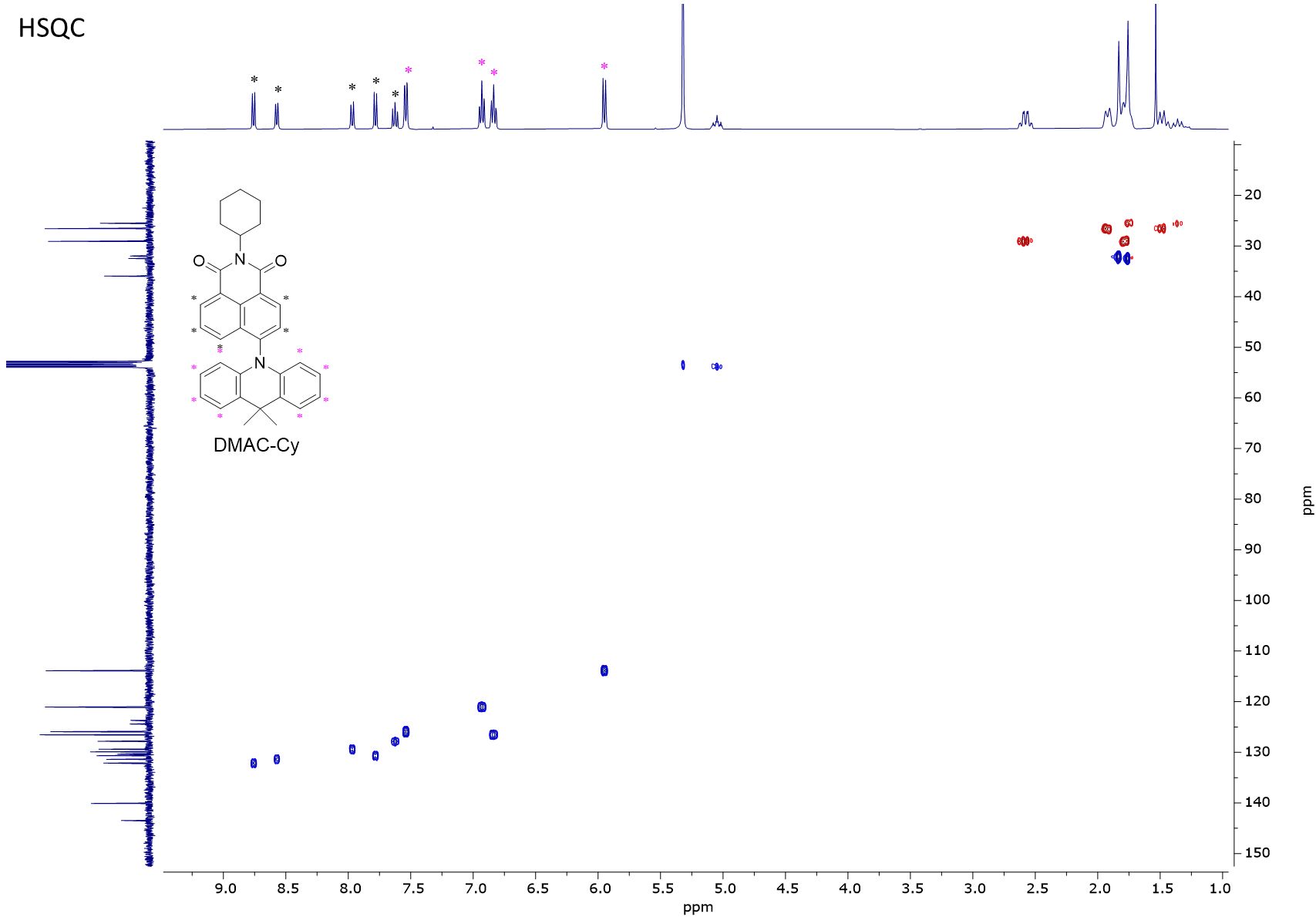


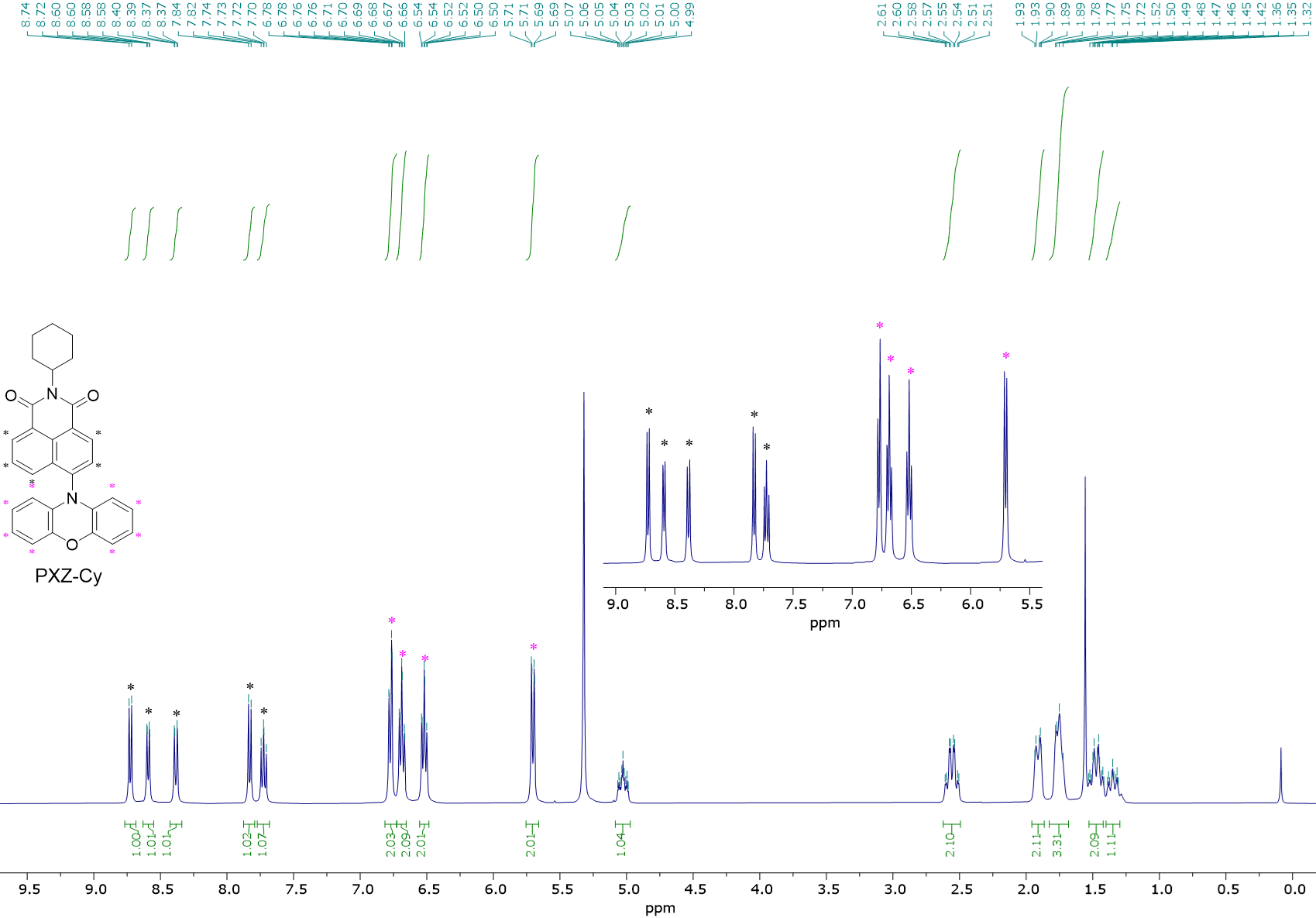


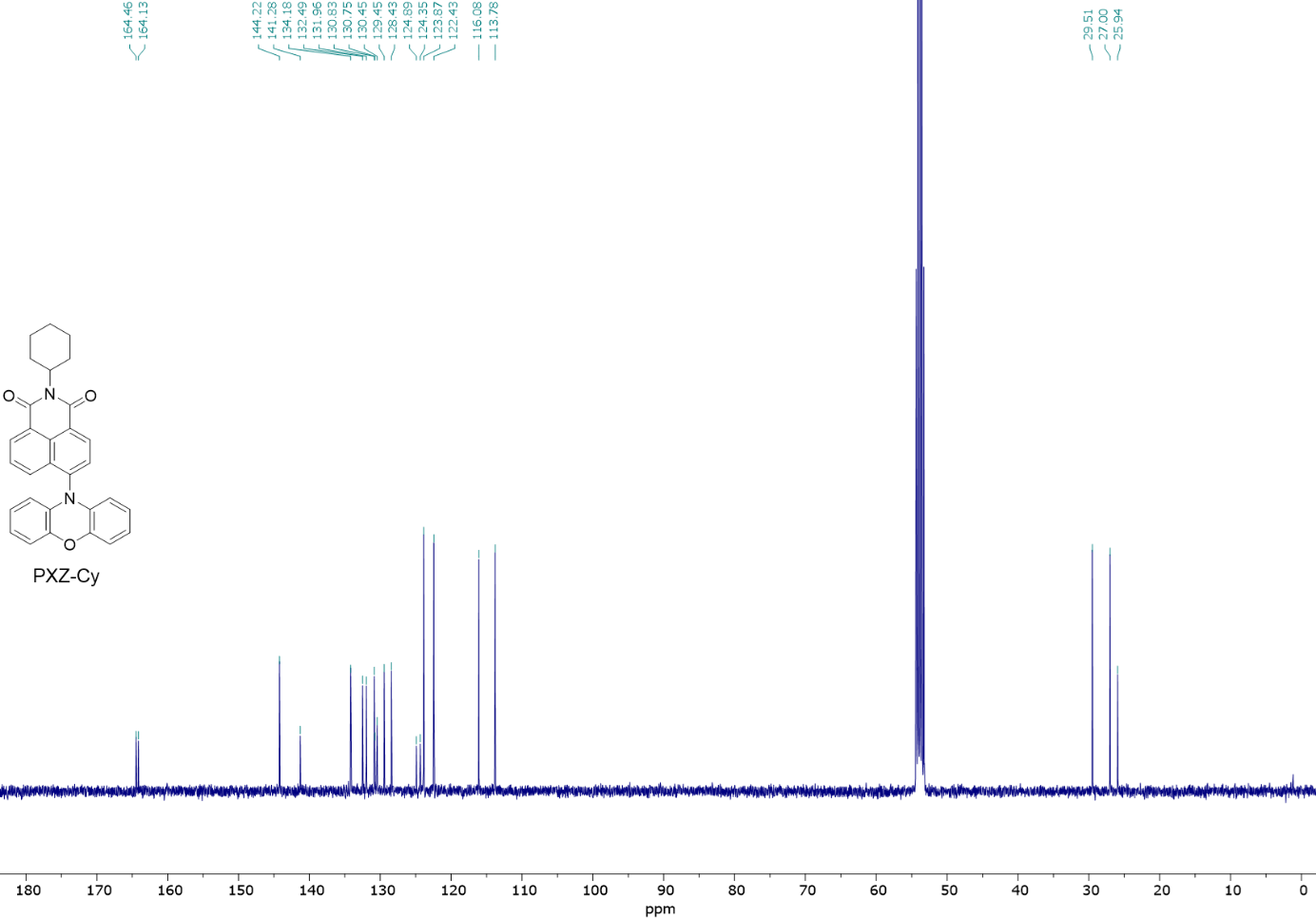


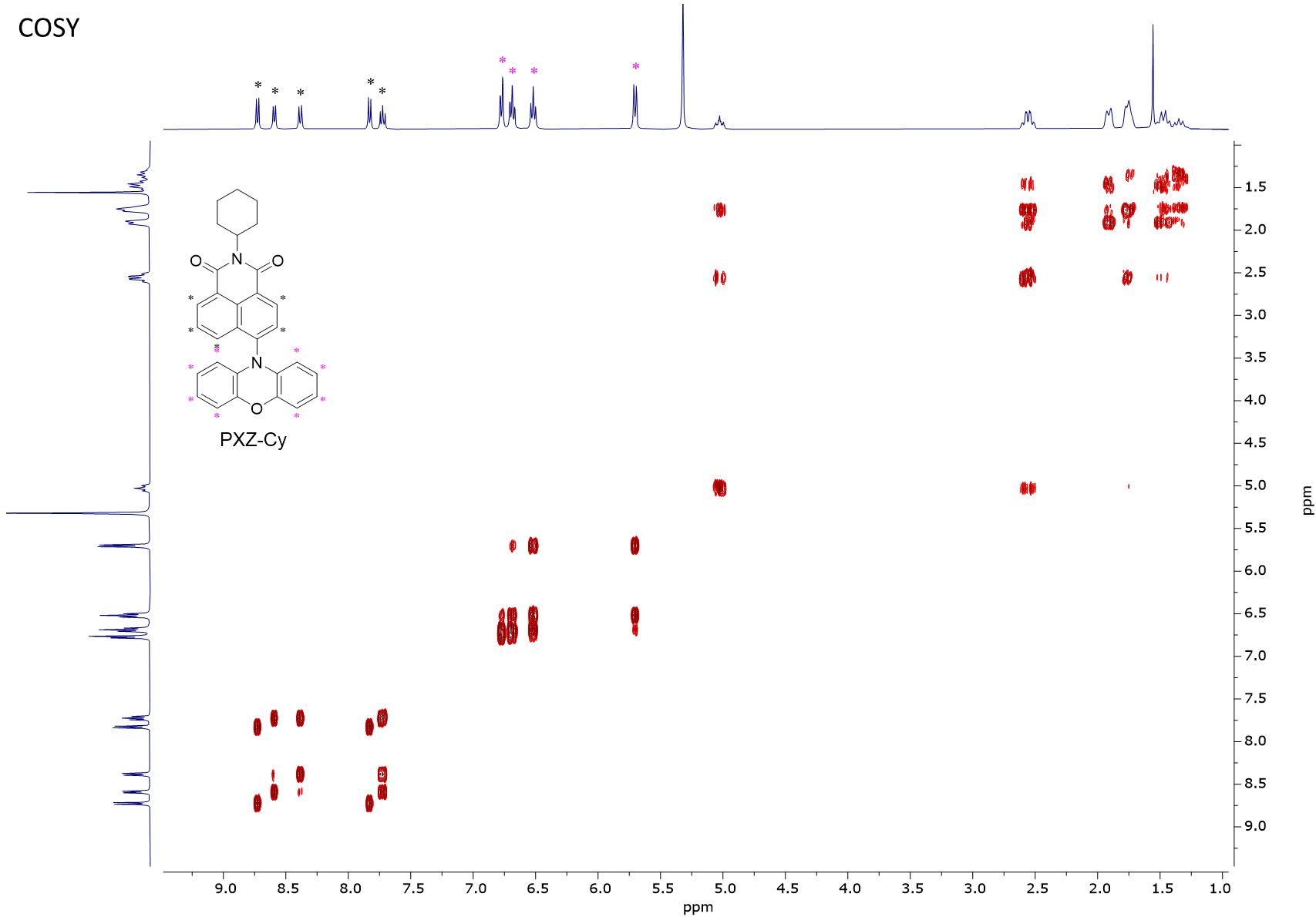


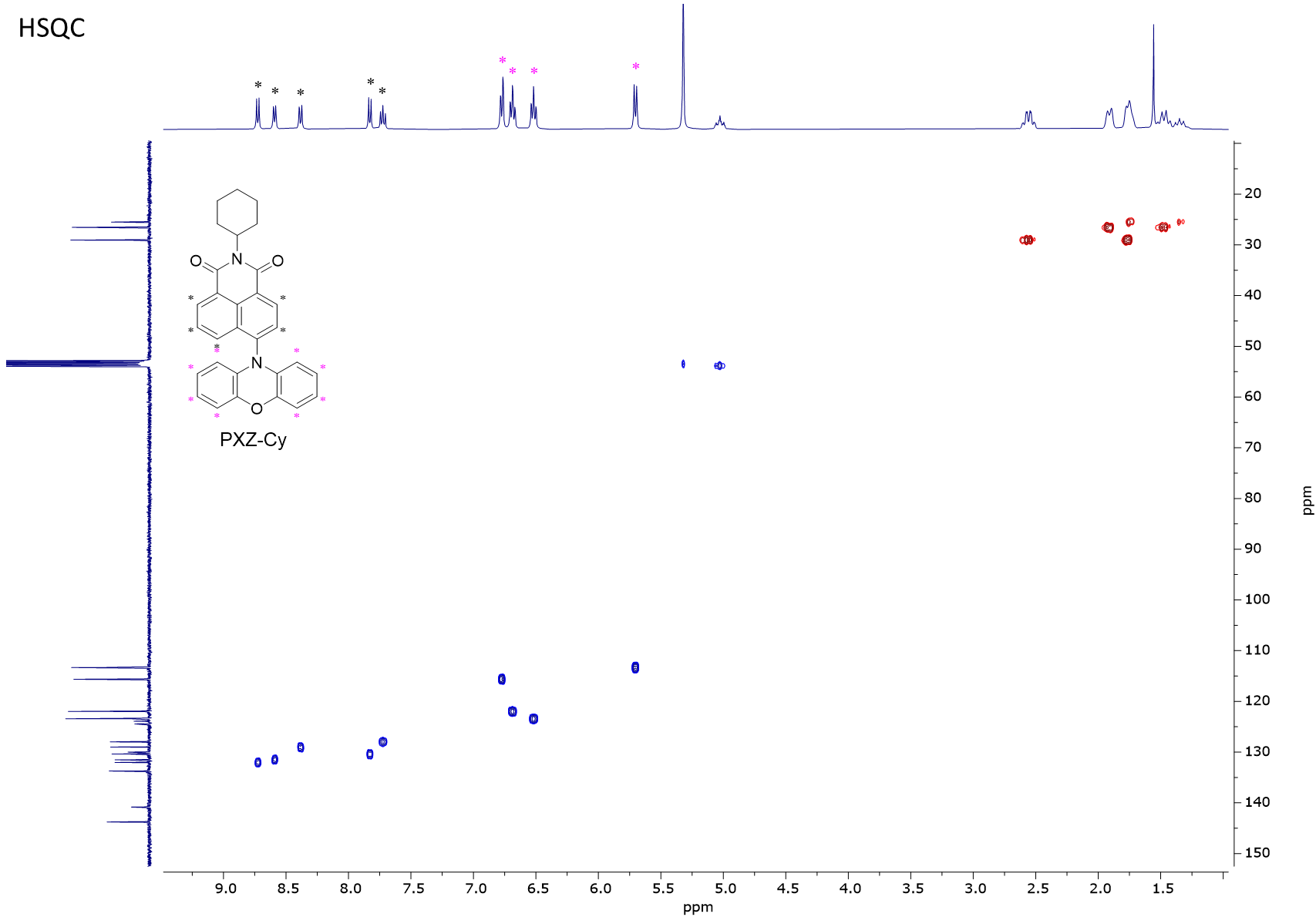


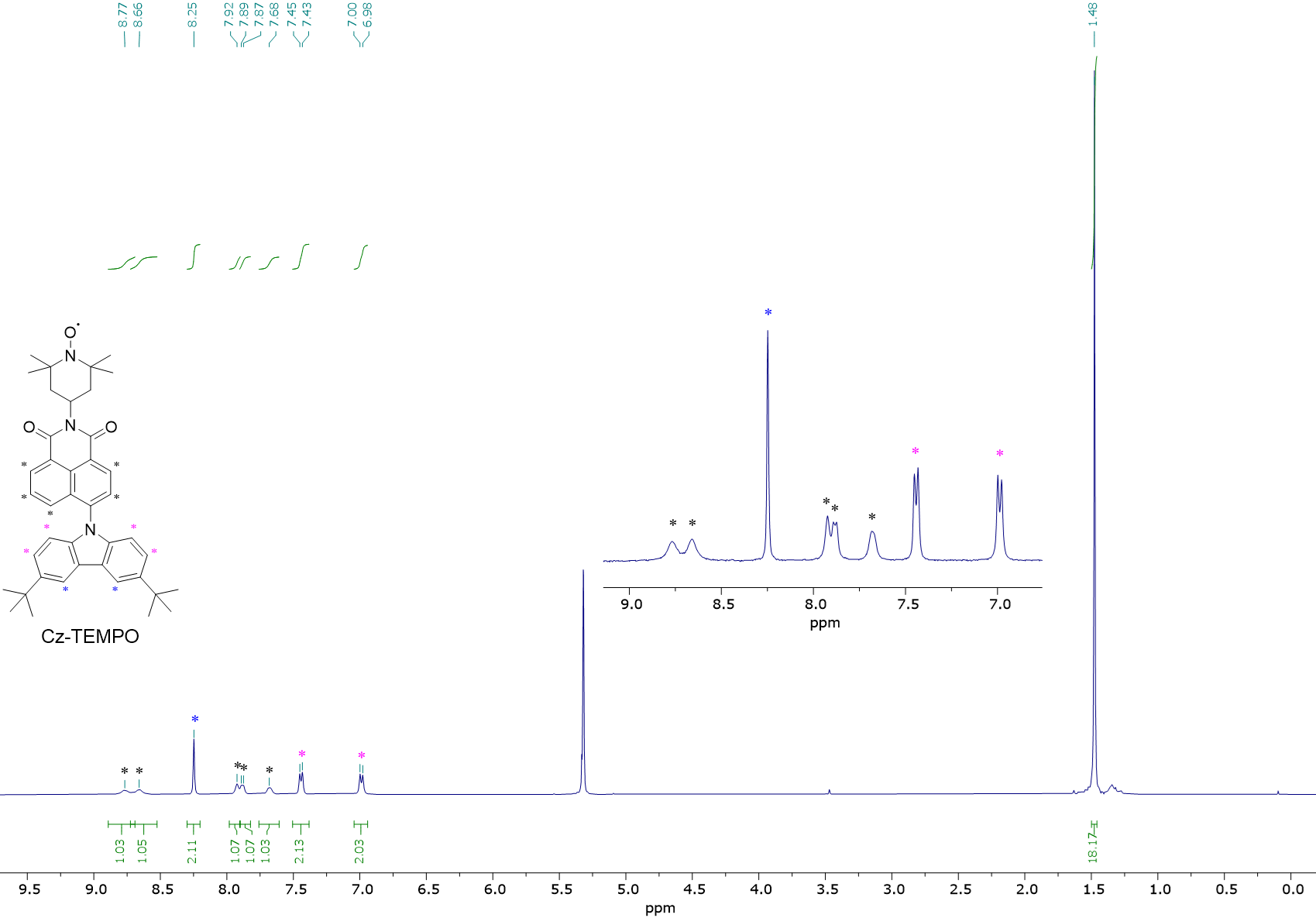


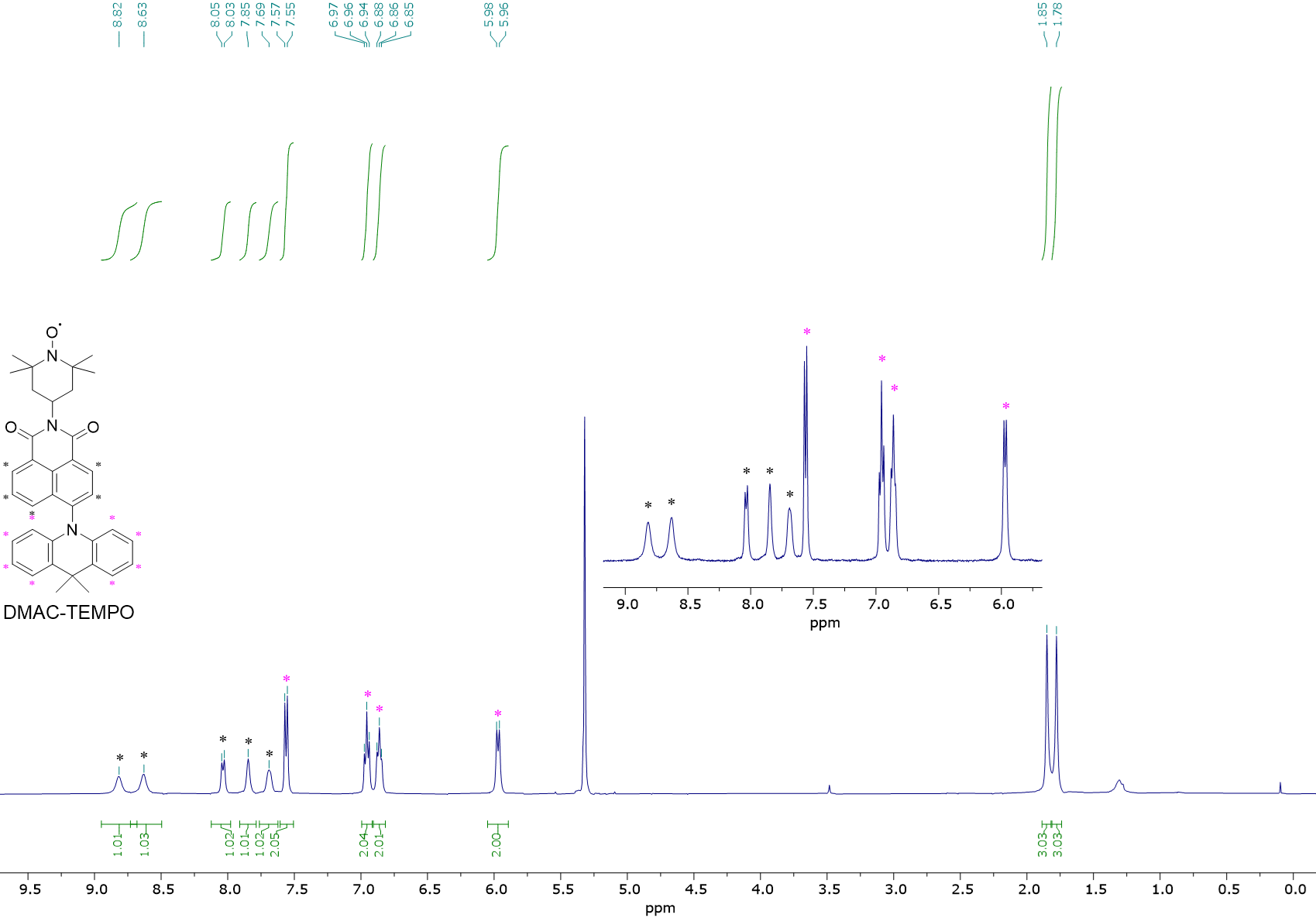


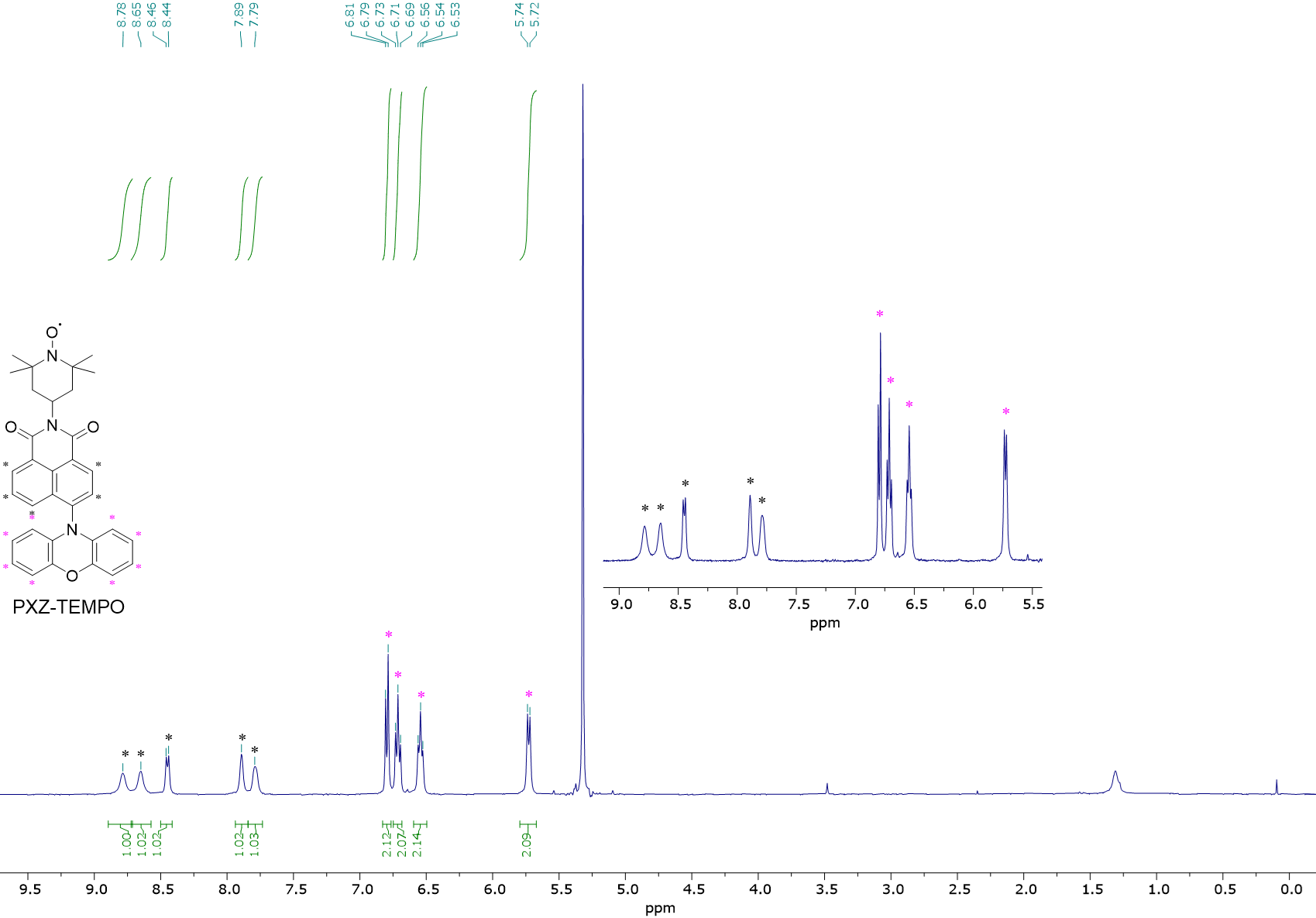


# SI References

[1] S. Ge, B. Li, X. Meng, H. Yan, M. Yang, B. Dong, Y. Lu, *Dyes Pigments* **2018**, *148*, 147.

[2] G. Türkmen, *J. Mol. Struct.* **2022**, *1266*, 133448.

[3] Y. Nakayama, N. Yokoyama, H. Nara, T. Kobayashi, M. Fujiwhara, *Adv. Synth. Catal.* **2015**, *357*, 2322.

[4] W. Zeng, H.-Y. Lai, W.-K. Lee, M. Jiao, Y.-J. Shiu, C. Zhong, S. Gong, T. Zhou, G. Xie, M. Sarma, K.-T. Wong, C.-C. Wu, C. Yang, *Adv. Mater.* **2018**, *30*, 1704961.

[5] Y. Qiu, H. J. Eckvahl, A. Equbal, M. D. Krzyaniak, M. R. Wasielewski, *J. Am. Chem. Soc.* **2023**, *145*, 25903.

[6] M. Mayländer, O. Nolden, M. Franz, S. Chen, L. Bancroft, Y. Qiu, M. R. Wasielewski, P. Gilch, S. Richert, *Chem. Sci.* **2022**, DOI 10.1039/D2SC01899C.

[7] G. M. Sheldrick, *Acta Crystallogr. Sect. Found. Adv.* **2015**, *71*, 3.

[8] C. F. Macrae, I. Sovago, S. J. Cottrell, P. T. A. Galek, P. McCabe, E. Pidcock, M. Platings, G. P. Shields, J. S. Stevens, M. Towler, P. A. Wood, *J. Appl. Crystallogr.* **2020**, *53*, 226.

[9] A. L. Spek, *Acta Crystallogr. Sect. C Struct. Chem.* **2015**, *71*, 9.

[10] A. L. Spek, *Acta Crystallogr. D Biol. Crystallogr.* **2009**, *65*, 148.

[11] J. B. Gerken, S. S. Stahl, *ACS Cent. Sci.* **2015**, *1*, 234.

[12] J. R. Harbridge, S. S. Eaton, G. R. Eaton, *J. Magn. Reson.* **2003**, *164*, 44.

[13] S. L. Bayliss, D. W. Laorenza, P. J. Mintun, B. D. Kovos, D. E. Freedman, D. D. Awschalom, *Science* **2020**, *370*, 1309.

[14] A. Mena, S. K. Mann, A. Cowley-Semple, E. Bryan, S. Heutz, D. R. McCamey, M. Attwood, S. L. Bayliss, *Phys. Rev. Lett.* **2024**, *133*, 120801.

[15] S. Gorgon, K. Lv, J. Grüne, B. H. Drummond, W. K. Myers, G. Londi, G. Ricci, D. Valverde, C. Tonnelé, P. Murto, A. S. Romanov, D. Casanova, V. Dyakonov, A. Sperlich, D. Beljonne, Y. Olivier, F. Li, R. H. Friend, E. W. Evans, *Nature* **2023**, *620*, 538.
